# Biotransformation and biological fate of magnetic iron oxide nanoparticles for biomedical research and clinical applications

**DOI:** 10.1039/d5na00195a

**Published:** 2025-03-24

**Authors:** Carlos Jacinto, Yasir Javed, Gabriel Lavorato, Wilson A. Tarraga, Blessed Isaac C. Conde, Juan Manuel Orozco, Agustin S. Picco, Joel Garcia, Carlos Sato Baraldi Dias, Sonia Malik, Surender Kumar Sharma

**Affiliations:** a Nano-Photonics and Imaging Group, Institute of Physics, Universidade Federal de Alagoas 57072-900 Maceió AL Brazil cjacinto@fis.ufal.br; b Department of Physics, University of Agriculture Faisalabad Pakistan; c Instituto de Investigaciones Fisicoquímicas Teóricas y Aplicadas (INIFTA), Faculdad de Ciencias Exactas, Universidad Nacional de La Plata – CONICET Diagonal 113 y 64 1900 La Plata Argentina; d Department of Chemistry, De La Salle University Manila Philippines joel.garcia@dlsu.edu.ph; e Institute for Photon Science and Synchrotron Radiation (IPS), Karlsruhe Institute of Technology (KIT) Hermann-von-Helmholtz-Platz 1 Eggenstein-Leopoldshafen 76344 Germany; f Physiology, Ecology & Environmental Laboratory (P2e), University of Orléans 45067 France soniamalik@babafaridgroup.edu.in; g Department of Biotechnology, Baba Farid College Bathinda 151001 India; h Department of Physics, Central University of Punjab Bathinda 151401 India surender.sharma@cup.edu.in; i Department of Physics, Federal University of Maranhão São Luís 65080-805 Brazil

## Abstract

Safe implementation of nanotechnology-based products in biomedical applications necessitates an extensive understanding of the (bio)transformations that nanoparticles undergo in living organisms. The long-term fate in the body is a crucial consideration because it governs potential risks for human health. To accurately predict the life cycle of nanoparticles, their fate after administration into the body—including their (bio)transformations, persistence, and biodegradation—needs to be thoroughly evaluated. Magnetic iron oxide nanoparticles (MIONPs) can enter the body through various routes, including inhalation, ingestion, dermal absorption, and injection. Microscale and nanoscale studies are performed to observe nanomaterial biotransformations and their effect on clinically relevant properties. Researchers are utilizing high-resolution TEM for nanoscale monitoring of the nanoparticles while microscale follow-up approaches comprise quantification tools at the whole organism level and the molecular level. Nanoparticle–cell interactions, including cellular uptake and intracellular trafficking, are key to understanding nanoparticle accumulation in cells and organs. Prolonged accumulation may induce cell stress and nanoparticle toxicity, often mediated through oxidative stress and inflammation. In this review article, the journey of nanoparticles in the body is depicted and their biotransformations and final fate are discussed. Immunohistochemical techniques are particularly valuable in tracking nanoparticle distribution within tissues and assessing their impact at the cellular level. A thorough description of a wide range of characterization techniques is provided to unveil the fate and biotransformations of clinically relevant nanoparticles and to assist in their design for successful biomedical applications.

## Abstract 2

Safe implementation of nanotechnology-based products in biomedical applications necessitates an extensive understanding of the (bio)transformations that nanoparticles undergo in living organisms.

## Graphical abstract

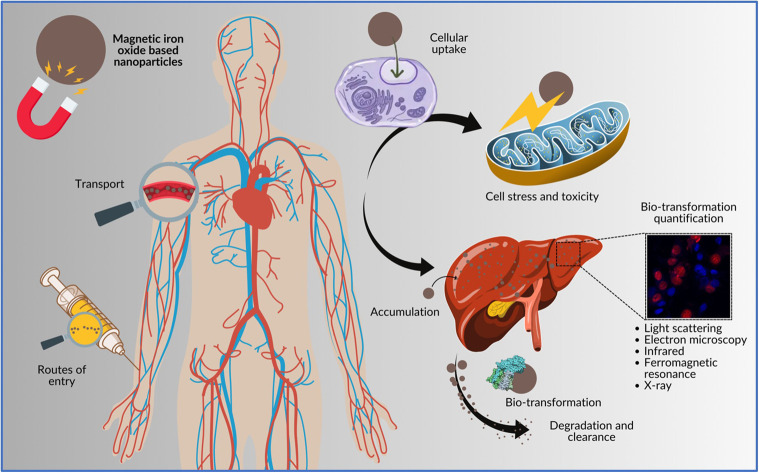

## 1. Introduction

For the implementation of engineered nanoparticles (NPs), the development of nanotechnology-based products may provide a sustainable alternative to traditional materials. However, this development faces major knowledge gaps and limitations regarding the environmental effects and potential health hazards posed by engineered NPs. The most exciting aspect that has emerged from the field of nanomedicine is the incorporation and utilization of NPs to solve technical issues in modern science, chiefly in biomedical and medical applications.^1,2^ In medical biology, the application of NPs leverages their interaction with cellular machinery and potential access to previously unreachable targets, such as the brain. NPs play a promising role in various biomedical fields such as cancer therapy, hyperthermia, gene delivery, magnetic resonance imaging, tissue repair, cell tracking, and drug delivery.^3^ The advancement of NPs in the field of nanomedicine highlights the need for assessing their potential toxicity along with the fate of nanomaterials *in vivo*.^4,5^

In the context of potential risks, the life cycle of NPs represents a comprehensive overview of their possible impacts. The life cycle of engineered NPs can be assessed based on their role in specific applications. The impact of NPs depends on their exposure scenarios, aging, and transformations within the products. Acute effects of NPs are evaluated through response/exposure assays; however, their biotransformations are less thoroughly investigated.^6^ Additionally, a reduction in size to the nanoscale greatly enhances particle reactivity. *In vivo* processing of NPs involves biotransformations, bioassimilation, biodegradation, elimination, and persistence mechanisms due to dynamic and complex interactions with various components in the biological media.^7,8^

Biological media continuously remodel the properties and identities of the NPs due to various types of interactions. Different types of molecules present in biological fluids reshape the surfaces of NPs, leading to aggregation, enzymatic attack, opsonization, and degradation. These remodeling mechanisms regulate NP transport in physiological media, potential toxicity, and cellular internalization.^9^ The main challenge is to track the complex interactions and characterize NPs during these processes *in vivo*. NPs are transformed and transported by various kinetic processes, which are generally considered to be slow.^10^

A major concern related to the NPs is their potential long-term persistence inside the body, which can cause chronic inflammatory reactions due to the presence of foreign substances. The central issue, therefore, is to analyze the life cycle of NPs within the body, from initial exposure through to complete assimilation and elimination. In some cases, rapidly degradable and highly reactive NPs are preferred, while in others, persistent, inert, and stable NPs are required to remain intact within living organisms.^11,12^ Additionally, by-products formed through NP degeneration may cause unexpected biological reactions. On the other hand, the accumulation of non-degradable NP products might disrupt autophagic and degradative pathways and saturate lysosomal compartments, which are essential for degrade proteins. Although nanotechnologists can control the synthesis mechanisms to adjust the shape, size, properties, and organization of NPs, the *in vivo* life cycle remains under active debate and discussion.^13,14^

The majority of studies focus on investigating the behavior of NPs over shorter timeframes (hours or days) following exposure, while longer exposure durations (months or years, up to complete elimination) remain ambiguous. The body must eventually degrade or eliminate these particles. Comprehensive investigations require time and face numerous methodological challenges in tracing NP residues *in vivo* over extended periods. In this review article, we discuss the common transformations and clearance routes that iron oxide-based NPs undergo during biomedical applications. Here, *transformations* are defined as alterations to the NP coating, primary particles, or changes resulting from agglomeration. Particles may also undergo additional transformation processes, such as surface oxidation or dissolution.

### 1.1. Magnetic iron oxide nanoparticles (MIONPs) in biomedicine

Most biomedical applications of iron oxide of NPs are based on magnetite (Fe_3_O_4_) and maghemite (γ-Fe_2_O_3_), which crystallize in a spinel cubic lattice (space group *Fd*3̄*m*).^15^ Magnetite is a mixed-valence iron oxide with the unit formula (Fe^3+^)_Td_ [Fe^2+^Fe^3+^]_Oh_O_4_^2−^, where divalent and half of the trivalent cations occupy octahedral (Oh) sites, while the other half occupy tetrahedral (Td) sites. Both magnetite and maghemite are Néel ferrimagnets, and the saturation magnetization is slightly larger for the former (92 compared to 82 A m^2^ kg^−1^).^16^ On the other hand, maghemite is a polymorph of ferric oxide (γ-Fe_2_O_3_) that is generally obtained after the oxidation of magnetite at temperatures below 300 °C. Its unit formula is (Fe^3+^)_Td_[Fe_5/3_^3+^*V*_1/3_]_Oh_O_4_^2−^, where *V* indicates additional vacancies in Oh sites, and its magnetocrystalline anisotropy is significantly reduced compared to magnetite.^16^ Under atmospheric conditions, hematite (α-Fe_2_O_3_) is the thermodynamically stable oxide at room temperature,^17^ but both maghemite and magnetite can be kinetically stabilized phases since the conversion to hematite requires a full transformation of the oxygen lattice from a cubic to a hexagonal phase which occurs above 300 °C.^1^ In turn, the conversion of magnetite NPs into maghemite is based on the diffusion of Fe cations within the FCC oxygen lattice *via* a thermally activated topotactic process. Due to their large surface-to-volume ratios, magnetite NPs have been shown to convert easily into maghemite at room temperature, both in dried solids and in aqueous colloids.^18–20^ In air or alkaline solution, the outward flow of Fe cations and electrons results in oxygen ionization, forming additional oxide layers at the NP surface,^19,21^ whereas in an acidic media, Fe(ii) is released into the solution.^10^ The diffusivity values that reflect the kinetics of this process depend on NP characteristics such as size, crystallinity, and the presence of crystal defects,^6^ and are also sensitive to the surrounding medium.^19^ For example, the epitaxial growth of a Zn-ferrite layer on Fe_3_O_4_ NPs has been shown to prevent magnetite oxidation.^22^ During the oxidation process, the spinel cubic crystal lattice can accommodate a full range of compositions between stoichiometric magnetite and pure maghemite. Since most synthesis methods are out of equilibrium, MIONPs with variable magnetite/maghemite ratios are typically produced. Due to the similarities in their crystal lattice, it is difficult to distinguish between them and therefore many studies refer to a Fe_3−*x*_O_4_ off-stoichiometric oxide. In this regard, there is some controversy on the Fe(ii) gradient in oxidized magnetite samples since some studies have shown homogeneous off-stoichiometric oxides,^23,24^ but others point to a magnetite/maghemite core@shell structure.^25^ Overall, the oxidation of magnetite is important to understand the biodegradation of iron oxides *in vivo*, which could be modulated by the composition and capping of the nanocrystals,^26^ thus additional efforts to characterize precisely their composition and structure in different media are necessary.^24^

The synthesis methods for magnetite or maghemite NPs rely on the use of Fe(ii) or Fe(iii) precursors and the redox control of the synthesis medium. Broadly speaking, the preparation routes include physical methods such as mechanical milling, laser ablation, and vapor deposition, whereas typical chemical methods involve co-precipitation, thermal decomposition, solvothermal, polyol routes, or magnetotactic bacteria-based methods. Comprehensive reviews on these methods can be found (*e.g.*, in ref. 27 and 28).

**Fig. 1:**
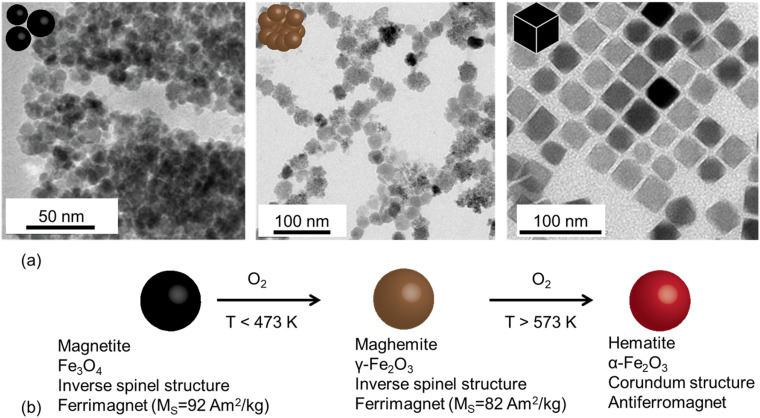
(a) TEM images of typical iron oxide nanomaterials: polydisperse NPs prepared by co-precipitation routes (left), multicore NPs (center), and monodisperse nanocubes prepared by thermal decomposition (right), (b) chemical transformations of magnetite NPs in the presence of oxygen at different temperatures and main properties of the associated iron oxides. Reproduced from ref. 36 under a Creative Commons (CC BY 3.0) License; reproduced from ref. 37 under a Creative Commons (CC BY 4.0) License; reproduced from ref. 38 with permission from American Chemical Society, copyright 2019.

The co-precipitation method is one of the simplest and most widely employed techniques for the preparation of magnetite and maghemite NPs^29,30^ (Fig. 1). It consists of the precipitation of the oxide by the addition of a base to an acidic solution of Fe(ii) and Fe(iii) cations under an inert atmosphere. The pH of the reaction, the strength of the base, and the reaction temperature are key parameters that determine the NP size, shape, and dispersion.^27^ Compared to other methods, neither expensive nor toxic reagents are required, nor temperatures above 100 °C. However, its main drawback is the broad size distribution, which has been attributed to the complex pathways that can lead to the formation of the nanocrystals.^29,31^ The importance of pH gradients in the reactor^32,33^ and the velocity of the base addition^33^ are important factors, and the precise nucleation and growth mechanism is still under discussion.^34^ NPs prepared by the co-precipitation route are dispersible in water, but coating with other molecules such as polyvinyl alcohol (PVA) or citrate can help to increase their colloidal stability.^35^

Other synthesis methods facilitate the production of multi-core NPs or magnetite chains, which are particularly interesting for biomedical applications because of their magnetic and photothermal properties.^39,40^ Multi-core NPs or nanoflowers can be produced through the polyol route, which consists of the hydrolysis of Fe alkoxide complexes in solutions of chelating alcohols such as diethylene glycol.^41^ In addition, chains of magnetite nanocrystals are synthesized by magnetotactic bacteria, making them particularly interesting due to the magnetic response that arises from the interaction between the NPs.^39^

Thermal decomposition methods, on the other hand, enable the production of highly crystalline NPs with precise control of their size and morphology.^42,43^ These methods, however, are based on a more complex synthesis framework, that usually require expensive reagents, and yield lower amounts of products. Iron precursors (Fe(iii) acetylacetonate, Fe-pentacarbonyl, or Fe-oleate) are decomposed in a high-boiling point solvent (such as benzyl ether or 1-octadecene) in the presence of oleic acid. Many studies have shown that wüstite domains (a paramagnetic iron oxide with Fe_1−*y*_O unit formula) can be formed as a result of such a reducing atmosphere, resulting in a decrease in magnetic properties. To circumvent this issue, some reports have employed oxidation agents that can favor the formation of maghemite nanocrystals^44^ and focused on controlling the redox activity of the solvents to stabilize magnetite NPs.^45^ The thermal decomposition method is particularly useful for producing core@shell^46^ and anisotropic NPs, including cubes^42^ and octopods,^47^ which provides outstanding control over the crystallinity and size of the nanocrystals. However, these methods yield oleate-capped NPs that form stable colloids in non-polar solvents, and surface functionalization methods are required for most biomedical applications. As a result, different molecules can be anchored at the NP surface, which would impact on the biotransformation and biological fate of MIONPs. Three different approaches are generally employed: (i) a ligand-exchange process to replace the oleate capping with a hydrophilic one, (ii) the formation of an inorganic shell on the iron oxide and (iii) the encapsulation of oleate-capped NPs into amphiphilic polymers that form micelle-like structures. In the first case, simple molecules such as tetramethyl ammonium hydroxide (TMAOH) or 2,3-dimercaptosuccinic acid (DMSA) can be used to remove the oleate capping and produce electrostatically stabilized colloids.^48,49^ More complex structures based on polyethylene glycol ligands firmly anchored to the oxide through catechol molecules have also been reported.^50,51^ Alternatively, ligand-exchange methods can be followed by the formation of core@shell inorganic structures, and methods to grow biocompatible SiO_2_ layers on iron oxides have been reported.^52^ On the other hand, amphiphilic polymers such as poly(maleic anhydride-*alt*-1-octadecene) (PMAO) or phospholipid-PEG ligands can interdigitate with oleate molecules and leave exposed hydrophilic entities,^53,54^ circumventing the requirement for an oleate-removal step. In these cases, the polymer coating can control the surface reactivity and determine the biodegradation pathway.^43^

Techniques such as physical adsorption, covalent attachment, and ligand exchange offer varying levels of stability and control over surface properties, while advanced methods like polymer coating and silica encapsulation enhance biocompatibility and functional versatility. These strategies enable improved dispersion, targeted delivery, and compatibility with biomedical and environmental systems. On the other hand, click chemistry has emerged as a powerful and highly efficient method for surface modification of MIONPs due to its simplicity, specificity, and bioorthogonality. This technique typically utilizes azide–alkyne cycloaddition reactions, which proceed under mild conditions and yield high-purity products.^55,56^ A review by Yi and colleagues discussed applications of click chemistry in nanoparticle modification together with their targeted delivery. Several recent studies highlight the utility of click chemistry for conjugating biomolecules, such as phytochemicals,^57^ peptides,^58^*etc.*, to the MIONP surface with exceptional precision. This method not only ensures strong covalent binding but also maintains the biological activity of the attached molecules. Furthermore, click chemistry is advantageous for creating multifunctional surfaces, allowing simultaneous integration of targeting ligands, imaging agents, and therapeutic payloads. By leveraging the modularity and robustness of click reactions, researchers have developed highly tailored MIONPs that excel as biosensors,^55,59^ in targeted drug delivery,^60^ magnetic resonance and near-infrared fluorescence imaging,^60,61^ and magnetic hyperthermia.^58^ The versatility and reproducibility of click chemistry make it a cornerstone technique for advancing the functionalization and application of MIONPs in cutting-edge nanotechnology.

Current biomedical applications of iron oxide NPs require careful control of their size, shape, composition, and surface functionalization. Many of these applications are based on the use of NPs to deliver heat locally *via* the application of external stimuli. This can be exploited, for example, in magnetic hyperthermia or photothermal therapies,^62,63^ heat-triggered drug delivery,^64^ and cryopreservation technologies.^65^ Large heating efficiencies are often achieved by heating mediators with a large saturation magnetization and precise control of their effective anisotropy.^52,66^ This can be realized by tuning the particle shape or by doping the iron oxide with metal cations such as Co, Mn, or Zn.^52^ In this sense, magnetite NPs are preferred over maghemite, but the oxidation to maghemite should be considered since it is responsible for a decrease in the magnetic moment and a change in the effective anisotropy,^67^ particularly for *in vivo* conditions.^68^ Interparticle magnetic interactions, which are modulated by the size, shape, and surface capping of the NPs, can also play a crucial role in determining the effective magnetic anisotropy of iron oxide colloids through the formation of NP chains during the application of alternating magnetic fields.^66^

On the other hand, the efficiency of iron oxides in photothermal therapies relies on precise control of the magnetite fraction and the density of crystal defects in the iron oxide lattice.^40^ Compared to magnetic hyperthermia, it has been demonstrated that the heating power per gram of material is superior under photothermal conditions.^63^ However, the latter is limited by the penetration depth of light; therefore, magnetic hyperthermia may be more advantageous for achieving remote energy transduction.

Iron oxides have also been proposed as contrast agents for magnetic resonance imaging (MRI),^69^ a widely used technique in clinical diagnosis. The local field generated by MIONPs can modulate the relaxation process by shortening the relaxation time of protons located in their surroundings, thus increasing the contrast in the image. Such contrast enhancement depends on the interaction between water molecules and NPs, which is influenced by many factors such as saturation magnetization, NP shape, and hydrodynamic diameter.^70^ Compared to *T*_1_ contrast agents (mostly based on complexes of paramagnetic ions such as Gd^3+^ and Mn^2+^), MIONPs reduce the transverse relaxation time (*T*_2_); therefore, a combination of a high saturation magnetization and ligands that favor the diffusion of water in the vicinity of the magnetic cores is desirable.^69^ It has been shown that Fe_3_O_4_ and metal-doped ferrite nanocubes^69,71^ or octapod-shaped NPs^47^ provide very large relaxivities and therefore excellent *T*_2_ contrast enhancement effects due to the strongly inhomogeneous local magnetic fields induced by their anisotropic shape.

### 1.2. Routes of entry of magnetic iron oxide nanoparticles into the body

MIONPs can enter the body through various routes, influencing their biodistribution, cellular interactions, and potential effects. One common route of entry for MIONPs is through inhalation, where airborne NPs can be inhaled and reach the respiratory system, potentially leading to deposition in the lungs and subsequent translocation to other organs.^72^ Inhalation exposure to MIONPs is of particular concern due to the potential for respiratory effects and systemic distribution of NPs throughout the body.^72^ However, for biomedical applications, there are currently no Food and Drug Administration (FDA)-approved MIONPs specifically for inhalation administration. Research in the field of inhalation therapies using magnetic NPs is ongoing, and these NPs show promise for applications such as drug delivery and treatment of respiratory diseases due to their ability to be directed and controlled by external magnetic fields.^73–76^ However, none have reached the stage of FDA approval for this specific route of administration. Most currently approved MIONPs are used for intravenous, oral, or intramuscular administration. It is crucial to consider the potential implications for respiratory health and the systemic distribution of these NPs. While MIONPs have shown promise in various biomedical applications, their inhalation raises concerns regarding pulmonary toxicity and the potential for systemic absorption. Studies such as those by Hosseini *et al.*^77^ have highlighted the influence of MIONPs on neural stem cell proliferation and gene expression in the hippocampus following ischemia or reperfusion, indicating a potential impact on cellular processes.

Additionally, injection is a commonly used route for the administration of NPs in biomedical applications. Studies have investigated the biodistribution and clearance of MIONPs following injection, highlighting their accumulation in various organs and tissues, as will be discussed in the next sections of this review. The injection route can influence the systemic distribution and targeting efficiency of MIONPs for applications such as drug delivery and imaging 62. The FDA has approved several MIONPs for intravenous administration, primarily for use as MRI contrast agents and for treating iron deficiency anemia (Table 1). As of now, there are no FDA-approved MIONPs specifically for intramuscular administration. Most research and clinical applications of MIONPs focus on intravenous administration, with key uses in MRI, drug delivery, and hyperthermia treatment for cancer. These NPs are designed to take advantage of their magnetic properties for targeted delivery and imaging, but their administration is primarily *via* intravenous routes rather than intramuscular. The development and application of MIONPs for biomedical purposes are still actively being researched, focusing on improving their biocompatibility, stability, and targeted delivery capabilities.^73,78–80^

**Table 1:** Current FDA-approved MIONPs for intravenous administration

MIONP	Indication	Description	Reference
Ferumoxytol (Feraheme®; Rienso® in the EU)	Initially approved for the treatment of iron deficiency anemia in patients with chronic kidney disease and later gained attention as an MRI contrast agent	Coated with carboxymethyl-dextran and has a size range of 17–31 nm; approval dates back to 2009	Huang *et al.*^76^
Ferumoxtran-10 (Combidex®)	Approved for use as an MRI contrast agent	Coated with dextran and sized between 15–30 nm; although it faced some regulatory hurdles, it was approved for specific clinical applications	Huang *et al.*^76^
Ferumoxide (Feridex®)	Used as an MRI contrast agent for liver imaging and other applications	Coated with dextran and has a larger size range of 50–100 nm	Huang *et al.*^76^
Ferucarbotran (Resovist®)	Used as an MRI contrast agent, particularly for liver imaging	Coated with carboxydextran and has a size of approximately 80 nm	Huang *et al.*^76^

Another entry route for MIONPs is oral ingestion, where NPs can be ingested through food, water, or other sources. Research has highlighted the importance of understanding the gastrointestinal fate of MIONPs upon oral exposure, including their absorption, distribution, and potential toxicity in the digestive system.^81^ The size, shape, and surface properties of MIONPs can influence their behavior upon oral ingestion, impacting their interactions with biological tissues and cells.^81^ Kiyani *et al.*^81^ investigated the potentially harmful effects of FeO NPs (MIONPs) taken orally on the skeletal muscle, liver, and kidney of BALB/c mice (a laboratory-bred strain of the house mouse). Compared to the control group, histological evaluation of the liver, kidney, and skeletal muscle tissue in the three experimental groups showed no signs of necrosis, inflammation, or degenerative alterations. At dosages up to 500 mg kg^−1^ body weight for 21 days, there was no histological evidence of any harmful effects on the liver, kidney, or skeletal muscle of BALB/c mice when spherical-shaped MIONPs (average diameter of 50 nm) were ingested orally. These results are consistent with some studies, yet other investigations have documented the harmful consequences of MIONPs. This implies that the degree of toxicity shown by MIONPs can vary depending on their size, shape, and mode of exposure. Currently, the FDA has approved ferumoxsil (trade name Lumirem® in the US and GastroMARK® in the EU) for oral administration. This MIONP is coated with siloxane and is primarily used for gastrointestinal imaging. Ferumoxsil was designed to enhance the contrast of the gastrointestinal tract in MRI scans by providing clear imaging of the bowel.^76,82^

Furthermore, dermal exposure represents another route through which MIONPs can enter the body. Skin contact with products containing MIONPs, such as cosmetics or topical formulations, can lead to the absorption of NPs through the skin barrier. Recent studies have demonstrated the effectiveness of superparamagnetic iron oxide NPs (SPMIONPs) in enhancing the transdermal delivery of chemotherapeutic molecules for skin tumors in hairless albino C57BL/6 mice.^83^ For example, maghemite NPs (γ-Fe_2_O_3_) with an average core diameter of 25 nm were sterically stabilized using a blend of macro-RAFT (reversible addition–fragmentation chain transfer) copolymers, consisting of 90% poly(ethylene glycol)methyl ether(methoxy polyethylene glycol, MPEG)-end and 10% NH_2_-end, synthesized *via* RAFT polymerization. Each polymer comprises a block of 10 units of monoacryloxyethyl phosphate to secure MPEG or polyacrylamide to the surface of SPIONs. Additionally, the polymer includes either a block of 40 acrylamide units and 17 units of poly(ethylene glycol) (PEG) (in MPEG-end-copolymer) or a block of 60 acrylamide units (in NH_2_-end copolymer) for steric stabilization. The combination of topical 5-fluorouracil and these SPIONs markedly decreased tumor growth compared to using 5-fluorouracil alone (*p* = 0.0072), whereas the application of SPIONs by themselves showed no significant effect (*p* = 0.96). This indicates that properly formulated NPs have been shown to increase the penetration of cytotoxic drugs like 5-fluorouracil (a hydrophilic, negatively charged molecule that struggles to penetrate the hydrophobic, negatively charged stratum corneum) into skin tumors, thereby improving therapeutic outcomes by enhancing transdermal penetration and modulating host–tumor interactions.^83^ Understanding the dermal penetration and distribution of MIONPs is essential for assessing their safety and potential effects on skin health. The transdermal administration of MIONPs offers a promising approach with significant potential in various biomedical applications; however, there are still no FDA-approved MIONPs specifically for transdermal administration.

The systemic distribution and targeting effectiveness of MIONPs for imaging and medication delivery are strongly influenced by the route of administration. Studies have indicated that the injection method can significantly influence the biodistribution, targeting specificity, and overall effectiveness of MIONPs in many biological contexts. For example, systemically injected CoMn-IONP nanoclusters efficiently accumulated in tumors *via* passive targeting, utilizing the enhanced permeability and retention (EPR) effect.^84^ This study highlighted how the route of injection can influence the accumulation of NPs in target tissues, emphasizing the importance of systemic delivery for effective tumor targeting. Similarly, Xie *et al.*^85^ showed that the delivery and intratumoral distribution of ultra-small MIONPs were primarily promoted by the EPR effect, underscoring the significance of systemic administration for tumor imaging and therapy.

Colby *et al.*^86^ demonstrated the importance of the route of administration for the material-based targeting strategy of expansile NPs. They found that intraperitoneal injection led to tumor-specific accumulation, whereas rapid clearance occurred with intravenous administration. This study exemplifies how the choice of injection route can impact the targeting specificity of NPs, influencing their distribution and retention in tumor tissues. Additionally, Thin *et al.*^87^ highlighted that intravenous cell administration is a common route for delivering cells but can lead to entrapment in the pulmonary capillary bed, affecting their distribution to distal tissues. Voxel-based (a three-dimensional counterpart to a pixel) dosimetry was conducted by Gupta *et al.*^88^ using small animal single photon emission computed tomography (SPECT)/CT (computed tomography) imaging of mice to evaluate the distribution of IONP-conjugated ^177^Lu-labeled folic acid following systemic administration. After injection, the mean absorbed dose at the organ level using the medical internal radiation dose (MIRD) schema was calculated, and the radioactivities of all three radiotracers, ^177^Lu-IONP-folate (>10 nm), ^177^Lu-folate, and ^177^Lu-MIONPs, were mainly and immediately accumulated in the liver (voxel-based absorbed doses = 0.96 ± 0.05, 0.76 ± 0.07, and 1.09 ± 0.20 Gy MBq^−1^, respectively) and kidneys (1.01 ± 0.17, 2.46 ± 0.50, and 0.52 ± 0.08 Gy MBq^−1^, respectively). About half of the renal absorbed dose decreased significantly after comparing ^177^Lu-IONP-folate with when the ^177^Lu-folate alone. Although no other routes of administration were conducted for comparison, their study demonstrated how the route of injection could impact the biodistribution and dosimetry of NPs, providing insights into their targeting efficiency *in vivo*.

Taken together, the route of injection significantly influences the systemic distribution and targeting efficiency of MIONPs for drug delivery and imaging applications. By selecting the optimal administration route, researchers can enhance NP biodistribution, targeting specificity, and overall therapeutic efficacy, ultimately improving their clinical translation and utility in various biomedical settings.

### 1.3. Nanoparticle–cell interactions

It is essential to consider the mechanisms involved in the cellular uptake and internalization of MIONPs to understand how they are introduced into cells. One crucial aspect is the surface chemistry of the NPs, which can influence their interaction with cell membranes and uptake pathways.^89^ NPs can enter cells through specific and nonspecific endocytosis pathways, such as phagocytosis and pinocytosis, depending on their surface properties and functionalization. Altering the surface chemistry of NPs can impact their internalization routes, highlighting the importance of surface modifications in mediating cellular uptake.^90^ One standard method of introducing MIONPs into cells is through receptor-mediated endocytosis, especially for NPs sized 20 to 200 nm, where NPs are internalized into the cytosol *via* specific cell membrane receptors.^91^ This process involves binding NPs to cell surface receptors, triggering their uptake into cells through endocytic pathways. By utilizing specific ligands or antibodies that target cell-specific receptors, the internalization of NPs into desired cell types can be enhanced, enabling precise delivery and targeting.^90^ This approach allows for the selective introduction of NPs into cells based on receptor expression profiles, offering a tailored strategy for cellular uptake. The following highly expressed receptors are some examples of MIONP targets.

• Transferrin receptor (TfR): MIONPs have been demonstrated to induce irreversible changes in protein conformation upon interaction with transferrin.^92^ These NPs can be directed to transferrin receptors through various strategies, such as utilizing iron chelators to upregulate the receptor before administering targeted NPs.^93^ Transferrin, an iron-transporting protein, is pivotal in this mechanism. It comprises bilobal protein shells with dual domains in each lobe, harboring an interdomain iron-binding cleft.^94^ The transferrin binding to the transferrin receptor initiates receptor-mediated endocytosis, resulting in the internalization of transferrin within endocytic vesicles and the subsequent release of iron from the protein.^95^ Additionally, the upregulation of transferrin receptors has been linked to cellular iron uptake and processes like apoptosis in endothelial cells.^96^ The transferrin receptor-dependent iron uptake mechanism has been implicated in various cellular processes, including drug-induced apoptosis.^96^

• Epidermal growth factor receptor (EGFR) and human epidermal growth factor receptor 2 (HER2/neu): MIONPs targeted to the epidermal growth factor receptor (EGFR) have shown promising applications in cancer therapy and imaging. When conjugated with specific ligands targeting EGFR, these NPs can induce various cellular responses. For instance, MIONPs, covalently coated with carboxymethyldextran, targeted to EGFR have been demonstrated to selectively induce lysosomal membrane permeabilization in cancer cells overexpressing EGFR under an alternating magnetic field.^97^ Additionally, NPs conjugated with single-chain anti-EGFR antibodies have been developed for tumor targeting and imaging, showcasing the potential of these targeted NPs in cancer theranostics.^98^ Also, SPMIONPs conjugated with recombinant human epidermal growth factor have been studied for magnetic resonance imaging contrast enhancement in malignant brain tumors.^99^ The internalization of MIONPs targeting EGFR has been shown to induce cell death in tumoral cells upon exposure to an alternating magnetic field.^100^ Moreover, the conjugation of magnetic NPs with EGFR-targeting ligands has been utilized for effective gene therapy in hepatocellular carcinoma and glioblastoma stem cells.^101,102^ Furthermore, NPs conjugated with single-chain anti-HER2 (human epidermal growth factor receptor 2) antibodies have been developed for tumor targeting and imaging, showcasing the potential of these targeted NPs in cancer theranostics.^98^

• Folate receptor (FR): interestingly, recent studies have focused on developing various types of NPs for targeted drug delivery to cancer cells overexpressing the folate receptor. Angelopoulou *et al.*^103^ developed folic acid-functionalized iron oxide condensed colloidal magnetic clusters to selectively deliver doxorubicin to tumor cancer cells. Folate receptors, particularly folate receptor alpha (FOLR1), have been identified as promising targets for delivering doxorubicin to cancer cells, enhancing the efficacy of this chemotherapeutic agent.^104^ Kaittanis *et al.*^105^ investigated the interaction of polyacrylic-acid-coated magnetic nanosensors with cancer cells expressing the folate receptor, showing that high-folate magnetic relaxation nanosensors outperformed low-folate counterparts in single cancer cell detection. Yin *et al.*^106^ prepared folate receptor-targeted multifunctional fluorescent magnetic NPs with cores containing iron oxide nanocrystals and shells with multimodal imaging capability. Bhattacharya *et al.*^107^ evaluated ultradispersed nanoconjugates for bimodal imaging as *T*_2_-weighted negative contrast MR imaging agents in folate-overexpressed cells. Microfluidic-modified folic acid-hybridized Fe_3_O_4_@SiO_2_ magnetic NPs have been designed to bind specifically to folate receptors expressed on tumor cells, enabling the capture and isolation of circulating tumor cells.^108^ Pluronic F127-folate-coated SPIONs have been investigated for their ability to target cancer cells expressing folate receptors for cancer diagnosis using MRI.^109^ The specific targeting of folate receptors by these NPs was confirmed through flow cytometry analysis and confocal laser scanning microscopy, highlighting their potential for precise cancer imaging. Moreover, pH-sensitive NPs with a superparamagnetic core and folic acid conjugation (FA-doxorubicin-iron oxide NPs [FA-DOX@MIONPs]) have been developed for the targeted delivery of doxorubicin to breast cancer cells (MCF-7, BT549, and MD-MBA-231) overexpressing folate receptors, and showing no toxicity against healthy organs *in vivo* in mice.^110^

• Prostate-specific membrane antigen (PSMA): MIONPs, particularly SPIONs, have attracted significant attention in prostate cancer research due to their potential applications in imaging and therapy. By conjugating these NPs with specific ligands, such as prostate-specific membrane antigen (PSMA), researchers have aimed to enhance the specificity and efficacy of cancer treatments.^111,112^ To understand the mechanism of MIONPs targeting PSMA, it is crucial to probe the interaction between these NPs and PSMA. Panday *et al.* discussed the utilization of MIONPs for tumor imaging by injecting a specific concentration of these NPs into the body and guiding them to the tumor site using an external magnet, providing contrast for imaging.^112^ This method showcases the capability of MIONPs to be directed to specific locations, which could be pivotal for targeting PSMA-expressing prostate cancer cells. Sun *et al.*^113^ also synthesized PSMA-targeted NPs loaded with doxorubicin and tanshinone (P-N-DOX/TAN, size: 139.7 ± 4.1 nm, *ζ* = 11.2 ± 1.6 mV) by conjugating a PSMA-targeted ligand to the NPs. This conjugation strategy illustrates the specificity achieved by linking PSMA-targeting ligands to NPs, potentially facilitating the targeted delivery of therapeutic agents to PSMA-expressing cells. This approach could be adapted to incorporate MIONPs for targeted drug delivery to prostate cancer cells expressing PSMA. The mini-review by Farina *et al.*^114^ discusses NPs as a platform for the targeted delivery of miRNA inhibitors or mimics specifically to prostate tumor cells to inhibit cancer progression. By conjugating PSMA-targeting ligands to these NPs, enhancing the targeted delivery of therapeutic agents to prostate cancer cells may be feasible. Integrating MIONPs into such systems could offer dual functionality for imaging and targeted therapy of PSMA-positive prostate cancer cells.

• Integrins (*e.g.*, αvβ3 integrin): to understand the mechanism of MIONPs targeting integrins, exploring their potential interactions and implications on integrin-mediated processes is essential. Integrins are cell surface receptors that play a crucial role in cell adhesion, signaling, and migration, making them significant targets for various biomedical applications. In a study by Li *et al.*,^115^ integrin αvβ6-targeted MR molecular imaging of breast cancer in a xenograft mouse model was investigated using peptide cFK-9 to N-amino (–NH_2_)-modified ultrasmall superparamagnetic iron oxide (USPIO) NPs that are also capable of providing negative 
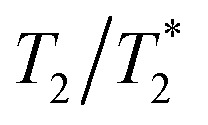
-weighted contrast in MRI. This application demonstrates the potential of MIONPs in targeting specific receptors like integrins for imaging purposes.

• Cluster of differentiation 44 (CD44): CD44 is a cell surface glycoprotein involved in cell adhesion, migration, and signaling, making it a significant target for various biomedical applications. In a study by Su *et al.*,^116^ anti-CD44 antibody-modified Fe_3_O_4_ SPIONs were used to target and eliminate head and neck squamous cell carcinoma stem cells to undergo apoptosis in an alternating magnetic field, showing 33.43% inhibitory ratio of the treated group. This application highlights the specificity and efficacy of CD44-targeted magnetic NPs in cancer therapy. Additionally, Nasr *et al.*^117^ focused on the effects of nanoprobe morphology on cellular binding and inflammatory responses, specifically utilizing hyaluronan-conjugated iron oxide magnetic nanoworms for magnetic resonance imaging of atherosclerotic plaques. This study emphasizes the role of CD44, a receptor for hyaluronic acid, in targeting atherosclerotic plaques in rabbits, showcasing the potential of magnetic NPs in CD44-mediated imaging applications. The mechanism of MIONPs targeting CD44 involves their unique magnetic properties, potential for functionalization, and interactions with CD44-expressing cells. By leveraging these properties, researchers can explore innovative approaches for targeted drug delivery, imaging, and therapeutic interventions in the context of CD44-mediated processes in various biological settings.

Another mechanism for introducing MIONPs into cells is through fluid-phase endocytosis, where NPs are engulfed by cells in a nonspecific manner. This process involves the internalization of extracellular material through the formation of vesicles, allowing for the uptake of NPs into cells regardless of specific receptor interactions. Usually, uncoated NPs lead to irreversible endocytosis.^118^ However, Ding *et al.*^119^ highlight how the size, shape, and protein corona of NPs also influence cellular uptake mechanisms, including endocytosis pathways such as clathrin-mediated and caveolin-mediated endocytosis. For example, star-shaped NPs adopt clathrin- and caveolin-mediated endocytosis pathways. On the other hand, 80 nm NPs undergo macropinocytosis due to their larger size. By leveraging fluid-phase endocytosis, researchers can achieve broad cellular uptake of NPs, facilitating their delivery to various cell types for biomedical applications. The process of endocytosis, crucial for internalizing NPs, has been the subject of multiple studies. Caveolin-1 and cell division cycle 42 (CDC42) have been identified as vital mediators of endocytosis of silica-coated MIONPs in HeLa cells, underscoring the significance of surface characteristics in NP uptake.^120^ Surface functionalization of magnetite SPIONs with ligands like insulin has been demonstrated to facilitate receptor-mediated targeting, thereby preventing nonspecific endocytosis and enhancing specific cellular uptake.^118^ Furthermore, the impact of surface charge on the internalization of carboxymethyl dextran-coated MIONPs with different degrees of substitution, ranging from 38 to 5 –COOH per chain groups per chain, has been illustrated, with fluid-phase endocytosis inhibited by specific concentrations of inhibitors.^121^ It is crucial to consider their cellular interactions and uptake mechanisms to investigate the fluid-phase endocytosis of MIONPs. A study by Sruthi *et al.*^122^ delves into the cellular interactions of mPEG_2000_-Si- and rhodamine-functionalized SPMIONPs on oligodendrocytes, highlighting their uptake *via* endocytosis and the subsequent intracellular Fe content increase. This finding underscores the importance of understanding how these NPs are internalized by cells, shedding light on their potential applications in targeted drug delivery and cellular imaging. Moreover, the synthesis and characterization of MIONPs play a pivotal role in determining their behavior during endocytosis. Qiao *et al.*^123^ discuss the advantages of Fe_3_O_4_ NPs in drug delivery applications due to their inertness, biocompatibility, and ease of detection, which can influence their interaction with cells during endocytosis. By controlling the size and shape of these NPs through methods like coprecipitation and *in situ* magnetic separation, researchers can tailor their properties for efficient cellular uptake and intracellular trafficking.^124^ Furthermore, the physicochemical characteristics of MIONPs, as highlighted by Etemadi *et al.*,^125^ are crucial in understanding their endocytosis behavior and potential toxicity profiles for endocytosis. The unique magnetic properties of these NPs, coupled with their surface chemistry, can influence their interactions with cell membranes and internalization pathways, impacting their fate within cells.^125^ This knowledge is essential for designing NPs that can be effectively taken up *via* endocytosis by cells without causing adverse effects. In the context of cancer research, the study by Krzyminiewski *et al.*^126^ on NP endocytosis in cancer cells using electron spin resonance spectroscopy sheds light on the mechanisms of cellular uptake and intracellular localization of MIONPs, specifically magnetite Fe(ii,iii) oxide core functionalized with organic unit containing nitroxide radical 4-hydroxy-TEMPO. Understanding how these NPs interact with cancer cells can provide insights into their potential use in targeted cancer therapies and imaging modalities, emphasizing the importance of studying their endocytosis pathways.

Phagocytosis represents another pathway through which MIONPs can be introduced into cells, particularly immune cells like macrophages.^127^ In this process, cells engulf large particles, including NPs, by forming phagosomes that internalize the particles for subsequent processing. By exploiting the phagocytic activity of immune cells, researchers can target specific cell populations for NP delivery, offering opportunities for immunotherapy and targeted drug delivery strategies. The ability to harness phagocytosis for NP uptake provides a valuable avenue for modulating immune responses and delivering therapeutic agents to immune cells. To investigate the phenomenon of phagocytosis involving MIONPs, it is crucial to consider their interactions with cells, particularly phagocytic cells like macrophages. A study by Dalzon *et al.*^128^ discusses the influences of NP characteristics on cellular responses, focusing on MIONPs of 100 and 200 nm in size and their uptake by mouse macrophage cell line J774A1. Additionally, the research by Lin *et al.*^129^ on remote magnetic control of autophagy in B-lymphoma cells (A20) using MIONPs, CdSe/ZnS quantum dots, and MIONPs-CdSe/ZnS quantum dots composite particles (all of approximately 10 nm particle size), sheds light on the intracellular degradation process following phagocytosis. It was shown that B-lymphoma cancer cells phagocytosed a considerable amount of MIONPs. When an external magnetic field was applied in a particular direction, the MIONPs aggregated in the cells. They caused light chain protein 3 (LC3) proteins to migrate from the cytoplasm to the nucleus during autophagy, producing a significant amount of proinflammatory cytokines like interleukin 6 (IL-6). Although the mechanisms of the pathways involved in this action were not explicitly elucidated, this study illustrates how MIONPs can modulate cellular processes like autophagy, highlighting their potential for targeted therapeutic interventions through phagocytosis-mediated uptake. In the context of cancer research, the study by Balk *et al.*^127^ on cellular uptake and toxicity of superparamagnetic lauric acid-coated MIONPs in head and neck cancer cell lines emphasizes the importance of assessing the biocompatibility and potential cytotoxic effects of these NPs following phagocytosis. This research contributes to understanding how MIONPs interact with cancer cells through phagocytosis, informing their use in cancer diagnostics and therapy.

Additionally, studies have demonstrated the use of magnetic force-mediated internalization to introduce magnetic MIONPs into cells.^130^ By applying external magnetic fields, the movement and localization of NPs can be manipulated, guiding their internalization into target cells. This approach enables the precise targeting and retention of NPs within cells, offering a controlled method for introducing NPs into specific cell populations. Magnetic force-mediated internalization provides a versatile strategy for enhancing the uptake of NPs and facilitating their intracellular delivery for various biomedical applications. An *in silico* model of Wirthl *et al.*^131^ explores the capture of superparamagnetic MIONPs in tumor spheroids in the presence of flow, highlighting the role of a cylindrical magnet in exerting magnetic forces on the NPs. This underscores how magnetic forces can influence the behavior and localization of NPs within complex biological environments, offering insights into the dynamics of NP internalization under the influence of magnetic fields. Introducing MIONPs into cells involves a range of mechanisms, including receptor-mediated endocytosis, fluid-phase endocytosis, phagocytosis, and magnetic force-mediated internalization.^132^ By understanding these pathways and optimizing NP surface properties, researchers can tailor the cellular uptake of NPs for specific applications in drug delivery, imaging, and therapy. The diverse strategies for introducing NPs into cells offer opportunities for precise targeting, controlled delivery, and enhanced therapeutic outcomes in biomedical research and clinical practice.

### 1.4. Cell stress and cellular toxicity

The cellular uptake of MIONPs involves various mechanisms, primarily endocytosis pathways and receptor-mediated internalization, as discussed in the previous section of this paper. In endocytosis, MIONPs are engulfed by cells through the plasma membrane, forming vesicles that transport the NPs into the cell. Additionally, macropinocytosis allows for bulk uptake of MIONPs by cells, particularly at varying high NP concentrations, depending on the size of the macropinosome; relatively large particles are typically engulfed through phagocytosis since phagosomes are sufficiently large, usually exceeding 250 nm.^133,134^ On the other hand, receptor-mediated internalization involves specific ligand–receptor interactions, where surface-modified MIONPs bind to cell surface receptors, leading to their internalization through receptor-mediated endocytosis. This mechanism enables targeted delivery of MIONPs to specific cell types or tissues by conjugating targeting ligands, such as antibodies or peptides, to the NP surface. These cellular uptake mechanisms are influenced by factors including IONP properties, cell type, and the local microenvironment, which are crucial for optimizing NP design for various biomedical applications.

**Fig. 2:**
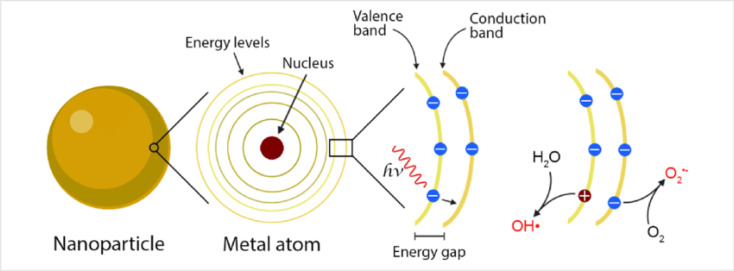
The mechanism of reactive oxygen species (ROS) production by MIONPs. The process begins with the NP, which contains metal atoms at its core. These metal atoms have a nucleus surrounded by discrete energy levels. When the NP absorbs energy in the form of photons (*hν*), electrons in the metal atoms can be excited from the valence band to the conduction band, leaving an energy gap between these two bands. This electron excitation creates a situation where the conduction band has an excess electron. In contrast, the valence band has a positive charge due to the missing electron (referred to as a hole). The positively charged hole in the valence band can interact with water molecules (H_2_O), forming hydroxyl radicals (OH˙). Meanwhile, the electron in the conduction band can react with oxygen molecules (O_2_) to form superoxide anions (O_2_˙^−^). These ROS, such as hydroxyl radicals and superoxide anions, are highly reactive and can cause oxidative damage to biological systems. The generation of ROS by MIONPs is a crucial aspect of their behavior and can have significant biological implications, depending on the context of their use or exposure. Reproduced from ref. 137 under a Creative Commons (CC BY) Licence from the Beilstein Institute for the Advancement of Chemical Sciences, Copyright [2020] CC-BY adapted from Correa *et al.*^138^ © 2020 CC-BY.

Intracellularly, the fate of MIONPs includes subcellular localization and potential interactions with cellular organelles. To comprehend the cellular responses to MIONPs, including oxidative stress, inflammation, genotoxicity, and apoptosis, a thorough review of the literature sheds light on the potential effects of these NPs on biological systems. Studies, such as those by Malabanan *et al.*,^135^ have indicated that MIONPs can induce cellular changes, including DNA damage, chromosome condensation, and micronuclei formation, by producing reactive oxygen species (ROS), ultimately leading to cell death. This underscores the potential genotoxic effects of MIONPs on cellular DNA integrity. MIONPs have been extensively studied for their ability to induce the production of ROS within biological systems. Research by Feng *et al.*^136^ have shown that MIONPs can lead to oxidative stress and apoptosis in cells, with polyethylenimine (PEI)-coated MIONPs exhibiting higher uptake and cytotoxicity through mechanisms such as ROS production. The mechanism of ROS production by MIONPs involves various pathways (Fig. 2) which can lead to oxidative stress and cellular damage.

Sharma *et al.*^137^ and Li *et al.*^139^ have highlighted the role of MIONPs in generating ROS, particularly in the context of cancer treatment and bacterial eradication. These NPs have been shown to catalyze ROS generation, which can contribute to their antibacterial and anticancer properties. The influence of the oleic acid coating on the magnetic susceptibility and Fenton reaction-mediated ROS generation by MIONPs, as discussed by Aadinath & Muthuvijayan,^140^ emphasizes the importance of surface modifications in ROS production. The Fenton reaction, mediated by MIONPs, is responsible for generating ROS, which can contribute to the antibacterial activity of these NPs. Additionally, the study by Yi *et al.*^141^ suggests that the catalytic activity of MIONPs can lead to ROS generation through intraparticle electron transport, enhancing their efficacy as nanozymes. The review by Li *et al.*^142^ discusses how MIONPs can induce oxidative stress through reactions that produce highly reactive hydroxyl or hydroperoxy radicals. These radicals can promote DNA damage, lysosomal dysfunction, and mitochondrial malfunction, contributing to cellular oxidative stress. These radicals can directly damage DNA by causing modifications to its structure, such as strand breaks, base modifications, and DNA–protein cross-links. This DNA damage can lead to mutations and genomic instability, contributing to cellular dysfunction and potentially carcinogenesis. Hydroxyl radicals can induce lysosomal dysfunction by disrupting lysosomal membranes and impairing lysosomal enzyme function, leading to the release of lysosomal contents into the cytoplasm and subsequent cell damage. Additionally, hydroxyl radicals can also target mitochondria, where they cause oxidative damage to mitochondrial membranes and proteins, disrupt electron transport chain function, and induce mitochondrial DNA mutations. These mitochondrial dysfunctions can lead to decreased ATP production, further increased ROS generation, and ultimately, cell death. The study by Meng *et al.*^143^ further supports the role of MIONPs in increasing ROS generation and oxidative stress, highlighting the time-course cellular effects triggered by these NPs.

*In vitro*, Ansari *et al.*^144^ have emphasized the genotoxic and oxidative stress-inducing potential of MIONPs, leading to DNA damage and apoptosis in various cell lines, including cancer cells. Bardestani *et al.*^145^ have demonstrated that free Fe ions released from MIONPs can trigger apoptosis pathways in exposed cells by depolarizing the cell membrane, disrupting membrane potential, and modulating the expression of key regulatory proteins like B-cell lymphoma 2 (BCL-2), BCL-2-associated X protein (BAX), and BCL-2-associated death promoter (BAD), along with the activation of caspase-3. Regulatory proteins such as BCL-2, Bax, and BAD play crucial roles in cellular processes, particularly in apoptosis regulation. BCL-2, an anti-apoptotic protein, inhibits cell death by preventing the release of cytochrome c from the mitochondria. Conversely, BAX and BAD are pro-apoptotic proteins that promote cell death by inducing mitochondrial outer membrane permeabilization and releasing apoptogenic factors.^146^ These proteins belong to the BCL-2 family, which regulates the intrinsic pathway of apoptosis, balancing anti-apoptotic (*e.g.*, BCL-2) and pro-apoptotic (*e.g.*, BAX, BAD) members.^147^ The balance between these proteins, such as BAX, BAD, and BCL-2, is critical in determining cell fate during apoptosis.^148^ Additionally, the phosphorylation status of BAD, including key regulatory residues like Ser112, Ser136, and Ser155, influences its activity and its role in promoting apoptosis.^149^ Moreover, interactions between regulatory proteins, such as the binding of phosphorylated BAD to 14-3-3 proteins, can modulate apoptotic signaling pathways.^150^ Incorporating the concept of MIONPs, it is noteworthy that free Fe ions released from MIONPs can trigger apoptosis pathways in exposed cells. In cancer studies, dysregulation of BCL-2 family proteins, including BAD, has been linked to promoting cell survival and tumorigenesis. For instance, the upregulation of pro-apoptotic proteins like BAD can induce apoptosis in cancer cells, highlighting their potential as therapeutic targets.^151^ Additionally, the phosphorylation of BAD by kinases like Akt can modulate its function and impact cell survival and proliferation in cancer.^152^ The intricate interplay among regulatory proteins like BCL-2, BAX, and BAD is essential for maintaining cellular homeostasis and determining cell fate under various conditions. These proteins are involved in complex signaling pathways governing apoptosis, emphasizing their significance in both normal physiological processes and disease states.

**Fig. 3:**
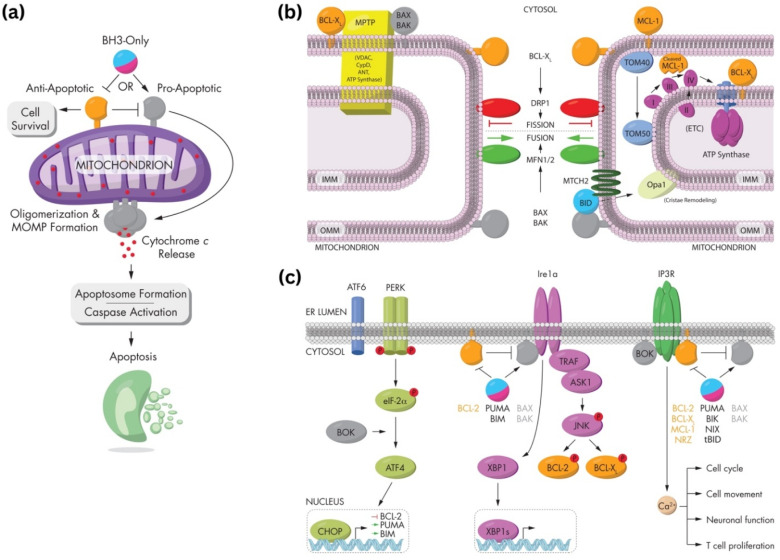
Regulation of BAX/BAK activation and oligomerization by BCL-2 family proteins. (a) The mitochondrial pathway of apoptosis, a critical cellular process that balances survival and programmed cell death. At the center of this pathway are BCL-2 family proteins, which are divided into three main groups: pro-apoptotic, anti-apoptotic, and BH3-only proteins. BH3-only proteins serve as upstream sensors of cellular stress and damage, which can activate either pro-apoptotic or anti-apoptotic proteins depending on the signals received. Pro-apoptotic proteins promote mitochondrial outer membrane permeabilization (MOMP) by oligomerizing and forming pores in the mitochondrial membrane. This results in the release of cytochrome c into the cytosol, a pivotal step in the apoptotic cascade. Cytochrome c interacts with apoptotic protease-activating factor-1 (Apaf-1) to form the apoptosome, leading to the activation of caspases—proteolytic enzymes that dismantle the cell in a controlled manner. In contrast, anti-apoptotic proteins counteract this process by inhibiting the activity of pro-apoptotic proteins, thereby maintaining mitochondrial integrity and promoting cell survival. The balance between these opposing forces determines whether the cell undergoes apoptosis or survives. (b) Mitochondrial dynamics and their regulation in apoptosis and metabolism. The outer (OMM) and inner (IMM) membranes are key sites for fission, fusion, and permeability transitions. Fission, mediated by DRP1, is balanced by fusion-promoting proteins MFN1/2 and OPA1, maintaining mitochondrial morphology and function. Anti-apoptotic BCL-XL stabilizes membranes, while pro-apoptotic BAX and BAK promote permeabilization. The mitochondrial permeability transition pore (MPTP), involving VDAC, ANT, and ATP synthase, responds to stress and can trigger cell death if dysregulated. TOM40 and TOM50 import proteins critical for the electron transport chain (ETC) and ATP synthesis. BID and cleaved MCL-1 regulate apoptosis and remodeling, while OPA1 optimizes cristae structure for ETC function. The balance of fission, fusion, and permeabilization is essential for energy, survival, and cell death. (c) Unfolded protein response (UPR) and its link to apoptosis and cellular regulation, focusing on key pathways activated during endoplasmic reticulum (ER) stress. The UPR is mediated by three ER membrane sensors: ATF6, PERK, and IRE1α, each contributing to stress adaptation or apoptosis depending on the severity of stress. PERK phosphorylates eIF2α, reducing global protein synthesis while allowing selective translation of ATF4. ATF4 upregulates pro-apoptotic factors like CHOP, PUMA, and BIM while suppressing anti-apoptotic BCL-2, promoting apoptosis under prolonged stress. IRE1α, through its RNase activity, splices XBP1 mRNA to produce XBP1s, aiding in adaptive responses. However, if stress persists, IRE1α interacts with TRAF and ASK1 to activate JNK, leading to phosphorylation of BCL-2 and BCL-XL, shifting the balance toward apoptosis. IP3R, involved in calcium signaling, can further amplify stress by facilitating mitochondrial calcium uptake, contributing to apoptotic processes mediated by BAX, BAK, and BH3-only proteins like PUMA, BIM, and BID. Reproduced from ref. 155 with permission from Springer Nature, Copyright 2017.

The upregulation of pro-apoptotic proteins and the downregulation of anti-apoptotic proteins can lead to caspase-3 activation and the initiation of the apoptotic cascade. Yaguchi *et al.*^153^ have elucidated how the Bcl-2 family proteins, including BCL-2, B-cell lymphoma-extra-large (BCL-XL), BAD, and BAX, regulate mitochondrial membrane potential and influence caspase-3 activation. The dynamic interactions and relative abundance of these proteins impact the mitochondrial apoptotic pathway, leading to caspase-3 activation and cell death. The modulation of these regulatory proteins, as demonstrated by He *et al.*^154^ and in a detailed review by Gross and Katz,^155^ can upregulate pro-apoptotic factors, activating the caspase cascade and promoting apoptosis (Fig. 3). Understanding the roles of these regulatory proteins is crucial for developing targeted therapies and interventions that modulate apoptotic pathways for therapeutic purposes. These findings highlight the potential of MIONPs to induce programmed cell death through various molecular mechanisms.

Interestingly, the study by Wang *et al.*^156^ has indicated that the photothermal effect of MIONPs can induce autophagy in cancer cells in a laser dose-dependent manner, suggesting a potential mechanism for cell death induction. Additionally, Basaki *et al.*^157^ have associated maternal exposure to MIONPs with ferroptosis in the brain, indicating the potential neurotoxic effects of these NPs. The literature suggests that MIONPs have the potential to induce oxidative stress, inflammation, genotoxicity, and apoptosis in various cell types, underscoring the importance of understanding the cellular responses to these NPs for both therapeutic and safety considerations. Further research is needed to elucidate the precise mechanisms underlying these cellular responses and to optimize the use of MIONPs in biomedical applications while minimizing potential adverse effects on cellular health.

The properties of MIONPs, such as size, shape, surface charge, and surface coating, play a significant role in influencing cellular responses and interactions. Studies, such as those by Feng *et al.*^136^ and Mulens-Arias *et al.*,^158^ have highlighted the impact of MIONP size and surface coating on biological effects, including cellular uptake, distribution, and toxicity. The size of MIONPs can influence cellular responses, with smaller NPs potentially enhancing cytokine secretion and cellular interactions compared to larger particles.^158^ Additionally, the surface coating of MIONPs can modulate cellular responses, affecting factors such as cellular uptake, biocompatibility, and immunomodulation.^136^ The surface charge of MIONPs, as discussed by Kimura *et al.*,^159^ can also influence cellular interactions and pharmacokinetics. Positively charged NPs may exhibit different cellular uptake and biodistribution patterns compared to negatively charged particles, impacting their biological effects. For example, amphiphilic PEI-coated modified MIONPs can capture negatively charged molecules like DNA or RNA through electrostatic interactions.^160^ PEI, a positively charged material, modifies the surface of MIOPs to be positively charged. This positive charge on the MIONPs surface attracts and binds to the negatively charged phosphate groups found in the backbone of DNA or RNA molecules. These modified NPs can serve as MRI-visible carriers for gene or drug delivery, as well as probes for tracking cells.^136^ Interestingly, macrophages in the liver extensively absorbed highly charged micellar NPs (*e.g.*, PEG^5k^-CA_8_ NPs), whether positively or negatively charged, but NPs with a slight negative charge showed the least clearance by macrophages and achieved the highest uptake by tumors.^160^ However, there is still no study demonstrating the conjugation of these types of ligands to MIONPs.

Moreover, the choice of coating material can affect properties such as hydrodynamic size, *ζ*-potential, shape, and curvature, ultimately impacting cellular responses, interactions, and biocompatibility.^143,161^ The time-course effects of MIONPs on cellular responses depend on properties such as hydrodynamic size, surface coating, and incubation time, underscoring the importance of these factors in determining cellular effects. Additionally, the colloidal stability of MIONPs, influenced by factors like particle size, surface chemistry, and aqueous conditions, can affect cellular responses and interactions. The influence of surface modifications on MIONPs, as explored by Karimi *et al.*,^161^ can impact cellular responses, with different coatings leading to varying levels of oxidative stress and biological effects. Coatings can alter the surface functionality of MIONPs, affecting interactions with biomolecules and cellular uptake. Moreover, the relaxivity of MIONPs for MRI, as investigated by Ge *et al.*,^162^ can be tuned by particle size and surface coating, highlighting the importance of these properties in biomedical applications.

**Fig. 4:**
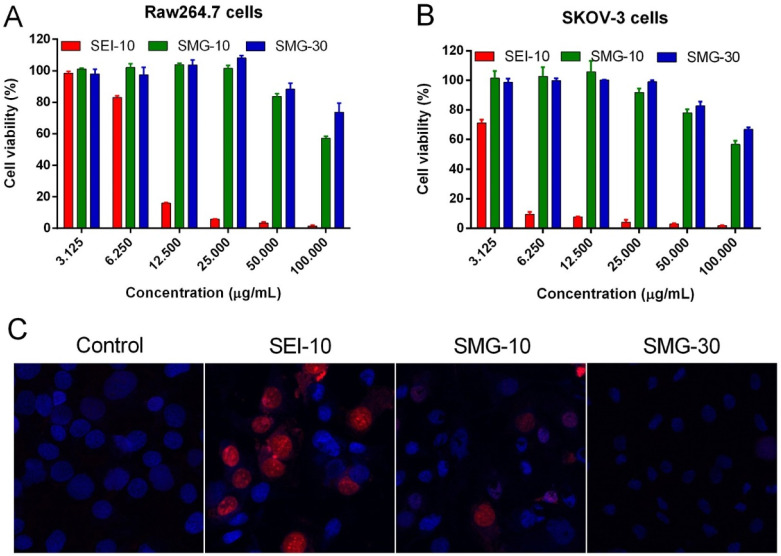
The *in vitro* cytotoxicity of various MIONPs coated with either SEI or PEG from the study of Feng *et al.*^136^ The cell viability of RAW264.7 macrophages (A) and SKOV-3 cells (B) was evaluated using the MTS assay after 48 h of treatment with different concentrations of MIONPs. (C) Representative fluorescent microscopic images illustrate the mode of cell death induced by different MIONPs in SKOV-3 cells. The cells were treated with SEI-10 (17 nm, 5 μg mL^−1^), SMG-10 (17 nm, 400 μg mL^−1^), or SMG-30 (36 nm, 400 μg mL^−1^) for 16 h, followed by staining with Hoechst 33342 (blue) and PI (red). Abbreviations SEI-10, SMG-10, and SMG-30 are based on their uncoated particle sizes. Reproduced from ref. 136 under a Creative Commons (CC-BY) Licence from Springer Nature, Copyright 2020.

NP size-dosage relationship can also influence the cellular toxicity of MIONPs (Fig. 4). For example, Feng *et al.*^136^ demonstrated that SEI-coated MIONPs (17 nm) exhibited higher cellular uptake, *in vitro* on SKOV-3 (human ovarian cancer cells) and *in vivo* in mice, compared to 30 nm ones and showed slight cytotoxicity only at high concentrations (>50 μg mL^−1^). In their study, most of the nuclei in SKOV-3 cells exhibited positive PI staining, indicating cell death after a 16 h exposure to 5 μg mL^−1^ of PEI-coated MIONPs (17 nm). However, only a small fraction of nuclei in cells treated with PEGylated MIONPs (17 nm) showed positive PI staining, even at 400 μg mL^−1^; PI staining in cells treated with larger PEGylated MIONPs (36 nm) was nearly negative, even at concentrations as high as 400 μg mL^−1^.

Moreover, Hansapaiboon *et al.*^163^ that CUR-2GE-Ch-MIONPs significantly increased cellular uptake and cytotoxicity towards breast cancer cells, indicating a dose-dependent effect on cellular toxicity. Furthermore, the study by Arias *et al.*^164^ highlighted that magnetite, a type of MIONP, led to concentration-dependent toxicity in human lung alveolar epithelial cells, showing that the concentration of MIONPs plays a crucial role in inducing cellular toxicity. Additionally, Ding *et al.*^165^ emphasized that surface chemistry and size greatly affect the magnetic properties, biodistribution, and toxicity of MIONPs, indicating that the dosage of MIONPs can impact their toxicity levels. In a study by Yang *et al.*,^166^ it was observed that MIONP administration reduced sperm quantity and quality in male mice in a dose-dependent manner, suggesting that higher dosages of MIONPs can have adverse effects on the reproductive system. This underscores the importance of considering dosage levels when assessing the potential toxicity of MIONPs on different biological systems. Moreover, the research by Casset *et al.*^167^ indicated that the internalization of MIONPs in macrophages resulted in a dose-dependent increase in toxicity, emphasizing the impact of dosage on cellular responses to MIONPs. Similarly, the study by Yoon *et al.*^168^ highlighted that the cytotoxicity of MIONPs has been linked to cellular uptake followed by the production of reactive oxygen species, indicating that higher dosages may lead to increased cellular damage. Taken together, these studies suggest that the dosage of MIONPs is critical in determining their cellular toxicity levels. Higher dosages of MIONPs may lead to increased cellular uptake, reactive oxygen species generation, and cytotoxic effects, highlighting the importance of carefully considering dosage levels when evaluating the potential toxicity of MIONPs across biological systems.

Several strategies have been proposed to address the cellular toxicity of MIONPs. One approach involves optimizing the coating of MIONPs to enhance biocompatibility. Generally, it is suggested that coating MIONPs with polyethylene glycols (PEG) can improve the biocompatibility of these NPs, making them more suitable for biomedical applications like gene delivery and MRI imaging.^136^ Similarly, Arias *et al.*^164^ highlighted that coating MIONPs with naturally occurring antioxidants (*e.g.*, phenolic acids) or with either natural or synthetic polymers—such as polyethylene glycol, poly(vinylpyrrolidone), polyvinyl alcohol, poly(lactic-*co*-glycolic acid), and chitosan—can reduce their toxic effects, emphasizing the importance of surface modifications in enhancing biocompatibility. Another strategy to mitigate cellular toxicity involves controlling the size and crystallinity of MIONPs. Jeon *et al.*^169^ emphasized that reducing crystallinity can decrease the saturation magnetization of MIONPs, promoting *T*_1_ contrast and potentially reducing their cytotoxicity. Furthermore, utilizing drug delivery systems that incorporate MIONPs has been proposed as a strategy to mitigate toxicity. Balabathula *et al.*^170^ developed a targeted nano drug delivery system using lyophilized MIONPs encapsulated with amphotericin B for treating systemic fungal infections, showcasing the potential of such systems to deliver therapeutic agents while minimizing toxicity.

In addition to surface modifications and drug delivery systems, antioxidants have been suggested as a means to mitigate MIONP toxicity. For example, Ansari *et al.*^144^ demonstrated that supplementation with thymoquinone could attenuate the genetic and oxidative damage induced by MIONPs in a dose-dependent manner. Similarly, Ogbezode *et al.*^171^ reviewed the use of green-synthesized MIONPs for biomedical applications, highlighting their low toxicity and biodegradability, which may help reduce adverse effects. Moreover, strategies involving the modulation of macrophage responses have been proposed to enhance the bactericidal activity of MIONPs. Yu *et al.*^172^ suggested fine-tuning the capacity of MIONPs to promote macrophage polarization towards a pro-inflammatory phenotype, which could be beneficial in conditions requiring robust immune responses, such as cancer and infectious diseases. This approach leverages the innate immune system to combat pathogens while minimizing toxicity. In summary, a combination of surface modifications, drug delivery systems, antioxidant supplementation, and immune modulation strategies can help mitigate the cellular toxicity of MIONPs. By optimizing the design and application of these NPs, researchers can improve their biocompatibility and therapeutic efficacy while minimizing adverse effects on cells and tissues.

Regulatory considerations for using MIONPs in biomedical applications are crucial to ensuring their safe and effective implementation. Several key aspects must be addressed to meet regulatory standards and guidelines. Firstly, the surface coating of MIONPs plays a significant role in determining their biocompatibility and toxicity profiles. Studies have shown that appropriate surface coatings are essential for the biomedical applications of MIONPs.^136^ Regulatory bodies often require detailed information on the composition and properties of NP coatings to assess safety and efficacy. Moreover, the unique properties of MIONPs—such as superparamagnetism, non-toxicity, biocompatibility, and chemical inertness—make them ideal for various biomedical applications.^173^ Regulatory agencies may require comprehensive data on the physicochemical characteristics of MIONPs to evaluate their suitability for specific medical uses. Additionally, the potential toxicity of MIONPs must be carefully considered in regulatory assessments. Research has indicated that MIONPs can trigger cellular damage, such as endoplasmic reticulum stress in hepatic cells, underscoring the importance of evaluating their safety profiles.^174^ Regulatory bodies may mandate thorough toxicity studies to assess the risks of using MIONPs in medical applications.

Future research should focus on elucidating the detailed molecular mechanisms underlying IONP-induced cellular stress and toxicity, including their impact on oxidative stress pathways, mitochondrial dysfunction, and DNA damage repair mechanisms. Additionally, there is a need to investigate the long-term effects of IONP exposure on cellular function and homeostasis, as well as the influence of MIONPs' physicochemical properties on their toxicity profiles. Comprehensive *in vivo* studies are essential to validate findings from *in vitro* models and to assess the biodistribution, pharmacokinetics, and systemic effects of MIONPs. Bridging the gap between preclinical studies and clinical applications is critical, addressing challenges such as NP clearance, immunogenicity, and off-target effects. By addressing these knowledge gaps, researchers can develop safer and more effective NP-based therapeutics for various biomedical applications.

## 2. Iron oxide nanoparticles in biological fluids

### 2.1. Protein corona formation and nanoparticle aggregation

When in contact with biological fluids (*e.g.*, plasma), NPs spontaneously adsorbed proteins on their surface. The adsorbed proteins forms what is known as *protein corona*.^175,176^ In addition, other biomolecules like lipids and sugars are also able to adsorb onto NPs. Owing to this, the more comprehensive term *biomolecular corona* is also used for indicating that the corona layer is formed by macromolecules (*i.e.*, proteins) and other small molecules (*e.g.*, lipids and sugars).^177^ Once formed, the protein corona constitutes a new interface between the NPs and the body and change the “biological identity” of the pristine NPs.^176^ Consequently, it has a tremendous impact on the NPs fate and their pharmacological, toxicological, therapeutic and diagnostic properties.^178^ Owing to this, in the last years, great efforts have been made to understand the relation between NPs characteristics such as constituent material, size, surface properties and coating material, and protein corona properties like thickness, composition and evolution through time.^179–181^

**Fig. 5:**
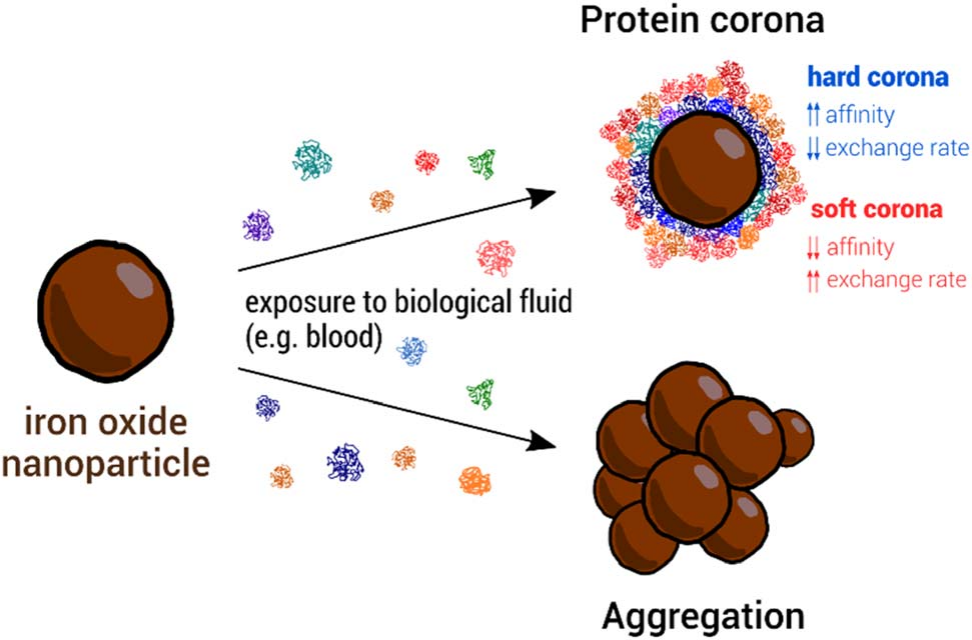
NP outcomes in biological fluids: upon contact with biological fluids containing proteins (*e.g.*, blood) NPs adsorbs proteins (represented as compact colored coils in the figure) from the media. The formed protein corona presents two different regions, one constituted by proteins with high affinity for the surface and low exchange rate with the medium, called *hard corona*, and another comprising protein with low affinity and high exchange rate, named *soft corona*. In parallel, NPs can aggregate in biological fluids. This can be a consequence of the high ionic strength exhibited by the latter and/or triggered by the adsorption of proteins. For the specific case of MIONPs, they also can aggregate owing to magnetic interactions.

The protein corona is composed of two regions: the hard and soft coronas.^176^ The hard corona consists of proteins with a high affinity for the NP surface and a low exchange rate with bulk media. This layer is thought to accompany the NP as it travels through various biological compartments in the body, significantly influencing its interactions with cells and extracellular components.^182,183^ Conversely, the soft corona is formed by proteins with lower affinity for the NP, which exhibit high exchange rates between their adsorbed and non-adsorbed forms. While the soft corona's impact on NP fate is generally considered less significant than that of the hard corona, this remains an area of active debate.^184–187^ The formation and distribution of proteins into the hard and soft corona are illustrated in Fig. 5. Furthermore, the protein corona is a highly dynamic structure that evolves over time.^188,189^ Initially, proteins with high media concentrations but low NP affinity are adsorbed. Over time, proteins at lower concentrations but with higher affinity gradually displace the initially adsorbed proteins. Known as the Vroman effect,^190^ this phenomenon can occur over a broad time range—from seconds to hours—depending on the NP and media conditions.^191^

In parallel, NP aggregation is another phenomenon frequently observed in biological fluids present *in vivo* like plasma or used for *in vitro* testing such as cell culture media (*e.g.*, DMEM, RPMI) supplemented with fetal bovine serum (FBS). It is schematized in Fig. 5, below protein corona. NP aggregation usually leads to the rapid clearance of the NP by the mononuclear phagocytic system (MPS), thus reducing the NP blood half-life (*t*_1/2_).^192^ As it is discussed below, the rapid clearance of NP aggregates severely alters the NP therapeutic and/or diagnostic efficiency. Besides, aggregation of NPs can affect NP biodistribution,^193^ alter toxicity profile^194^ and potentially trigger embolism events.^195^

Aggregation of NPs is a consequence of the loss of their colloidal stability which in such complex environments like plasma or cell culture media can be attributed to several reasons,^196^ the most important being:

• The high electrolyte concentration observed in these media causes the screening of the electrostatic repulsion between alike NPs. Then, weak attractive van der Waals forces dominate the force balance and NP tend to aggregate unless other repulsive interaction opposes to them (*e.g.*, steric repulsion provided by a polymer coating).

• Adsorption of proteins from media onto NP surface may reduce NP surface charge and consequently trigger NP aggregation. Also, protein adsorption might induce bridging interactions between NPs leading to the loss of colloidal stability. This phenomenon is more prone to happen at low protein concentrations, where NPs are partially covered by them. Conversely, the opposite behavior, *i.e.*, improvement of NP colloidal stability as a consequence of protein adsorption, has also been seen frequently. In this case, the stability increase has been attributed to the formation of a uniform protein layer surrounding the NP that provides steric stabilization to the colloid.

• In the case of magnetic NPs such as iron oxide-based ones, attractive magnetic forces are a destabilizing contribution to the colloidal stability.^197^ As it was explained above, the magnitude if these interactions greatly depend on NP size and shape.

**Fig. 6:**
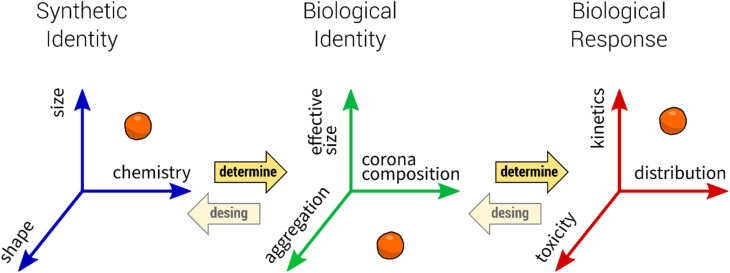
Scheme showing the relation between the *synthetic identity* of a NP (as prepared in the lab), its *biological identity* (when in contact with biological fluid) and the *biological response* that triggers (in the biological organism). For more details, see the text above. Reproduced from ref. 176 with permission from the Royal Society of Chemistry (RSC), Copyright 2012.

To recap, the synthetic identity of a NPs, encompassing aspects such as composition, size, shape, and surface chemistry, dictates its behavior in biological fluids, including the adsorption of proteins and other biomolecules, and maintenance of colloidal stability. This synthetic identity molds the biological identify of the NPs, effectively shaping how it is perceived by cells and various body compartments. Ultimately, this biological identity governs the biological and physiological responses elicited by the NP within the body. This concept, schematized by Walkey *et al.*^176^ a decade ago (see Fig. 6), holds the promise of a transformative perspective: as our comprehension of these interactions advances, it may become possible to reverse-engineer NP synthetic identities based on desired biological responses.

Single protein adsorption onto MIONPs and the impact on protein structure has been intensively investigated and probed to depend on NP nature (*i.e.*, hematite, maghemite, magnetite), surface coating and media properties, among other factors.^180^ For instance, iron saturated human transferrin (Tf) underwent irreversible conformational changes upon interaction with magnetite MIONPs.^198^ These changes were more pronounced upon exposure to bare MIONPs than polyvinyl alcohol (PVA) coated ones, and attributed to loss of Fe ions from the protein. Conversely, apo-Tf and partially iron saturated Tf were found to be (thermally) stabilized upon adsorption onto bare maghemite MIONPs.^199^ Upon interaction of lysozyme (from hen egg white) with bare MIONPs, no changes in the protein structure were observed at low NP concentrations while protein destabilization was detected at higher ones.^200^ This behavior was attributed to changes in the preferential hydration of protein molecules at low and high NP concentration. On the other hand, it was shown that MIONPs coated with trisodium citrate (TSC) and sodium triphosphate (STP) induced denaturation of lysozyme while polyethyleneglycol (PEG) coated ones did not.^201^ The loss of lysozyme native structure in the first two cases was ascribed to the Na^+^ diffusion to the core of the protein. The interaction of fibrinogen (Fb) with bare maghemite MIONPs led to perturbations in the secondary and tertiary structure of the protein.^202^ For citrate, dextran and PEG coated maghemite MIONPs opposed results were reported, where no changes in Fb conformation were observed.^203^ For citrate and dextran, Fb was adsorbed by displacing the coating, resulting in increased NP stability, while PEG coating prevented the adsorption of Fb.

The adsorption of bovine serum albumin (BSA) onto hematite MIONPs has been interpreted as a multistage process where the BSA undergoes conformational changes, including unfolding and refolding, that depends on the competition between protein–particle and protein–protein interactions.^204^ Interestingly, pre-adsorption of phosphates on hematite NPs has been observed to decrease the surface coverage of BSA and mitigate the extent of conformational alterations.^205^ Upon adsorption onto magnetite MIONPs, the secondary structure of BSA was altered suggesting partial protein unfolding and aggregation on NP surface driven by hydrophobic interactions.^206^ Additionally, it has been shown that it is possible to adsorb human serum albumin (HSA) onto SPIONs to form a layer of approximately 3 nm where HSA evidenced negligible conformation changes.^207^ The coated SPIONs showed improved colloidal stability and reduced dissolution. Furthermore, as reviewed by Chubarov,^208^ coating of MIONPs with albumins had been thoroughly investigated as it lowers the cytotoxicity and non-specific interaction of the NPs and improve their targeting capabilities and biocompatibility. Moreover, the interactions of biomolecules from other families, such as lipids, nucleic acids, and carbohydrates, with MIONPs have also been explored. The review by Abarca-Cabrera and colleagues documents several studies in this area.^209^

Numerous studies have investigated how the properties of MIONPs influence protein adsorption and the specific composition of the protein corona in complex biological media such as serum or plasma, with a particular focus on surface coating and related characteristics. For instance, in a study by Jansch *et al.*,^210^ the adsorption of proteins from human plasma onto SPIONs coated with citric acid and triethylene glycol was examined. Their investigations revealed that the most dominant adsorbed proteins were fibrinogen and immunoglobulins. Interestingly, they did not observe a typical Vroman effect in the adsorbed proteins over time. Another study delved into the protein corona formation on SPIONs coated with citric acid (CA), polyacrylic acid (PAA), and an oleic acid double layer (OAOA) when exposed to human plasma.^211^ It demonstrated similar adsorption patterns and kinetics for the former two coatings, while the latter exhibited a more slowly evolving adsorption pattern. Comparing the specific proteins adsorbed onto each type of NPs, it was observed that OAOA-coated NPs were enriched with lipid carrier proteins, such as Apolipoprotein A-1, while CA-coated NPs had higher levels of opsonins like immunoglobulins and complement factors. Portilla and co-workers found a comparable protein corona composition formed on positively coated MIONPs (aminopropylsilane, APS), neutrally coated (dextran), and negatively coated (dimercaptosuccinic acid, DMSA) when exposed to DMEM supplemented with FBS. However, variations in protein abundance were observed based on the coating, with a notable contribution from ion-binding proteins.^212^ The authors associated this finding with the presence of Fe ions on the IONP surface, suggesting their involvement in protein selection.

In a different study, Hirsch *et al.*^213^ investigated protein adsorption from FBS onto SPIONs coated with negative (–COOH), neutral (–OH), or positive (–NH_2_) polyvinyl alcohol (PVA) coatings. They found similar patterns of protein adsorption across all three NPs. Therefore, they suggested that protein adsorption was independent of surface (PVA) charge. Similarly, Sakulkhu and their co-workers^214^ explored positively, neutrally, and negatively PVA or dextran coated SPIONs and discovered that PVA-coated ones adsorbed more proteins from fetal bovine serum (FBS), with a preference for proteins with molecular weights between 70 and 100 kDa.

Stepien *et al.*^215^ investigated the protein corona formed on MIONPs coated with PMAO and further functionalized with glucose or polyethylene glycol (PEG; *M*_W_ = 5000 Da). They observed that the total protein adsorption followed the order of PMAO > PMAO–glucose > PMAO–PEG. Moreover, it was demonstrated that PMAO–glucose-coated MIONPs adsorbed more opsonins, such as complement factors and apolipoproteins, while PMAO-PEG-coated MIONPs exhibited higher levels of dysopsonins, including albumin. Another study reported that MIONPs coated with PEG (*M*_W_ = 2000) exhibited a similar corona composition compared to MIONPs coated with DMSA when exposed to human plasma.^216^ However, the former displayed a broader range of adsorbed proteins. Furthermore, MIONPs-PEG had lower levels of apolipoprotein A–I (opsonin) and higher levels of albumin (dysopsonin). Notably, 65–70% of the soft corona proteins were also detected in the hard corona.

Wiogo and co-workers^217^ investigated the influence of polymer charge, molecular weight, and branching on the adsorption of proteins from RPMI supplemented with FBS or BSA onto bare magnetite NPs or those coated with positively charged branched polyethyleneimine (b-PEI, 25 kDa and 0.8 kDa), linear PEI (LPEI, 25 kDa), or negatively charged polymethacrylic acid (PMAA, 20 kDa). They found that the quantity of adsorbed proteins follows the order bare b-PEI (25 kDa) NPs > bare NPs – bPEI (0.8 kDa) NPs > L-PEI NPs > PMAA. Moreover, they shown that PEI coated NPs tend to adsorb proteins with pI < 7 while bare and PMAA and bare ones with pI > 7. Other report evaluated the influence of particle surface hydrophobicity on the protein corona formation on SPIONs.^218^ To these ends, SPIONs coated with different materials ranging from lower to higher hydrophobicity as polyacrylic acid (PAA), an amphiphilic block copolymer (AMP), and azelaic acid (AZA) were incubated with albumin and IgG-depleted human serum. The research indicated that more hydrophobic NP surfaces tended to adsorb more hydrophobic proteins. Additionally, it was observed a correlation between NP surface hydrophobicity and the proportion of fast-exchange proteins in the corona (soft corona).

In parallel to protein corona studies, extensive research has been conducted to explore the colloidal stability of MIONPs in biological relevant media such as single protein solutions,^219–222^ cell culture media^217,223,224^ or media containing sera/plasma^211,221,225,226^ has been found that MIONPs aggregation is highly dependent of NP properties (*e.g.*, surface coating), as well as media composition, and is profoundly influenced by protein adsorption. Many researchers have reported that protein corona formation improves MIONPs colloidal stability.^211,217,219,223,227–230^ For instance, Wells and co-workers^228^ showed that FCS reduced the aggregation of MIONPs in water, PBS and endothelial cell basal medium-2. Safi *et al.*^223^ observed that citrate coated MIONPs aggregate in cell culture media RPMI, but this trend is reduced in presence of 10% FBS when corona formation took place. Moreover, multiple studies concerning MIONPs of different compositions (*e.g.*, maghemite, magnetite) with various surface coatings (such as citrate, PAA, PMAA, PEI, dextran, heparin), dispersed in a range of media (*e.g.*, PBS, DMEM), and exposed to diverse protein sources (*e.g.*, FBS, human serum/plasma) have consistently reported that serum or plasma lead to enhanced colloidal stability of the NPs, and this stability progressively improves with increasing protein concentrations.^211,217,219,227–230^ On the contrary, a smaller number of studies have reported that protein adsorption does not prevent the aggregation of MIONPs.^213,222,231^ As an example, Etheridge and colleagues^231^ observed that commercial MIONPs aggregate in water across varying FBS concentrations and examined the properties of these aggregates. Table 2 compiles selected references related to protein adsorption, corona formation, and MIONPS aggregation in biologically relevant fluids. It highlights the key aspects of NP properties (*i.e.*, synthesis methods, size, coating) and the media (*e.g.*, PBS supplemented with FBS, human plasma, *etc.*) used in the studies, along with the experimental techniques employed (*e.g.*, TEM, DLS, SDS-PAGE, *etc.*).

**Table 2:** Selected references addressing protein corona formation and aggregation of MIONPs in biological fluids

Focus of the study	NP size	Surface coating	NP synthesis	References
MIONPs colloidal stability in water, PBS and cell culture media supplemented with FBS [DLS]	4–8 nm [TEM]	Poly(TMSMA-r-PEGMA)	Coprecipitation	Lee *et al.*^232^
	12–16 nm [DLS]			
MIONPs colloidal stability in water and DMEM supplemented with FBS [TEM, DLS, *ζ*-potential]	PEI coated NP ∼25 nm (octahedra) [TEM]	PEI and PAA	Modified oxidative hydrolysis	Calatayud *et al.*^233^
Protein adsorption onto MIONPs [TGA, *ζ*-potential]	PAA coated NP ∼32 nm (sphere). [TEM]			
	NP forms stable agglomerates in suspension			
MIONPs colloidal stability in gastric and intestine simulated fluids and at different pH values [DLS]	All NPs *ca.* 10 nm [TEM]	PMAO) and/or casein	Thermal decomposition	Huang *et al.*^234^
	In aqueous suspension, 10–24 nm depending on coating [DLS]	Loaded with doxorubicin or indocyanine green		
Influence of coating on MIONPs colloidal stability in HEPES saline [DLS, *ζ*-potential]	Bare NP: ∼7–8 nm [TEM]	Various linear or dendritic PEG based oligo/polymers	Thermal decomposition	Gillich *et al.*^235^
Influence of coating on temperature inducing MIONPs clouding point at different salt concentrations [DLS, *ζ*-potential]	Coated NP in aqueous suspension: from 10–100 nm depending on coating [DLS]			
Colloidal stability of and protein adsorption onto MIONPs in PBS and 10% FBS [DLS, HPLC]	∼8 nm [TEM]	Short zwitterionic dopamine sulfonate	Thermal decomposition	Wei *et al.*^236^
	∼10 nm [DLS]			
Influence of coating on the colloidal stability of MIONPs when incubated in aqueous media at different pH and salt concentration [visual observation]	∼22 nm [TEM]	Various cathecol based PEG oligomers	Thermal decomposition	Na *et al.*^237^
	35–40 nm [DLS]			
MIONPs colloidal stability in DMEM and RPMI supplemented or not with FCS [DLS, turbidity]	Bare NP: ∼10 nm [TEM]	PVA and PEI	Coprecipitation	Petri-Fink *et al.*^238^
	PEI coated: NPs embedded in polymer matrix			
Protein adsorption upon exposure of MIONPs to FBS [SDS-PAGE]	Bare NP; ∼5 nm [TEM]	Plain, amino- and carboxyl-dextrans	Coprecipitation	Amiri *et al.*^239^
Protein (hard) corona thickness upon exposure of MIONPs to FBS [DCS]	Dextran coated NP: ∼15 nm [TEM]			
Colloidal stability of MIONPs in PBS, DMEM and DMEM supplemented with FBS [DLS]	∼12 nm [TEM]	SAS and SAS + PEG	Thermal decomposition	Fang *et al.*^240^
Influence of coating on protein adsorption upon exposure of MIONPs to FBS (10 or 55%) in PBS. [*ζ*-potential, BCA assay]	Bare NP ∼8 nm [TEM]	PG plain and containing amine, carboxyl and sulfate groups	Thermal decomposition	Zou *et al.*^241^
Influence of coating on protein corona composition upon exposure of MIONPs in the same media. [SDS-PAGE, LC-MS/MS]				
Impact of protein adsorption on MIONPs colloidal stability the same media				
Colloidal stability of MIONPs under DMEM + FBS [DLS, *ζ*-potential]	Bare NP ∼7.3 nm [TEM]	Bare	Coprecipitation	Casals *et al.*^242^
Influence of coating on colloidal stability of MIONPs under RPMI, and FBS [TEM, DLS]	Bare and coated NP ∼10–20 nm [TEM]	Linear PMAA, linear PEI, branched PEI and oligo PEI	Coprecipitation	Wiogo *et al.*^217^
Influence of coating on protein corona composition upon exposure of MIONPs to FBS [1D-SDS-PAGE, LC-MS/MS]				
Protein corona size upon exposure of MIONPs to BSA [1D-SDS-PAGE, DLS, TEM, FTIR, EDS]	Bare NP ∼495 nm [SEM]	Uncoated	Polyol method	Yang *et al.*^206^
	Bared NP ∼597 nm [DLS]			
	BSA adsorbed NP ∼797 nm [DLS]			
Influence of coating on colloidal stability of MIONPs under pH and NaCl [DLS, *ζ*-potential]	PEG with different size coated NP 120–170 nm [DLS]	PEG of different *M*_w_	Coprecipitation	Illés *et al.*^243^
Influence of coating on colloidal stability of MIONPs under water, PBS, citrate, DMEM, and FBS [DLS]	Bare NP ∼8.3 nm [TEM]	Citrate and PAA	Coprecipitation	Safi *et al.*^223^
	Bare NP ∼24 nm [DLS]			
	Citrate coated NP, idem for uncoated			
	PAA2K coated NP ∼30.0 nm [DLS]			
Influence of coating on colloidal stability of MIONPs under FBS [UV-visible at 480 and 566 nm]	Bare NP ∼11.9 nm [DLS]	PVA with different charges	Coprecipitation	Hirsch *et al.*^244^
	PVA (COOH, OH, and NH_2_) coated NP ∼34.9 nm [DLS]			
Influence of coating on colloidal stability of MIONPs under NaCl, and NH_4_Cl, DMEM, and FCS [DLS]	Citrate coated NP ∼23 nm [DLS]	PPEG, PAA (2 kDa and PAA (5 kDa)	Coprecipitation	Chanteau *et al.*^245^
	PPEG coated NP ∼17 nm [DLS]			
	PAA2K coated NP ∼19 nm [DLS]			
	PAA5K coated NP ∼22 nm [DLS]			
Influence of coating on colloidal stability of MIONPs under water, PBS, and FBS [DLS]	Bare NP ∼6.5 nm [TEM]	PEO-DOPA, PEO-nitroDOPA and PEO-tri-nitroDOPA	Thermal decomposition	Saville *et al.*^246^
	PEO-DOPA coated NP ∼39.7 nm [DLS]			
	PEO-nitroDOPA coated NP ∼89.6 nm [DLS]			
	PEO-tri-nitroDOPA coated NP ∼68.7 nm [DLS]			
Influence of coating on colloidal stability of MIONPs under NaCl, temperature, and human blood plasma [DLS]	PLA-PEG coated NP ∼100 nm [DLS]	Nanospheres of PLA-PEG	Thermal decomposition	Bakandritsos *et al.*^247^
Influence of coating on protein corona composition upon exposure of MIONPs to human blood plasma [2D-PAGE, ExPASy/SWISS-2D-PAGE]	Citrate/TREG-stabilized NP ∼7.8 nm [TEM]	Citrate-TREG	Polyol method	Jansch *et al.*^210^
	Citrate/TREG-stabilized ∼12.2 nm [DLS]			
Influence of coating on colloidal stability of MIONPs under PBS, buffer HEPES, and buffer Tris[DLS, DCS, *ζ*-potential, ATR-FTIR]	Bare NP ∼10 nm [TEM]	OA, citrate and PAA	Coprecipitation	Jedlovszky-Hajdú *et al.*^211^
	OA coated NP ∼25–150 nm [DLS]			
Protein corona thickness of MIONPs under PBS + FBS [DLS]	PAA coated NP ∼25–150 nm [DLS]			
Influence of coating on protein corona composition under incubation of MIONPs with PBS + FBS [1D-SDS-PAGE, LC-MS/MS]	Citrate-coated NP ∼25–150 nm [DLS]			
Influence of coating on protein corona composition under *in vivo* interaction of MIONPs with rat serum [LC-MS/MS]	PVA coated NP (neutral) ∼90 nm [DLS]	PVA	Coprecipitation	Sakulkhu *et al.*^248^
	PVA coated NP (positive) ∼95 nm [DLS]			
	PVA coated NP (negative) ∼91 nm [DLS]			
Influence of NP size on protein adsorption from human blood serum [1D-SDS-PAGE]	Bare NP ∼30, 225 and 375 nm [TEM]		Polyol method	Hu *et al.*^249^
Influence of NP size on protein corona composition under incubation of MIONPs with human blood serum [RPLC-MS/MS]				
Influence of coating and NP size on protein corona formation under FBS [DLS, *ζ*-potential]	Core size NP of 3.5, 6, 9, 13 and 15 nm [TEM]	Plain, COOH and NH_2_ dextrans	Thermal decomposition	Mahmoudi *et al.*^250^
Influence of coating and NP size on protein corona composition under incubation of MIONPs with FBS [1D-SDS-PAGE]	COOH-dextran coated NP ∼9–30 nm [DLS]			
	Plain dextran coated NP ∼9–30 nm [DLS]			
	NH_2_-dextran coated NP ∼9–30 nm [DLS]			
Influence of coating on colloidal stability of MIONPs under RPMI, DMEM, FBS mouse blood serum, and human blood serum [DLS, *ζ*-potential]	DMSA coated NP ∼64.7 nm [DLS]	DMSA, dextran and APS	Coprecipitation	Portilla *et al.*^251^
	Dextran coated NP ∼116.4 nm [DLS]			
Influence of coating on protein corona composition under incubation of MIONPs with serum samples of bovine, human, and mouse [nano-LC-ESI-MS/MS]	APS coated NP ∼112.3 nm [DLS]			
Influence of coating on protein corona composition under incubation of MIONPs with mouse blood plasma [LC-MS, LC-MS/MS]	NH_2_ dextran coated NP ∼50 nm [purchased]	NH_2_–dextran	Purchased from Micromod	Simberg *et al.*^252^
	Nanoworms with no crosslinking			
Influence of coating on colloidal stability of MIONPs under KCl, PBS, and FBS [DLS, *ζ*-potential]	PVA-NH_2_ coated NP ∼37.9 nm [DLS]	PVA and dextran with different charges (NH_2_, COOH and OH)	Coprecipitation	Wiogo *et al.*^217^
	PVA-OH coated NP ∼28.3 nm [DLS]			
Influence of coating on protein corona composition under incubation of MIONPs with FBS [LC-MS/MS]	PVA-COOH coated NP ∼38.1 nm [DLS]			
	Dextran-NH_2_ coated NP ∼18.8 nm [DLS]			
	Dextran-OH coated NP ∼19.7 nm [DLS]			
	Dextran-COOH coated NP ∼18.3 nm [DLS]			
Influence of coating on transferrin adsorption [SEC-FPLC with 125I-protein]	Iron oxide core ∼11 nm [electron micrograph]	PEG amines	Thermal decomposition	Bargheer *et al.*^253^
	PEGamine coated NP ∼25 nm [DLS]			
Influence of protein corona on NP [DLS, *ζ*-potential]	Dextran coated NP (Nanomag-D-spio) ∼81.8 nm [DLS]	Dextran	Purchased from Micromod	Vogt *et al.*^254^
Influence of coating on protein corona composition under incubation of MIONPs with human blood plasma [LC-MS/MS]				
Influence of protein corona on IONP size [DLS]	Phosphorylated mPEG coated NP ∼13.1 nm [TEM]	Methoxy-PEG	Thermal decomposition	Zhang *et al.*^255^
Influence of coating on protein corona composition under incubation of MIONPs with mice blood plasma [LC-MS/MS]				
Influence of coating on colloidal stability of MIONPs under water, PBS, DMEM, and FBS [DLS, *ζ*-potential]	Bared NP ∼12.7 nm [TEM]	APS, dextran and DMSA	Coprecipitation	Portilla *et al.*^212^
	APS coated NP ∼122.4 nm [DLS]			
Influence of coating on protein adsorption [BCA assay]	Dextran coated NP 109 nm [DLS]			
Influence of coating on protein composition under incubation of MIONPs with FBS [LC-ESI-MS/MS]	DMSA coated NP ∼82.8 nm [DLS]			
Influence of coating on colloidal stability of MIONPs under RPMI + FCS [*ζ*-potential]	PEI coated NP ∼150 and 182 nm [DLS]	Branched PEI	Purchased from Chemicell	Gräfe *et al.*^256^
Influence of coating on protein corona composition under incubation of MIONPs with FCS [1D-SDS-PAGE]				
Influence of coating on protein corona composition under incubation of MIONPs with mice serum blood plasma [LC-MS/MS]	Bare NP ∼12 nm [TEM]	PMAO, glucose and PEG	Thermal decomposition	Stepien *et al.*^215^
	PMAO coated NP ∼21.9 nm [TEM]			
Influence of coating on colloidal stability of MIONPs under water, PBS, and DMEM	Glucose coated NP ∼22.3 nm [TEM]			
Influence of coating on NP properties [DLS, *ζ*-potential]	PEG coated NP ∼36.2 nm [TEM]			

### 2.2. Accumulation in cells and organs

The accumulation of magnetic iron oxide-based NPs in cells involves complex processes influenced by various factors, including NP properties and cellular mechanisms. One approach for NP accumulation in cells involves directly attaching NPs to the cell surface, facilitating their interaction with cellular components. This direct binding can lead to the internalization of NPs into the cytosol through receptor-mediated endocytosis, fluid-phase endocytosis, or phagocytosis. By attaching to the cell surface, MIONPs can initiate cellular uptake processes that contribute to their accumulation within cells.

Cellular uptake of these NPs can be enhanced by using several strategies, such as applying magnetic fields and introducing ligands onto the NP surface. The application of external magnetic fields can enhance the cellular uptake of MIONPs, increasing cell particle accumulation.^257^ Pulsed magnetic fields have been shown to promote cellular transport and NP accumulation. By applying magnetic forces, researchers can manipulate the movement and localization of NPs, guiding their internalization and accumulation within target cells.^258^ This approach offers a controlled and efficient method for promoting the accumulation of NPs in specific cell populations, enabling targeted delivery and enhanced therapeutic outcomes. Min *et al.*^259^ demonstrated that a pulsed magnetic field could improve the transport of MIONPs through cell barriers by minimizing cell surface aggregate formation and maximizing the force driving cellular uptake and transport of particles.

In contrast, constant magnetic fields can inhibit cellular uptake by inducing the formation of aggregates that exceed the size of endocytic vesicles.^257^ Using magnetic fields as a driving force for NP accumulation offers a controlled and efficient method for enhancing cellular uptake and intracellular accumulation of magnetic NPs. Moreover, specific cellular processes such as endocytosis and phagocytosis can facilitate the internalization of MIONPs into cells.^260^ These mechanisms involve the formation of vesicles that engulf NPs, allowing for their internalization and accumulation within cells.^260^ Complementary techniques, such as correlative microscopy, provide insights into the spatial distribution of NPs within cells, revealing co-accumulation with endogenous cellular components like Fe in macrophages.^260^

**Fig. 7:**
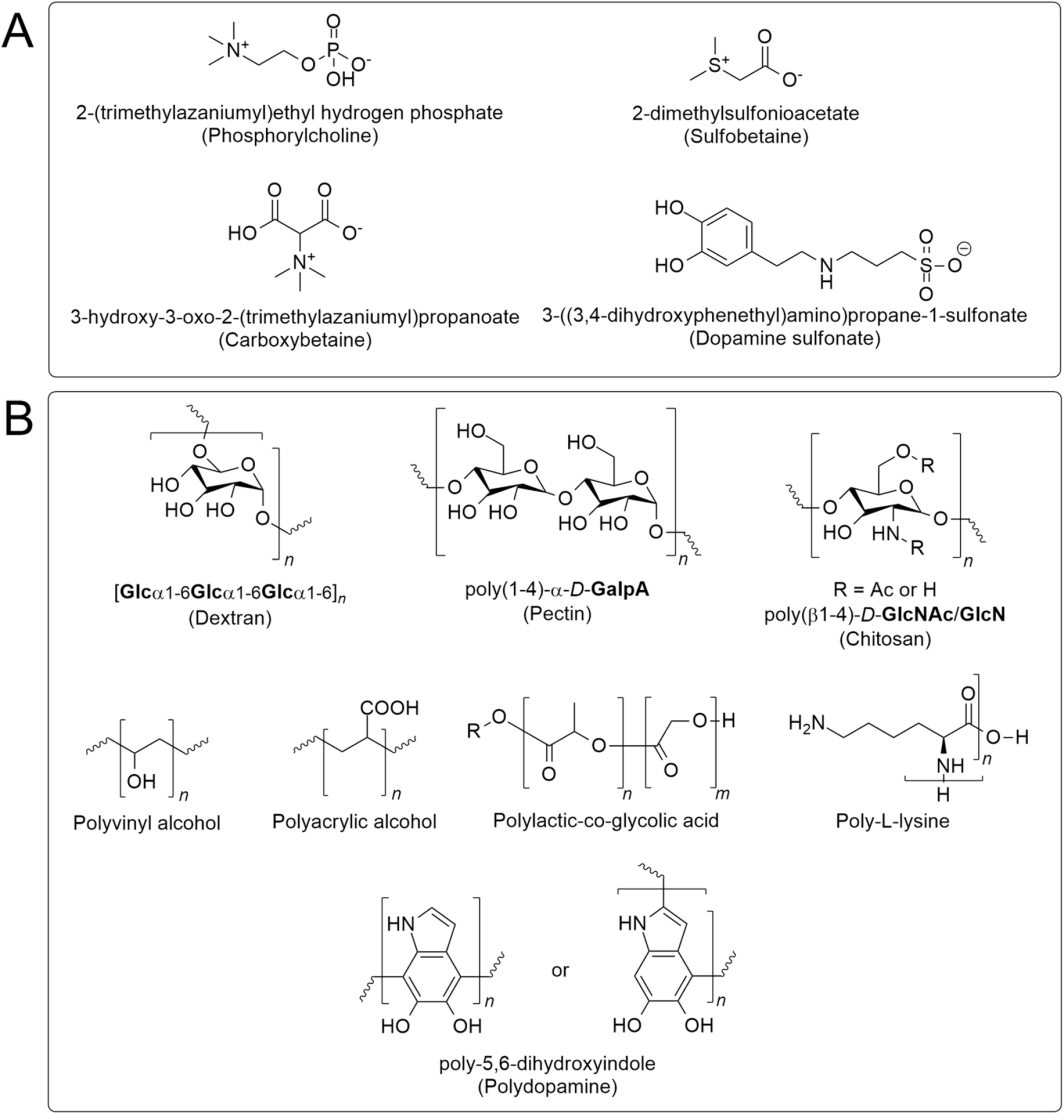
Structures of commonly used ligands of MIONPs for enhanced cellular uptake. (A) Zwitterionic and (B) polymeric ligands. Polyvinyl alcohol (PVA) is a neutral, hydrophilic polymer with no inherent charge at physiological pH, making it non-ionic. Conversely, polyacrylic acid (PAA) may carry a negative charge when carboxyl groups are ionized under physiological conditions. Polylactic-*co*-glycolic acid (PLGA) is generally neutral, with a slightly hydrophobic character due to its ester linkages, but it may not carry a significant charge. In contrast, poly-l-lysine (PLL) may be positively charged, as its amine groups are protonated at physiological pH, resulting in a cationic character that strongly influences its interaction with negatively charged biological molecules.

Understanding the intracellular trafficking and accumulation of NPs is crucial for optimizing their delivery and targeting in biomedical applications. In addition to external magnetic fields, specific ligands or targeting moieties (Fig. 7 and Table 3) on the surface of MIONPs can enhance their accumulation in target cells.^261^ Functionalizing NPs with ligands that bind to cell-specific receptors can promote their selective uptake and accumulation in desired cell populations.^261^ This targeted approach allows for the precise delivery and accumulation of NPs in specific cell types, improving their efficacy in various biomedical applications. Polyethylene glycol (PEG) is commonly used to improve biocompatibility and stability, creating a hydrophilic shell that reduces protein adsorption and rapid clearance by the immune system. However, it often decreases non-specific cellular uptake. To achieve specific uptake, targeting ligands such as antibodies, peptides, and aptamers are employed, which bind to overexpressed receptors on target cells, facilitating receptor-mediated endocytosis. Cell-penetrating peptides (CPPs), like *trans*-activator of transcription (TAT)-derived peptides (derived from the human immunodeficiency virus), significantly enhance cellular uptake through direct translocation or endocytosis by interacting with the cell membrane.^262,263^ Small molecules, including folic acid, glucose, and hyaluronic acid, can target specific receptors on cells, promoting selective uptake.^264–266^ Zwitterionic polymers as ligands offer a neutral overall charge that reduces nonspecific interactions while balancing stealth properties and cellular interaction.^267–270^ Carbohydrate coatings like monosaccharides or disaccharides (*e.g.*, Glu, Gal, Suc, and Mal) and polymers (*e.g.*, dextran, pectin, and chitosan) stabilize MIONs, reduce opsonization, and improve circulation times by decreasing uptake by non-target cells.^271–274^ The optimal ligand choice depends on the balance between stealth and targeting, the target cell type, and the intended biomedical application, allowing for precise control over how MIONs interact with biological systems.

**Table 3:** Effects of surface modification on MIONPs, highlighted in recent studies

Aspect	Effect of surface modification	Examples of modifications	References
Colloidal stability	Enhances colloidal stability by preventing agglomeration due to electrostatic or steric hindrance	PEGylation, citrate coating, silica coating	Lahooti *et al.*,^275^ de Sousa *et al.*^35,276^
Chemical stability	Prevents chemical reactions of the inorganic core	Oleate moieties	Orozco-Henao *et al.*^277^
Biocompatibility	Improves biocompatibility, reducing cytotoxicity and making MIONPs suitable for biomedical applications	Dextran, chitosan, or PEG coatings	Lahooti *et al.*,^275^ Phalake *et al.*,^278^ Gholipour *et al.*,^279^ Moghadam *et al.*^280^
Targeting specificity	Functionalization with targeting ligands enables selective interaction with specific cells, tissues, or biomolecules	Antibodies, peptides, or aptamer functionalization	Phalake *et al.*,^278^ Rahman & Ochiai,^281^ Bilbao-Asensio *et al.*^282^
Surface charge	Alters surface charge, affecting interaction with cells and biodistribution	Zwitterionic coatings, carboxyl, or amine groups	Rahman & Ochiai^281^
Magnetic properties	May affect the magnetic moment and relaxation behavior due to surface spin canting or interaction with coating material	Thin polymer layers or heavy ligand adsorption	Lahooti *et al.*,^275^ Salafranca *et al.*,^283^ Vasilakaki *et al.*^284^
Drug loading efficiency	Facilitates drug conjugation or encapsulation, enabling drug delivery applications	Functional groups like –COOH, –NH_2_, or thiol groups	Gendelman *et al.*,^2^ Dias *et al.*^6^
Imaging capabilities	Enhances imaging capabilities by attaching fluorescent dyes or radioactive labels for multimodal imaging (MRI, fluorescence, *etc.*)	Fluorophores or radionuclide labels	Gupta *et al.*,^285^ Rivas-Aiello *et al.*^286^
Hydrophilicity	Increases water dispersibility, making MIONPs more suitable for biological environments	Hydrophilic polymers like PVP or PEG	Lahooti *et al.*^275^
Reduced toxicity	Lowers oxidative stress or iron leaching that can occur in physiological environments	Silica or gold shells, biopolymer coatings, naturally occurring antioxidants (*e.g.*, phenolic acids)	Moghadam *et al.*^280^
Biodistribution	Alters circulation time and accumulation in specific organs (*e.g.*, liver, spleen, or tumor tissue), depending on the functionalization	PEGylation for stealth properties, folic acid targeting	Lahooti *et al.*,^275^ Ramezani *et al.*,^287^ Gupta *et al.*^285^
Biomolecule specificity	The presence of oxygenized groups (*e.g.*, carboxyl) provides affinity with single-stranded nucleic acids ex vivo	Graphene oxide	Rivera *et al.*^288^

The structure of zwitterionic ligands (*e.g.*, sulfobetaine, phosphorylcholine, carboxybetaine) on MIONPs plays a crucial role in modulating cellular uptake by altering surface properties.^267–270^ These ligands balance positive and negative charges, creating a neutral surface that reduces nonspecific interactions, immune recognition, and cellular uptake, making them ideal for targeted delivery. Their hydrophilic nature forms a dense hydration layer, enhancing “stealth” properties and prolonging circulation time. However, this can also decrease overall uptake unless specific targeting mechanisms are employed. Polysaccharides like dextran, chitosan, and pectin also influence MIONP uptake by modifying surface interactions (Fig. 7B).^271–274^ Dextran, a neutral, hydrophilic polymer, stabilizes MIONPs and reduces uptake. In contrast, chitosan's positive charge increases uptake through electrostatic interactions, making it suitable for drug delivery. Pectin, negatively charged, tends to reduce uptake but is useful for controlled-release applications. Similarly, polymers like PVA, PAA, PLGA, PLL, and polydopamine affect MIONP cellular uptake (Fig. 7B). PVA provides stability and reduces nonspecific uptake, while PAA, with its negative charge, stabilizes MIONPs but may also reduce uptake unless functionalized. PLGA enables controlled release and enhances uptake *via* endocytosis. With its positive charge, PLL promotes strong cellular interactions and uptake, which is ideal for gene delivery, while polydopamine offers versatile functionalization and tunable uptake based on its bioinspired structure. Polydopamine, inspired by the adhesive properties of mussel proteins, significantly influences the cellular uptake of MIONPs through its unique structure and surface chemistry.^289^ The catechol and amine groups in polydopamine allow it to strongly adhere to various surfaces, forming a versatile and stable coating on MIONPs. This bioinspired coating enhances the biocompatibility of the NPs and provides a platform for further functionalization, such as attaching targeting ligands or therapeutic agents, which can enhance specific cellular uptake. The hydrophilic nature of polydopamine and its ability to create a dense surface layer can reduce nonspecific interactions with cell membranes, potentially leading to lower non-specific cellular uptake. However, by modifying the polydopamine surface by targeting moieties or adjusting the polymer density, the uptake of MIONPs by specific cells can be significantly increased, making polydopamine-coated MIONPs highly adaptable for various biomedical applications, including targeted drug delivery and imaging.^289^

**Fig. 8:**
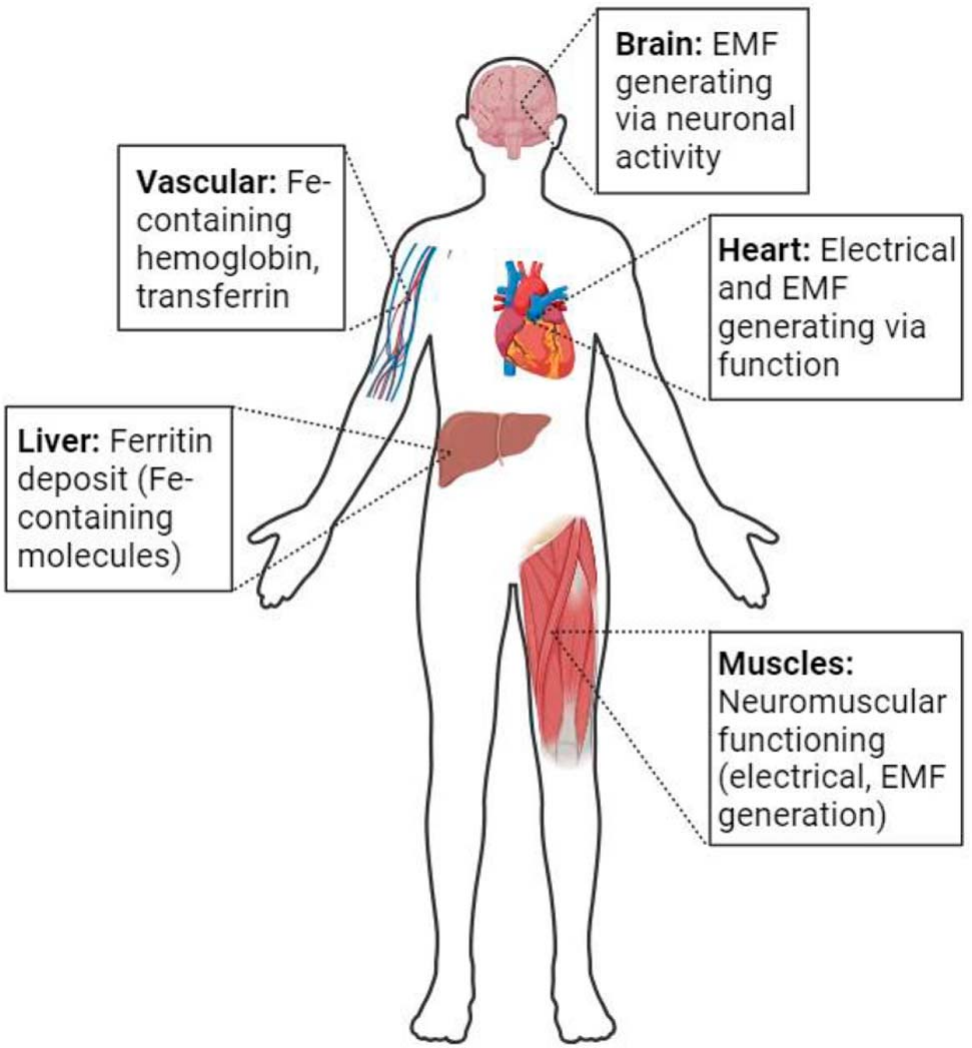
Human organs and organ systems where magnetic particles and electrical and electromagnetic fields (EMF) are naturally found. Reproduced from ref. 292 with under a Creative Commons (CC BY 3.0) License from MDPI, Basel Switzerland, 2024.

Various factors can influence the accumulation of MIONPs in organs. These NPs' biodistribution and organ distribution are intricate and can be impacted by NP size, surface charge, magnetic property, and coating.^290^ The interplay between NP size and charge is pivotal in determining the distribution of systemically delivered MIONPs within the body.^290^ The surface functionalization of MIONPs can affect cell internalization, potentially influencing their accumulation in specific organs. These NPs can undergo biotransformation processes within the body, leading to their accumulation in tissues like the spleen, liver, and lungs over extended periods.^291^ The transformation of superparamagnetic MIONPs into other forms within organs underscores the dynamic nature of these NPs in biological environments.^291^ Additionally, the magnetic properties of these NPs, such as superparamagnetism, can impact their behavior in biological systems and their interaction with cells and tissues. The unique magnetism of MIONPs can result in their accumulation in specific organs, including the brain, where magnetic particles and electrical and electromagnetic fields (Fig. 8) have been naturally found.^292,293^ This is attributed to magnetic NPs, such as MIONPs, which can be confined within endosomes and undergo processes like degradation, storage, and neocrystallization.^293^

Magnetic iron oxide-based NPs can accumulate in cells through various mechanisms, including endocytosis, magnetic targeting, and interactions with specific cellular components. Zhang *et al.*^261^ discussed the force-mediated endocytosis of MIONPs for magnetic targeting of stem cells, highlighting that the cellular uptake of MIONPs occurs through an endocytic pathway, with the NPs being localized in lysosomes. The intracellular MIONPs had no adverse effects on human muscle-derived stem cells (hMDSCs) and their subsequent passaging. It was also shown that MIONPs were localized to target cells and not translocated to other cells in an *in vitro* coculture system. This emphasizes how magnetic forces can facilitate the internalization of NPs into cells, leading to their accumulation in specific cellular compartments.

**Fig. 9:**
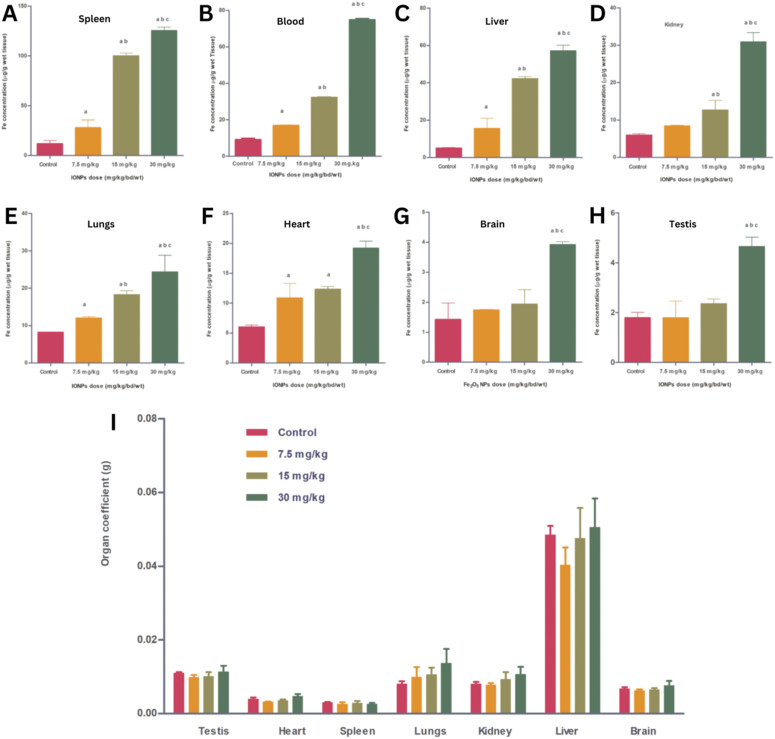
Biodistribution of intravenously administered MIONPs in male Wistar rats. Bioaccumulation patterns of MIONPs in different organs of Wistar rat (A–H): spleen (A), blood (B), liver (C), kidney (D), lungs (E), heart (F), brain (G), and testis (H) treated with varying doses of MIONPs. All the organs studied showed dose-dependent accumulation of MIONPs that was statistically significant (*p* < 0.05), except for the brain and testis, where substantial distribution was only seen in the high-dose (30 mg kg^−1^) group. The following letters indicate significant inter-group differences (*p* < 0.05): a (against control), b (*vs.* 7.5 mg kg^−1^ MIONPs), and c (*vs.* 15 mg kg^−1^ MIONPs). Organ coefficients—organ weight (g) divided by the animal body weight (g)—of Wistar rats given varying IONP injection dosages in comparison to the control group (I). The groups that received NP injections and the control group did not differ significantly (*p* < 0.05). Results are presented as mean ± standard deviation (*n* = 6) with standard deviation error bars. Tukey's test was used to do multiple comparisons and one-way analysis of variance to establish statistical significance. Reproduced from ref. 294 with under a Creative Commons (CC BY 3.0) License from Dove Medical Press Limited, 2019.

Several studies have investigated the biodistribution, clearance, and morphological alterations of MIONPs in different animal models. These investigations have provided insights into the accumulation patterns of MIONPs in organs such as the liver, spleen, lungs, and brain, shedding light on their potential impact on these tissues. For example, Gaharwar *et al.*^294^ conducted an *in vivo* study to investigate the accumulation and excretion of Fe_2_O_3_ NPs (average size of ∼30–35 nm) in male Wistar rats *via* intravenous administration in the caudal vein (Fig. 9).

The level of organ Fe concentration accumulation was found to be spleen > blood > liver > kidney > lung > heart > testis > brain. The urine excretion profile shows its maximum concentration the day after administration was maintained until the 28^th^ day. In contrast, the Fe content in feces remained high during the first three days after injection. The paper did not mention the rationale as to why the type of NPs administered were accumulated on these specific organs; it can be explained by how numerous studies have demonstrated that the NPs with hydrodynamic sizes (*d*_H_) in the range of 15–100 nm have the most extended bloodstream circulation times and, as a result, are more likely to reach other organs and targets, including the brain, artery walls, and—in pathological conditions—lymph nodes and tumors.^295–299^

**Fig. 10:**
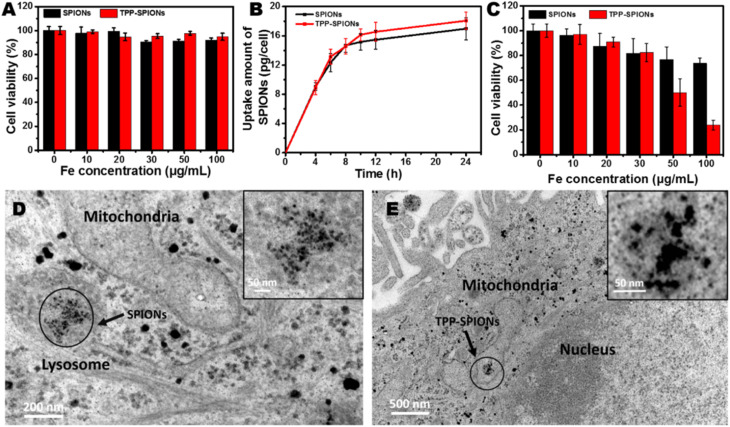
Comparative analysis of SPIONs and TPP-SPIONs in HepG2 Cells. (A) Assessment of cell viability in HepG2 cells coincubated with SPIONs and TPP-SPIONs using the MTS colorimetric assay. (B) Quantification of time-dependent uptake of SPIONs and TPP-SPIONs. (C) Analysis of dose-dependent cell viability in HepG2 cells subjected to magnetic hyperthermia with SPIONs and TPP-SPIONs. Bio-TEM images of HepG2 cells after incubation with 50 μg mL^−1^ of SPIONs and TPP-SPIONs for 12 h. Arrows in (D) indicate NP accumulation in the endo/lysosomes, while arrows in (E) highlight the localization of mito-targeted NPs within the mitochondria. Reproduced from ref. 300 with permission from American Chemical Society, copyright 2019.

MIONPs have also been shown to accumulate in various organelles within healthy cells, influencing cellular processes and responses. Studies have provided insights into the intracellular localization of MIONPs, shedding light on their interactions with different organelles.^260,300–302^ One of the primary organelles where MIONPs have been found to accumulate in the mitochondria. TPP-SPIONs (triphenylphosphonium cation-superparamagnetic iron oxide nanoparticles) were evaluated using spheroids and monolayer-cultured HepG2 cells.^300^ The treatment of magnetic hyperthermia, large-scale spheroid exhibits less sensitivity to TPP-SPIONs and more *in vivo* tumor characteristics than monolayer cells (Fig. 10). This demonstrates that MIONPs can localize to the mitochondria, impacting mitochondrial function and cellular processes. This accumulation in mitochondria has implications for cellular metabolism, energy production, and even targeted therapies like mitochondrial hyperthermia treatment.^300^

Lysosomes have been identified as critical organelles where MIONPs accumulate and undergo degradation processes. Studies have highlighted the role of lysosomes in the cellular uptake and processing of MIONPs, indicating that these NPs settle into lysosomes, where they undergo metabolization and potentially release free Fe ions into the cell.^276^ The lysosomal pathway plays a crucial role in the fate of MIONPs within cells, influencing their biodegradation and potential toxicity. The cytoplasm of cells is also where MIONPs have been observed to accumulate, at least on microbiological specimens like *E. coli*, impacting cellular structures and functions. Transmission electron microscopy studies have shown that MIONPs can be internalized into the cytoplasm, affecting cellular components and potentially leading to cellular damage.^303^ The presence of MIONPs in the cytoplasm highlights their ability to interact with cellular machinery and influence cellular processes.^303^ Furthermore, MIONPs can be present in other organelles, such as the endoplasmic reticulum and nucleus, affecting cellular functions and responses. The accumulation of MIONPs in these organelles can affect cellular signaling, gene expression, and overall cell health.^302^ Understanding the intracellular distribution of MIONPs is crucial for elucidating their mechanisms of action and potential impact on cellular physiology. MIONPs have been shown to accumulate in various organelles within healthy cells, including mitochondria, lysosomes, cytoplasm, endoplasmic reticulum, and nucleus. The localization of MIONPs in these organelles influences cellular processes, metabolism, and responses, highlighting the importance of studying their intracellular fate for therapeutic and safety considerations.

The fate of MIONPs in cells is mostly studied in *in vitro* culture systems. An extensive review of Chen & Hou^304^ discussed the effect of MIONPs on cells and extracellular vesicles. MIONPs are absorbed by cells *in vitro* through endocytosis, with smaller MIONPs taken up mainly *via* clathrin- and caveolae-mediated endocytosis. At the same time, larger particles and aggregates are ingested through macropinocytosis and phagocytosis. Once internalized, MIONPs are transported to lysosomes, where they are degraded, releasing free Fe stored as ferritin or hemosiderin for further cellular use.^304^ The biocompatibility of MIONPs is mainly due to efficient Fe metabolism, allowing for Fe circulation and excess Fe exocytosis. However, cytotoxicity can arise from excessive reactive oxygen species (ROS) production, especially when MIONPs are present in concentrations above 50 μg mL^−1^.^304,305^ Proper control of IONP parameters can mitigate adverse effects on cell viability, metabolism, oxidative stress, and proliferation. In mesenchymal stem cells (MSCs), SPIONs can enhance cell migration and homing, particularly by upregulating CXCR4 expression, which improves the effectiveness of MSCs in tissue regeneration.^304^ In cancer cells, MIONPs can induce ferroptosis through the Fenton reaction, which generates highly toxic hydroxyl radicals in the acidic tumor microenvironment. This process is amplified by the co-delivery of H_2_O_2_ producers and metal catalysts, enhancing the therapeutic potential of MIONPs in cancer treatment.^304^

There are still limited studies on the fate of MIONPs in the cells and organs *in vivo*, more so in the speciation. Current studies and reviews such as that of Nowak-Jary & Machnicka^79,295^ are limited to discussing the localization and concentration (*e.g.*, general Fe content) of MIONPs *in vitro* after administration. However, it is generally accepted that once MIONPs are internalized by cells, their speciation can change depending on the intracellular environment.^304^ Typically, MIONPs are taken up through endocytosis, where they become enclosed in endosomes that mature into lysosomes with an increasingly acidic environment.^304^ In these acidic conditions, MIONPs may dissolve partially, releasing iron ions (Fe^2+^ or Fe^3+^) into the cellular environment. These ions can then be incorporated into the Fe metabolism of the cell, such as being stored in ferritin or used to synthesize heme and iron–sulfur clusters.^304^ However, some MIONPs, particularly those stabilized with strong surface coatings like dextran, may remain intact as metal oxides sequestered within lysosomes.^306^ The surface chemistry of the NPs plays a critical role in determining their stability and whether they remain as metal oxides or dissolve. Enzymatic activity in lysosomes may also contribute to the breakdown of MIONPs over time, releasing Fe that can reoxidize or precipitate into other forms within the cell. Thus, the fate of MIONPs after cellular uptake is influenced by the specific intracellular conditions and the surface characteristics of the NPs, leading to various potential outcomes ranging from retention as metal oxides to transformation into bioavailable Fe ions.

## 3. Biotransformation quantification and the fate of magnetic iron oxide

**Fig. 11:**
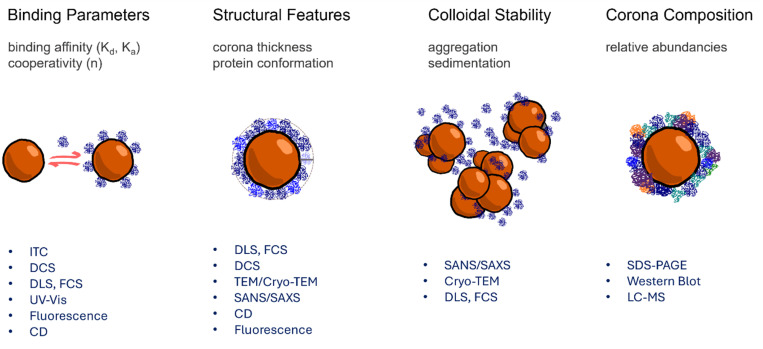
Various aspects of studying protein corona formation and colloidal stability of NPs in biological media, along with the commonly used techniques for each case. The proposed grouping of binding parameters, structural features, colloidal stability, and corona composition is logical, but it should be noted that other articles may present different categorizations. Acronyms: CD, circular dichroism; DCS, differential centrifugal sedimentation; DLS, dynamic light scattering; FCS, fluorescence correlation spectroscopy; ITC, isothermal titration calorimetry; LC-MS, liquid chromatography-mass spectrometry; SAXS, small angle X-ray scattering; SDS-PAGE, sodium dodecyl sulfate polyacrylamide gel electrophoresis; TEM, transmission electron microscopy.

As mentioned earlier, when NPs are introduced into biological fluids, they may undergo various interconnected physicochemical processes, such as the formation of a protein corona and aggregation. As depicted in Fig. 11, different aspects of NPs' fate in biological media can be studied:

• Binding parameters: key metrics such as dissociation constants and cooperativity describe the protein–NP interactions. These parameters are typically derived from titration experiments, where NP concentrations are fixed while protein concentrations vary. The data from these experiments are often fit to models like the Langmuir isotherm or Hill binding to quantify binding dynamics.^307^

• Protein layer thickness and conformation: the thickness of the protein layer adsorbed onto the NP surface and the conformational changes in proteins post-adsorption provide insights into the interaction's structural impact.

• Colloidal stability: the influence of the biological media and protein adsorption on NP colloidal stability is another critical factor, as it affects NP dispersion and aggregation tendencies within the medium.

• Protein corona composition: the relative abundances of proteins within the corona are frequently analyzed following the elution of proteins from the NP surface.^308^

### 3.1. Experimental techniques for studying protein corona formation and nanoparticle colloidal stability

Depending on the specific aspect being studied, various techniques can be employed, including spectroscopic methods (*e.g.*, UV-Vis, fluorescence, circular dichroism), scattering techniques (*e.g.*, dynamic light scattering, small-angle X-ray scattering), electrophoretic methods (*e.g.*, SDS-PAGE), chromatographic techniques (*e.g.*, liquid chromatography-mass spectrometry), and microscopic approaches (*e.g.*, scanning electron microscopy, transmission electron microscopy). In the following paragraphs, we will briefly summarize the most commonly used techniques. It is important to note that the descriptions and discussions provided here do not aim to be a comprehensive treatise on characterization techniques for investigating protein corona formation and NP aggregation in complex media. For readers seeking more detailed information on these topics, we recommend consulting the following reviews^309^ and books.^310,311^

The construction of adsorption isotherms to estimate binding parameters involves determining the fractional protein coverage on the NP surface in relation to the concentration of free protein.^312^ The Hill binding equation is commonly employed to interpret the results and calculate dissociation constants and cooperativity.^312^ Ideally, these experiments are designed to assess interactions between a single protein and NPs. However, they have also been applied to more complex mixtures, such as fetal bovine serum (FBS). Generally, there are two methods for conducting these experiments: one involves the separation of NP-bound proteins from free proteins, while the other does not. In the first method, separation is achieved through centrifugation (or magnetic separation in the case of magnetic NPs). The concentration of free proteins is then quantified in the supernatant using techniques such as protein UV-Vis absorption, intrinsic fluorescence, or protein determination assays (*e.g.*, Bradford assay).^313^ Notable examples of this approach applied to MIONPs can be found in the works of Santana,^314^ Yu,^315^ and their collaborators. In the second method, a signal proportional to fractional coverage (*e.g.*, changes in size or heat) is monitored to construct the binding isotherm. Techniques such as isothermal titration calorimetry (ITC) and dynamic light scattering (DLS), which will be described below, are associated with this approach.

ITC measures the heat changes associated with the adsorption of proteins onto NP surfaces.^316^ This data allows to determine binding parameters such as association constant (*K*_a_), interaction enthalpy (Δ*H*) and stoichiometry of the process (surface binding sites). Furthermore, it allows to calculate the values of the Gibbs free energy change (Δ*G*) of the interaction and derive its entropy change (Δ*S*). Depending on the used model used to fit the data, cooperativity effects can be estimated too.^317^ Interaction of MIONPs with albumin^318,319^ or immunoglobulins,^319^ and the influence of the surface coating has been investigation using ITC.

DLS, also known as photon correlation spectroscopy (PCS), is a technique used to measure the size and size distribution of particles in a suspension. A laser beam is directed at the sample, and as particles in the suspension move due to Brownian motion, they scatter light.^320^ This scattered light is detected, and the fluctuations in light intensity are analyzed over time to derive a correlation curve. From the correlation curve, the translational diffusion coefficient of the particles is derived and further employed to calculate particle hydrodynamic size using the Stokes–Einstein equation. In situations where there is no aggregation and the signal of free protein is negligible, DLS can be used to derive binding parameters.^321,322^ It has been routinely employed in numerous works to investigate MIONPs in biological fluids, aiming to study corona thickness and detect aggregation, among others, as it is evidenced in Table 2.

Differential centrifugal sedimentation (DCS) is a technique used for the characterization and analysis of NPs in liquid media based on their sedimentation properties.^323^ In this method, a sample containing the NPs of interest is placed in a disk rotor filled with a density gradient medium (*e.g.*, sucrose) and subjected to high-speed centrifugation. As the rotor spins, the NPs sediment at different rates depending on their size and density, with larger and denser particles settling faster than their smaller and less dense counterparts. Detection methods, such as UV-visible or X-ray techniques, are used to monitor the NPs and calculate their sedimentation rates. By analyzing these rates and knowing the density of the materials comprising the NPs, researchers can derive high-resolution information regarding NP size and size distribution.^324^ DCS has been utilized to study the behavior of MIONPs with various carboxylic coatings in human plasma,^211^ as well as to determine the thickness of the hard corona formed on bare and dextran-coated SPIONs after incubation with FBS.^239^

In addition, various techniques are employed to visualize the protein corona surrounding NPs through transmission electron microscopy (TEM).^321^ The primary challenge in this visualization lies in the low contrast of the protein layers. Illustrative examples of protein corona visualization on MIONPs using TEM techniques can be found in the literature. For example, Yu and colleagues employed TEM to visualize a bovine serum albumin (BSA) corona formed around SPIONs.^325^ Similarly, Zhang *et al.*^255^ visualize the corona formed from mouse plasma onto MIONPs in their study. In both works, the authors used staining to improve contrast, uranyl staining in the former case and phosphotungstic acid staining in the latter. A dedicated section below delves into the examination of MIONPs in biological fluids using electron microscopy.

CD measures the differential absorption of left- and right-circularly polarized light by optically active (chiral) compounds. In the case of proteins, CD provides information about the secondary structure and conformational changes by analyzing the specific patterns of the CD signal in the wavelength range *ca.* 190–260 nm.^326^ It can be used to check the influence of protein–NP interactions in the conformation of the former. For instance, in the field of MIONPs, Ashby *et al.* found that non-binding or fast dissociating proteins did not undergo secondary structure changes upon interaction with SPIONs, while slow dissociating proteins exhibited clear alterations of their native structure.^327^ On other example, Yallapu and co-workers^328^ demonstrated that the conformation of human serum proteins was only slightly to negligibly affected after interacting with MIONPs coated with cyclodextrin and F127 polymer.

In small-angle X-ray scattering (SAXS), the sample is exposed to X-rays, and the scattering pattern of these X-rays is measured at low angles. SAXS provides valuable information about the size, shape, and arrangement of NPs, including aggregation features, at the nanoscale.^329^ This experimental technique can be used to assess the colloidal stability of NPs in biological fluids, as well as the nature of aggregates when they are present.^330^ Moreover, it can be employed to follow protein adsorption and derive binding parameters.^321^ Honecker *et al.*^331^ have reviewed the use of SAXS (as well as SANS, small angle neutron scattering) for the characterization of magnetic NPs and provided commentary on several examples related to MIONPs.

Sodium Dodecyl Sulfate Polyacrylamide Gel Electrophoresis (SDS-PAGE) is a widely used laboratory technique for separating and analyzing proteins based on their molecular weight.^332^ In SDS-PAGE, proteins are denatured and coated with a negatively charged detergent (SDS) to give them a uniform negative charge. They are then subjected to electrophoresis in a polyacrylamide gel, with smaller proteins moving faster than larger ones through the gel matrix in response to an applied electric field. As a result, the proteins are separated according to their size, forming distinct “bands” on the gel. After electrophoresis, the gel can be stained to visualize the separated proteins. It is routinely employed for studying qualitatively or semi-quantitatively the composition of protein coronas adsorbed around NPs. In this case, proteins are commonly eluted from NP surface using specific buffers and then submitted to the electrophoresis run.^333^ Several examples of the use of SDS-PAGE for characterizing protein coronas formed on MIONPs having different surface functionalization and exposed to distinct protein sources (*e.g.*, FBS, plasma, among others) can be found in the literature.^50,214,217,218,220,251,334–341^

### 3.2. Mass spectrometry

Mass spectrometry (MS) is commonly employed to elucidate detailed information about the composition of the protein corona. MS identifies and quantifies molecules based on their mass-to-charge ratio. Various MS techniques are available, depending on the method used to ionize the sample (*e.g.*, electrospray ionization, ESI, or matrix-assisted laser desorption/ionization, MALDI) and the approach to analyzing the mass-to-charge ratio (*e.g.*, time-of-flight, quadrupole, ion trap, Orbitrap).^335^ In the analysis of protein coronas, two main approaches are employed. In the first approach, the corona is eluted from NPs (NPs), subjected to SDS-PAGE, and bands of interest are trypsin-digested into peptides, which are subsequently analyzed using MS. Alternatively, in gel-free approaches, the eluted corona is trypsin-digested, the resulting peptides are separated using liquid chromatography (*e.g.*, reverse-phase C18 columns), and then submitted to MS (LC-MS). Intermediate approaches are also prevalent, wherein bands excised from SDS-PAGE gels are subjected to trypsin digestion and subsequently analyzed using LC-MS. The identified peptides are then matched to entries in protein sequence databases.^336^ Following data processing, this enables the detection and quantification of the relative abundances of individual proteins in the corona.^333^ In the field of MIONPs, mass spectrometry, particularly LC-MS, has been extensively utilized to assess protein coronas. For example, Sakulkhu and colleagues investigated, using LC-MS, the influence of distinct PVA and dextran-coated SPIONs on the composition of the protein corona and its impact on blood circulation time.^214^ Portilla *et al.*^251^ explored the composition of the protein corona formed on MIONPs with different coatings (APTES, dextran, or DMSA) exposed to fetal bovine serum (FBS) in various cell culture media. They correlated their findings with the interaction of the NPs with macrophages. In another investigation, Wang and co-workers^334^ assessed the composition of the protein corona formed on MIONPs coated with brushed PEG or brushed phosphorylcholine using LC-MS and evaluated how it altered the catalytic activity of the NPs.

### 3.3. Electron microscopy: transformation studies at different scales

**Fig. 12:**
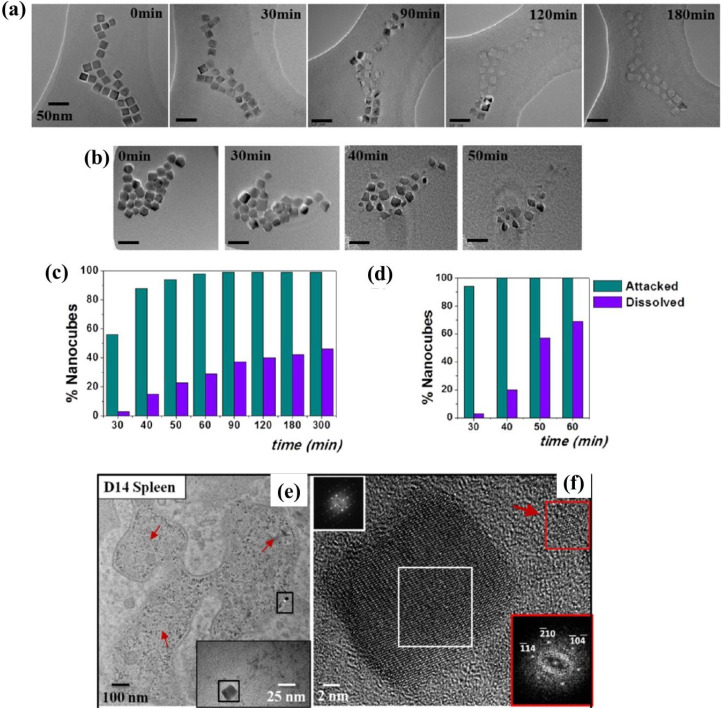
Biodegradation of polymer functionalized nano-cubes; biotransformation of (a) amphiphilic, (b) PEG, coated iron oxide nano-cubes in the model lysosome-like medium. The rate of nano-cubes modulation over time for (c) amphiphilic, (d) PEG coatings. (e and f) *In vivo* distribution and recycling of nanocubes. Reproduced from ref. 50 with permission from American Chemical Society, Copyright 2013.

The development of efficient and safe nanomaterials for biomedical applications requires a comprehensive understanding of their lifespan in living organisms. For this purpose, multifunctional TEM provides the opportunity to study the interactions of injected nanomaterials in the body, their life cycle, and changes in structure and chemical properties. The first study using high-resolution TEM was reported by Lartigue *et al.*^50^ in 2013, focusing on iron oxide nanocubes with two different coatings: amphiphilic and polyethylene glycol (PEG) polymer. The studies were performed at different levels of complexity, including model lysosomal medium, *in vivo*, and *in vitro* (Fig. 12a–f). High-resolution images showed that the distribution of the coating layer was important for the transformation of the iron oxide nanocubes, as the majority of the cubes were attacked from the corners rather than the facets. Furthermore, the nature of the coating also influenced the transformation process, with PEG-functionalized cubes degrading more rapidly than those coated with amphiphilic materials. These transformations significantly affected biological-related properties, such as heat generation efficiency and contrast-related characteristics. *In vivo* studies revealed similar behavior, indicating a stochastic process of degradation. Research on iron oxide nanoflowers by the same group demonstrated that porous surfaces in nanomaterials could act as preferential sites for degradation. Although the shape of the nanomaterials did not provide many indicators of selective degradation, the nature of the surface remained crucial. For comparison, the transformation of iron oxide–gold dimers was studied through TEM, revealing that the degradation of iron oxide occurred much faster than that of the gold component. There was no degradation of the gold portion observed until one month after injection, whereas degradation of gold was noted *in vivo* after six months. Iron oxide-filled carbon nanotubes also exhibited degradation *in vivo*, with the carbon components progressively annihilating while unpacked iron oxide was released from within.

Recent studies have exploited that TEM revealed the biodistribution of nanomaterials (such as plasmonic, magnetic, and graphitic nanostructures) along with their biodegradation and recycling processes for biomedical applications. Cho *et al.* studied the kinetics of different-sized PEG-coated gold NPs *in vivo*. For 24 h, gold NPs with the size (4 or 13 nm) revealed a high level in blood but cleared after 7 days. However large particles with the size range of 100 nm showed complete clearance after 24 h. TEM studies revealed that AuNPs are trapped inside lysosomal Kupffer cells, cytoplasmic vesicles, mesenteric lymph, and macrophages of the spleen. Small-sized gold NPs activated transiently 2B and CYP1A1 enzymes, metabolic enzymes of phase I in the liver tissues for the time duration of 1–7 days, which confirmed the increased gold levels inside the liver. TEM micrographs confirmed that Fe is accumulated substantially in the macrophages of the connected tissues and delivered eventually to different parts of the organism. Balasubramanian *et al.* examined the biodistribution of AuNPs with the size range of 20 nm in more than 25 different organs for 24 h, 1 week, 1 month, and 2 months after a single-dose administrated intravenously in rats. Gold was consistently and rapidly accumulated inside the spleen (8.4 ± 5.0 to 9.5 ± 6.4 ng g^−1^) and liver (49.4 ± 50.4 to 72.2 ± 40.5 ng g^−1^) for 2 months of study.^342^ Lévy *et al.* studied the degradability of ultrasmall superparamagnetic MIONPs and observed a slight shift in size distribution towards smaller size. The diffraction results confirm the structure retainability of the NPs (*i.e.*, magnetite/maghemite structure).^338^

### 3.4. Microscale follow-up of biotransformation

#### 3.4.1. Magnetic properties follow-up

**Fig. 13:**
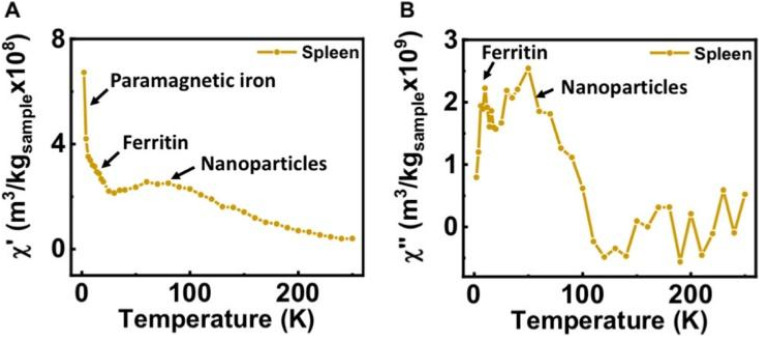
Variation in AC magnetic susceptibility with temperature of spleen indicating the role of different iron-based species, (A) in-phase component, (B) out of phase component. Reproduced from ref. 344 with permission from American Chemical Society, copyright 2022.

A vibrating sample magnetometer (VSM) is a valuable tool for quantifying changes in magnetic properties. In this technique, the magnetic field of the sample is altered due to the vibration of components, which generates an electric field in the coil based on Faraday's law of induction. Villanueva *et al.*^339^ reported the modification of MIONPs with anionic, cationic, and neutral-charged carbohydrate molecules (∼10 nm) and designed a delivery system targeting tumor cytokines. Mazuel and co-workers^340^ proposed the use of stem cell spheroids to track intracellular transformations within tissues, presenting the spheroid's magnetic properties as an indicator of the degradation process. This same spheroid model was employed for tracking the biodegradation of multifunctional iron oxide–gold nanoparticles by measuring variations in magnetic properties following a 1 month intravenous injection. While the magnetic properties of the Fe core degraded rapidly, the gold component preserved the magnetic core and its related technological properties.^341^ Ruiz *et al.*^343^ conducted *in vivo* studies using murine models for 3 months and reported the accumulation of nanoparticles in the liver, spleen, and lungs without significant toxic effects. However, biotransformation was observed, manifesting as either size reduction or aggregation of the particles. In contrast to DMSA-coated nanoparticles, which metabolized within 24 h in the liver and lungs, PEG-functionalized nanoparticles were detected even after 24 h in various organs. This suggested a longer circulation time for PEG and a delayed approach of the particles to the tissues. The loss of saturation magnetization was also reported as an indicator of the biodegradation process.^338^ AC magnetic susceptibility has been validated through histological and iron protein analyses, demonstrating its utility as a valuable technique for quantifying magnetic nano-species *in vivo*.^343^ Additionally, magnetic susceptibility was utilized to differentiate the degradation kinetics of glucose and PEG-coated MIONPs in various organs, including the spleen, liver, heart, lungs, and kidneys.^215^ Fernández-Afonso *et al.*^344^ extensively employed AC magnetic susceptibility for Fe profiling in animal tissues by quantifying magnetic properties, ferritin proteins, and related Fe species. This technique allows for the quantification of large tissue amounts with high sensitivity and a low limit of detection for Fe species (Fig. 13).

#### 3.4.2. Quantification of material by ICP-MS

Inductively coupled plasma atomic emission spectroscopy significantly plays a major role in pharmaceutical analysis. It is considered a multi-elemental analysis technique but provides accuracy only at ultra-trace levels with complex spectral interfaces. It is used for the determination of multiple elements of heavy metals in ultra-trace and trace concentrations in pharmaceutical and bulk drugs. Krone *et al.*^345^ detected Cd in many Zn supplements through ICP-MS, with gluconate supplements containing the lowest Cd amounts. Bourgoin *et al.* detected Pb and Cd in calcium supplements using ICP-MS.^346^ Wang *et al.*^347^ determined tungsten and its intermediates in bulk drug substances. The minimization of matrix effects was achieved through the recovery of spiked samples. Different isotopes were used for monitoring spectral interfaces. The detection limit was 0.000246 g mL^−1^, and the linear method was used from 0.1 to 5 g mL^−1^. Spikes were recovered at 5, 10, and 50 ppm levels, respectively. Bismuth was used as an internal standard. In the tungsten samples, this method covered approximately the range of 2500 ppm. Lewen *et al.*^348^ reported traces of Ni, Pb, Sn, and Cr in vitamin E through ICP-MS. Microwave emulsion and acid mineralization were compared; acid mineralization with hydrogen peroxide and nitric acid was carried out in microwave vessels. Triton X acted as an emulsifier for the preparation of the emulsion. To decrease viscosity, tetralin was added. Ti, In, and Y were used as internal standards. Matrix effects were analyzed through the comparison of slopes obtained by external calibration and standard addition. I, Pd, Br, Ba, and Na were determined quantitatively in methamphetamine hydrochloride through two different methods involving reagents and catalysts. Pd, Na, Ba, and Br were determined in the drug prepared using Emed's method. However, Br, Na, and I were determined in the drug prepared by Nagai's method.^349,350^ Lewen *et al.*^351^ detected Se, In, As, Cd, Sb, Bi, Pt, Ag, Hg, Pd, Ru, and Mo through ICP-MS. Four solvents out of seven were considered the best: water, 2-butoxy water/ethanol, 0.5% HCl, and 5% HNO_3_. Many APIs were insoluble in 0.5% HCl; hence, 2-butoxy water/ethanol was preferably used. Internal standards of Au, Co, and Rh were added to a standard solution of 25 ng mL^−1^. The instrument was tuned to 25 ng mL^−1^ of indium solutions. The average recovery of specific elements in accordance with functional groups was studied through ICP-MS.

#### 3.4.3. Quantification of superparamagnetic iron by electron spin resonance

Electron spin resonance spectroscopy (ESR), also called electron paramagnetic resonance or electronic magnetic resonance, is used for the detection of molecules with unpaired electrons, such as paramagnetic transition metal ions (Fe^3+^, Cu^2+^, Mn^2+^)^352^ and free radical species. ICP-MS can be used for the quantification of Fe and other metals in the body, but the problem lies in the fact that ICP-MS cannot differentiate between iron oxide from NPs and Fe that is already present in the body. The ESR/EPR technique can be useful as it can detect only SPIO (*i.e.*, from NPs); therefore, ESR spectroscopy has many applications in various fields, such as food sciences, physics, biological sciences, and chemistry. Immunoassays have tremendous applications in both industrial quality control and medical diagnosis. Large analytical methods have been proposed for immunoassay development to diagnose many infectious agents, drugs, proteins, hormones, and peptides.^353^ The most popular immunoassays include radioimmunoassay,^354^ chemiluminescence,^355^ enzyme immunoassay,^356^ surface plasmon resonance^357^ and fluorescence immunoassay.^358^ Iannone *et al.* investigated superparamagnetic macromolecular magnetite complexes coated with hydrophilic dextran as a contrast agent in MRI in the spleen and liver. The authors detected the dextran magnetite particles in tissues through electron spin resonance spectroscopy. They injected dextran magnetite NPs into a group of experimental animals, and these were detected through ESR in some of the reticuloendothelial organs. The ESR spectrum of the complexes revealed the distribution of these particles in blood, liver, bone marrow, and spleen as a function of time, concluding that blood clearance was biphasic and dependent on particle size.^359^ Jiang *et al.* applied ESR to immunoassays and used MIONPs as probes in ESR to amplify the signals resulting from Fe^3+^ ions present in the lattice. To detect the antigen, a polyclonal antibody of IgG from rabbits was used. MIONPs with a size range of 10–30 nm were assigned to the antibody, while Fe^3+^ ions in the NPs were investigated for the ESR signal. With rabbit IgG, Sepharose beads were assigned as the solid phase. Antibody-labeled NPs were added to a sample containing the antigen, and then Sepharose beads conjugated with the antigen were introduced into the sample. Antibody-labeled NPs bound to the antigen present on the Sepharose beads were centrifuged to separate them from the sample for measurement. Smaller-sized NPs, in the range of 10 nm, probed the upper detection limit and sensitivity up to 40 and 1.81 μg mL^−1^. With the increase in size, the detection limit and sensitivity ranges decreased to 0.014 and 0.13 μg mL^−1^.

### 3.5. Factors affecting the transformations in the body

#### 3.5.1. Nature of nanomaterials

Nanomaterials, owing to their promising physical and chemical properties, have raised significant concerns regarding their impact on the environment. Their safe implementation at the industrial level, as well as in nanomedicine, requires a thorough understanding of their behavior in organisms. Due to the high surface-to-volume ratio, they undergo physical and chemical transformations, corrosion, and degradation triggered by interactions with the surrounding environment.^360,361^ Biological interactions are quite complex because of the involvement of biomolecules, which greatly affect their physical state, properties, chemical evolution, and aggregation. Surface reactivity is an important factor in determining the outcomes and hazards of NPs in living organisms. To assess the toxicity, transport, and persistence of inorganic nanomaterials, their transformation is a critical factor. Oxidation or reduction of metal oxide NPs and elemental metals occurs in natural systems through various interactions with different ligands, resulting in the formation of various toxic ions, such as Zn^2+^, Fe^2+^, or Ag^+^.^362^ Lartigue *et al.*^50^ reported the visualization of the transformations of iron oxide nanocubes influenced by the intracellular environment. They monitored all possible structural degradations of nanocubes coated with polyethylene glycol and amphiphilic polymer shells using aberration-corrected TEM. Their results showed that polymer coatings exert significant control over the availability of chelating agents and surface reactivity, which govern the degradation process. A comparison of single nanocube degradation studies with intracellular transformations in mice observed over two weeks after intravenous administration comprehensively revealed the role of intracellular sorting within degradation compartments, as well as NP clustering, recycling, and transport of ferritin in the protein. Their approach reduced the gap between in-solution and *in vivo* tracking.^50^

#### 3.5.2. Alternating current biosusceptometry

To attain the real translational of MIONPs prospective after administered in an organ, they must be identified *in vivo* as well as real time to evaluate the various physiological factors along with their possible interaction, retention and elimination profiles. Several MNPs distributions studies performed by various research groups, however the process of uptake and subsequent metabolization and degradation of MIONPs by mononuclear phagocytic system remains unknown. Prodan *et al.* reported that the long-term accumulation may be equally interesting and beneficial for imaging purposes as well as for other therapeutics applications,^363^ where they act as a *T*_2_ contrast enhancement agent in MRI either as a tracer or marker of new imaging modalities such as multichannel-ACB imaging.^364^ Further, a pre-clinical trial must be considered for future and real clinical applications, once the long term effects of the MIONPs deposited in the liver and spleen are still unknown.^192,365^ It should be kept in mind that MIONPs retention has long side effects for the periods up to 10–11 months in the organs.^366^

**Fig. 14:**
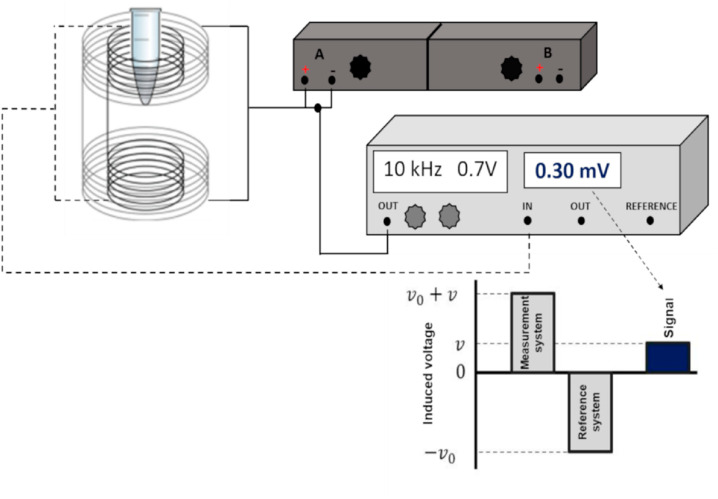
Schematic view of ACB setup used for MNP detection and quantification. A phase-sensitive amplifier (lock-in—Stanford Research Systems SR830) (light grey), an electrical signal of 0.7 V at a frequency of 10 kHz is generated and is amplified by power amplifiers (−3 dB) (dark gray), in which the resulting current is applied to the excitation coils. Reproduced from ref. 367 under a Creative Commons (CC BY) Licence from MDPI, Copyright 2022.

In the recent time, various imaging, spectroscopy and magnetometry techniques are employed for the detection and quantification of the biodistribution of MNPs in animal models along with MRI and MPI. Alternating current biosusceptometry (ACB) is a simple biomagnetic detection system used to detect and quantify the amount of MNPs in different organisms. It is based on the mutual induction between two induction and pickup coils coaxially arranged in a first order gradiometer. When there is no magnetic material close to the measurement system, the signal response is minimized. By closing the gap between the magnetic materials and detection pair, an imbalance occurs in the magnetic flux, increasing the electrical signal acquired. This signal can be measured, digitalized and recorded online with the help of a sensitive-to-phase amplifier, analog/digital card and a computer. A more details about the ACB system for MNPs biodistribution and elimination signal quantification is given elsewhere.^367^ A schematic diagram of the ACB set up is shown in Fig. 14.

**Fig. 15:**
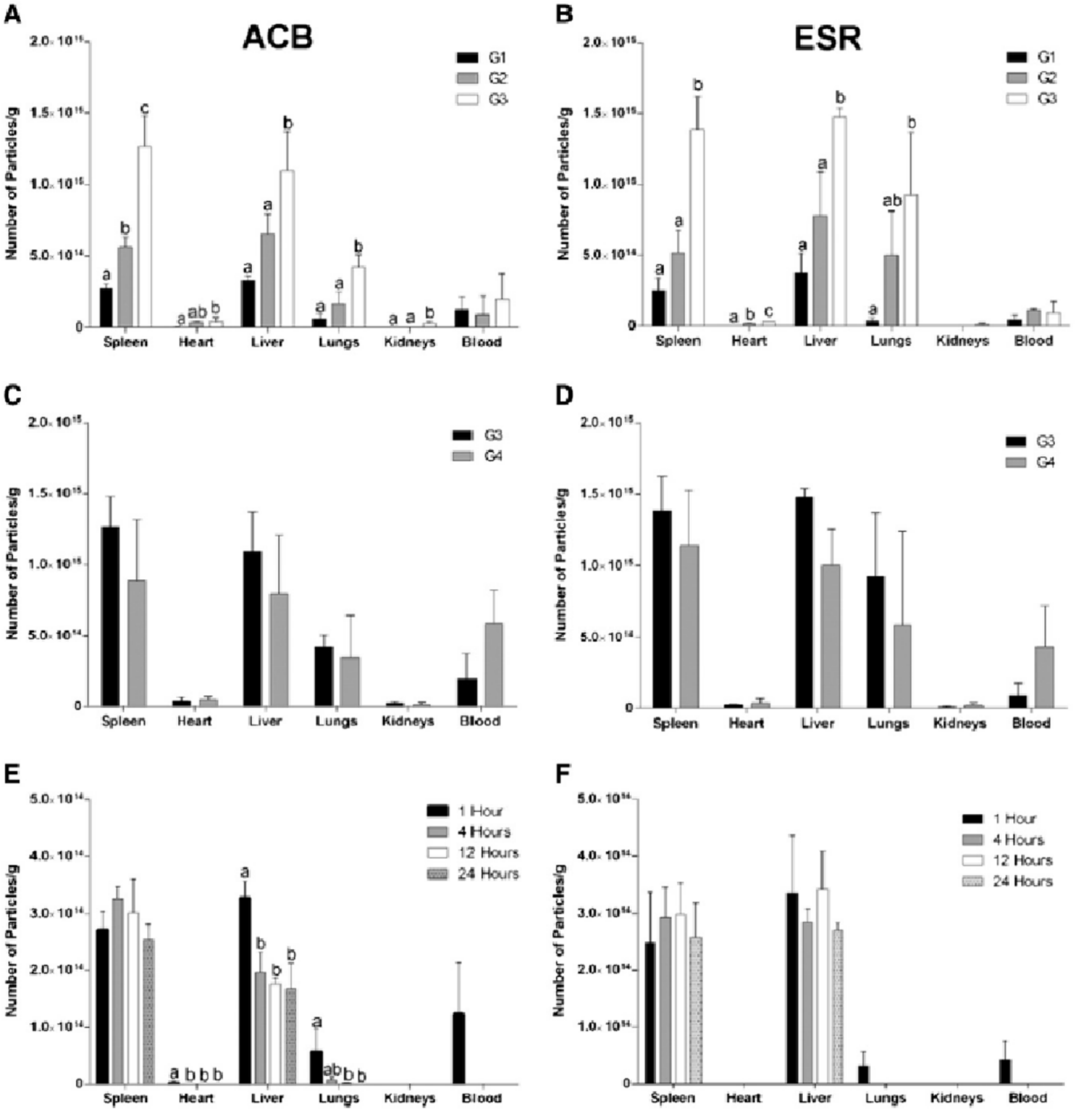
(A) ACB and (B) ESR data for MNP biodistribution in G1, G2, and G3. (C and D) Comparison between G3 and G4 for the ACB (C) and ESR (D). (E and F) Biodistribution results and changes due to time for ACB (E) and ESR (F). The results are expressed as mean and standard deviation. Same letters represent no significant difference, whereas different letters indicate significant differences between groups (*p* = 0.05). Reproduced from ref. 369 under a Creative Commons (CC BY) Licence from Springer Nature, Copyright 2017.

Quini *et al.*^368^ used ACB approach to evaluate the dynamic of citrate coated MnFe_2_O_4_ NPs distribution by assessing liver uptake and its response to change in the dose, administration protocols and times. Sixteen male rats (weighing 250–300 g) were divided into four groups and received saline (0.9 mg mL^−1^; control), and one (G1), two (G2), or three (G3) 300 μL injections of MNPs (23 mg mL ^−1^, 1.17 × 10^15^ NPs per mL, dispersed in saline solution). Fig. 15 shows the biodistribution results including the number of particles that were found in each organ of system G1, G2, and G3 by ACB and its comparison by ESR. Moreover, to access how the injection protocol interferes with accumulation of NPs, four additional rats (G4) received a single injection of 900 μL. The results obtained from both systems for each organs using paired *t*-test were compared. No significant variations between the system were observed. All the groups presented an upsurge in the spleen, liver, blood for shorter time points. However, beyond 4 h of the MNP injection, the particle blood concentration approached to zero.

The same research group further monitored the citrate coated MnFe_2_O_4_ NPs in the bloodstream of Wistar rats using two different injection protocols by ACB. They simultaneously evaluated cardiovascular parameters, including mean arterial pressure, heart rate, and episodes of arrhythmia in order to secure the well-being of all animals.^369^ It was observed that serial injections of MNPs increased the circulation time in comparison to single injections. Immediately, after each injection, it was observed a transitory drop in arterial pressure, a small drop in heart rate, and no episodes of arrhythmia. Though some cardiovascular effects were observed, they were transitory and easily recovered in both protocols.

Soares *et al.*^367^ has used the ACB system to study a DOX-induced chronic kidney disease (CKD) model in rats, *in vivo* and real time, as well as in *ex vivo*. The kidney perfusion and washed-out profiles of healthy animals from injured kidney animals were successfully demonstrated through biochemical and histological study. Through ACB study, it was observed that DOX caused an important effect on NP biodistribution, indicating a generalized modification in biodistribution as the disease evolved. The dynamical properties of perfusion in progressive kidney disease showing a great potential to reach clinics.

**Fig. 16:**
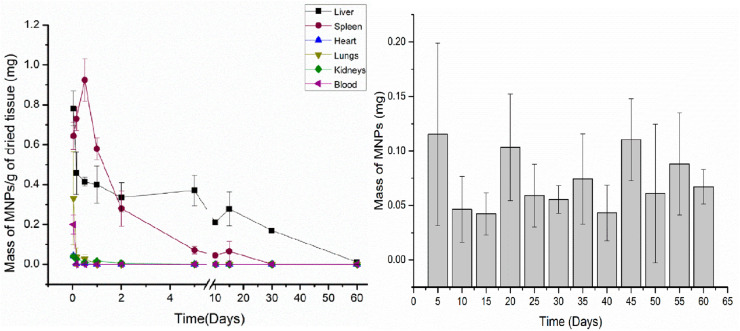
Biodistribution results for all organs of interest of the Cit-MnFe_2_O_4_ over the period from 1 h to 60 days (left panel); elimination *via* feces every five days (right panel). For statistical analysis, the Mann–Whitney *U* test was used. It was found no significant difference between the days (*p* < 0.05). Reproduced from ref. 367 under a Creative Commons (CC BY) Licence from MDPI, Copyright 2022.

The same group has explored the biodistribution of the same NPs (Cit-MnFe_2_O_4_) with hydrodynamic diameter of 40 ± 5.6 nm and *ζ* = −27.8 mV in different organs quantified from 1 h to 60 days after *in vivo* administration through ACB technique. Fig. 16 shows the biodistribution results for all organs of interest with dominate accumulation in the liver and spleen, Cit-MnFe_2_O_4_ were in all the organs from 1 h and until 12 g after the administration. However, in the liver, the NPs were detected in the whole span of time (*i.e.*, 60 days), presenting a maximum after 1 h of administration (∼5.4 mg of Cit-MnFe_2_O_4_).

It was found that amount of administered Cit-MnFe_2_O_4_ reached a low level (approximately zero) after 60 days. In the spleen, MNPs were detected significantly within 15 days of administration, with the highest accumulation observed at around 12 h (0.092 mg). The Cit-MnFe_2_O_4_ signal was ultimately found to decrease over time. Despite low ACB signal intensity, Cit-MnFe_2_O_4_ was also observed in other organs, such as the kidney (detectable up to 48 h), whereas both the heart and lungs accumulated NPs only up to 12 h. Furthermore, the authors noted that elimination kinetics showed an exponential pattern, with the liver and spleen as the two main organs responsible for NP uptake from the bloodstream. The pharmacokinetics of the MNPs in both organs were compared, demonstrating a bi-exponential model to determine the circulation time of Cit-MnFe_2_O_4_. In the liver, Cit-MnFe_2_O_4_ exhibited bi-exponential concentration decay with a first-phase half-life of 70 min, which was faster and responsible for the distribution and clearance of most of the injected dose. The second phase was slower, with a half-life of approximately 30 days. In the spleen, however, a single phase with a half-life of around 2 days was observed. Quantification of Cit-MnFe_2_O_4_ NPs in feces was reported five days post-administration, showing a peak value of 0.115 ± 0.08 mg of NPs, although without any consistent trend (Fig. 16, right panel).

#### 3.5.3. Magnetic resonance imaging

**Fig. 17:**
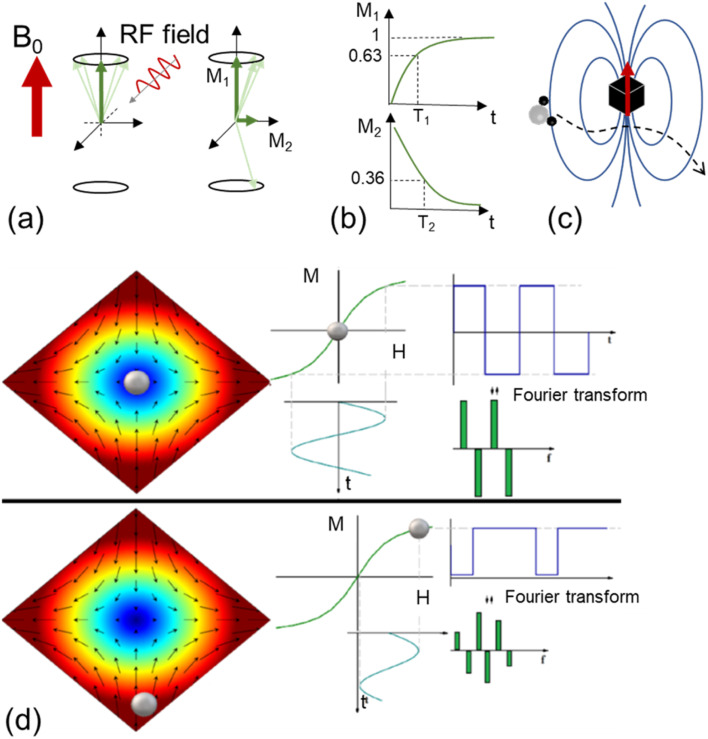
Physical principles of MRI: (a) a strong magnetic field (*B*_0_) is applied in the longitudinal direction and a perturbing RF field pulse is applied in the transverse direction, (b) the relaxation of the longitudinal (*M*_1_) and transverse (*M*_2_) magnetization has characteristic times (*T*_1_ and *T*_2_ respectively) and (c) magnetic field inhomogeneities produced by CAs are responsible for variations in the relaxation rates. Basics of MPI: (d) spectral response at a given location of the magnetic tracer within the selection field: the tracer is located at the FFP (upper panel), the tracer is placed in a point within the selection field such that it reaches the saturated magnetization state (lower panel). Reproduced from ref. 22 with permission from American Chemical Society, Copyright 2015.

MRI has great potential to follow their biodistribution of magnetic NPs in the body as a result of their usage as contrast agents (CAs).^1,2^ MRI is a fundamental non-invasive medical diagnosis tool that relies on the relaxation of the spin of hydrogen nuclei. Since the relaxation rates are influenced by the proton diffusion velocity, which depends on the structure of the diffusing molecules, contrast between water and fat can be achieved.^4^ This contrast can be enhanced by means of the introduction of CAs, *i.e.*, substances that alter the proton relaxation rates. There are two types of contrast agents depending on the MRI acquisition method: longitudinal (*T*_1_-weighted) or transverse (*T*_2_-weighted). In both cases a strong magnetic field is applied in the longitudinal direction and a perturbing radiofrequency field pulse is applied along the transverse direction. The perturbing field flips the magnetization to the perpendicular direction which, once switched off, relaxes to the original direction given by the static field. Two processes can be thus followed: the relaxation of the longitudinal magnetization to its original value (*T*_1_-weighted images) or the fast decrease of the transverse magnetization (*T*_2_-weighted images) (see Fig. 17a–c). The presence of paramagnetic molecules with high magnetic moments or field inhomogeneities (produced, *e.g.*, by superparamagnetic NPs) can shorten the nuclear spin relaxation times, increasing the contrast in the images. Most *T*_1_-weighted CAs are based on paramagnetic Gd^3+^-based complexes^5^ while *T*_2_-weighted images can be obtained by using superparamagnetic (SPM) NPs, mostly Fe oxides due to their biocompatibility. In the latter, the high susceptibility of the NPs produces magnetic field inhomogeneities and field gradients close to the particles, leading to a dephasing of the proton spins.^2^ Since the early proposal of using SPIOs^6^ as contrast agents for liver MRI, many studies have focused on designing novel CAs with increased efficiency.^7,8^ Compared to paramagnetic complexes used for *T*_1_-weighted imaging, due to their specific size or surface ligands, MIONPs can accumulate in certain organs or can target specific cells,^9^ which could be important for the development of more efficient diagnosis tools.

Several studies focused on the design of NP systems for novel *T*_2_-CAs. Some attempts to increase the relaxivity (*r*_2_, *i.e.*, the reciprocal of the transverse relaxation time) have relied on tuning the composition of the oxide to achieve a larger magnetic moment while keeping a SPM response.^11,71^ However, this strategy is limited due to toxicity issues of other metal cations such as Co(ii). On the other hand, it was shown that an increase in the size of the NPs often leads to increased *r*_2_ values^12^ but at the expense of larger hydrodynamic sizes that can lead to changes in the biodistribution of the NPs. Record-high values have been achieved by introducing nanocrystals with anisotropic shapes (*i.e.*, nanocubes and nanostars) which are responsible for enhanced magnetic fields inhomogeneities in the vicinity of the NPs.^14,47^ Such optimization of the Fe oxide size, composition and morphology has been possible following the development of thermal decomposition methods that have allowed a precise control of the crystallinity, size and shape of the nanocrystals, as discussed in Section 1.

Apart from the size and composition of the inorganic core, the efficiency of a *T*_2_-CA is also governed by the ligands at the NP surface. This is due to the fact that diffusion of water molecules can be mediated by the interaction with such ligands. For example, highly hydrophilic molecules can slow down the diffusion process, shortening *T*_2_ and increasing the relaxivity, while hydrophobic entities can produce an opposite effect.^12,69^ However, bulky ligands also produce an increase in the hydrodynamic size and, above a certain size, the interaction of protons with the magnetic core can be diminished, lowering the relaxivity. There exists therefore a compromise between the size of the NP and the hydrophilicity of the capping molecules. According to recent systematic studies of precisely controlled NPs with different sizes and capping layers,^12,370^ small particles would benefit from an increase in the capping thickness or hydrophilicity, while the relaxivity of large particles is decreased for thicker coatings.

#### 3.5.4. Magnetic particle imaging

The detection of NPs through MRI is indirect since it is mediated by their interaction with water protons so there is interest in developing imaging techniques able to quantitatively detect magnetic NPs as a result of their physical properties^16^ (Fig. 17d). Magnetic particle imaging (MPI) is a relatively new technique that takes profit of the magnetic properties of a magnetic NP system first presented by Gleich and Weizenecker.^17^

The basic principle follows the theory of superparamagnetism.^18,19^ Briefly, when an external magnetic field is applied to the system, the magnetic moments of the NPs align coherently with the field orientation, and the sample magnetization is given by *M*_s_ = *μN*, where *N* is the number of NPs in the sample and *μ* is the mean magnetic moment per NP. At finite temperature, in the absence of an applied field, the magnetic moment of each NP oscillates between stable states separated by a magnetic anisotropy energy barrier. Generally, a uniaxial anisotropy is considered, which provides two possible stable states. The average time it takes for the NP magnetic moment to transition from one stable state to another is known as the Néel relaxation time. Additionally, magnetic relaxation of NPs in a colloidal suspension can occur *via* Brownian relaxation, which involves the physical rotation of the NPs (and their magnetic moments) within the colloid. Depending on the properties of the NPs and the experimental conditions, relaxation in a colloidal suspension is governed by one of these two mechanisms. Large magnetic anisotropies favor the Brownian mechanism, while if the thermal energy is sufficient to achieve thermodynamic equilibrium of the MNPs, the Néel relaxation will dominate the magnetization dynamics.

When an alternating magnetic field is applied to the system, the relationship between changes in the magnetization of the NPs and the field direction is nonlinear. In other words, there is a discrepancy between the applied field frequency and the NPs' relaxation time.^17,20^ Consequently, the measured magnetization generates a series of harmonics, which are fundamental to the MPI technique. For a sufficiently low applied magnetic field, the presence of harmonics confirms the existence of magnetic material and can be used to distinguish regions containing magnetic particles from those without NPs. This principle is therefore useful for determining the spatial distribution of magnetic material within a given sample. To map the spatial distribution within a sample, a DC external field is required to ensure that the particles are in a magnetically saturated state. This field, known as the selection field, is typically configured in a Maxwell arrangement. Within the Maxwell magnets, a zero-field magnetic point called the field-free point (FFP) exists, where the magnetic material responds to the alternating external field, producing measurable harmonics in its magnetization dynamics. Conversely, if the magnetic material lies outside the FFP, the magnetization remains in a saturated state, and no harmonics are detected. By moving the FFP across the sample, a map of the magnetic material's distribution can be generated.^17,21–23,371^

In recent years, advancements in MPI have focused on the development of magnetic tracers. The resolution and signal-to-noise ratio of an MPI measurement strongly depend on the magnetic and structural properties of the NPs. Key factors influencing the response of an MPI tracer include the mean size and size distribution of the NPs, saturation magnetization, coercivity at experimental temperatures, anisotropy, particle aggregation, and surface capping.^22,23^ NP size is well-known to correlate closely with magnetic properties such as saturation magnetization, coercivity, and magnetic interactions. These properties directly affect NP relaxation and, consequently, the signal obtained from the retrieved harmonics; studies indicate that an optimal frequency exists for a given NP size.^16,24^ Additionally, surface ligands on NPs can reduce dipolar interactions and prevent aggregation. Since the loss of colloidal stability affects magnetic properties, it is a critical factor in designing effective MPI tracers. Biodegradation of NPs may also alter their magnetic response, and the capping layer can play an essential role in this process. Overall, similar to MRI, MPI uses non-ionizing radiation and can provide a signal that scales with the magnetic NP concentration, offering competitive spatial and temporal resolution.^16^*In vivo* studies have shown the application of MPI to track stem cells^25^ and to follow quantitatively the short-term biodistribution of commercial tracers.^26^

#### 3.5.5. X-ray microscopy

The recent developments in X-ray microscopy (XRM) have driven the technique into a promising tool for imaging both inorganic materials and biological tissues,^372,373^ meaning it can be deployed to track and characterize iron-rich particles in a biological environment.^374–376^

**Fig. 18:**
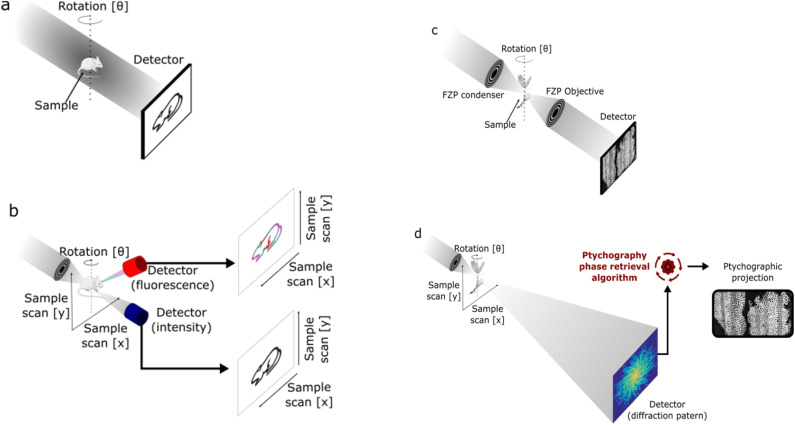
Main XRM techniques: (a) X-ray μ-tomography; (b) scanning X-ray microscopy including STXM and SXFM; (c) full-field X-ray microscopy; (d) X-ray phychography.

Particularities of the XRM technique would include not only their spatial resolution but also the high penetration allowing 3D imaging. Technically, X-ray imaging can be traced back to the discovery of the X-ray itself, as celebrated by the 1901 physics Nobel prize. By then, the obtained images consisted of pure absorption contrast 2D projections. Later, in the 1970s, the development and establishment of X-ray tomography as a robust and reliable 3D imaging technique culminated into the main feature found at XRM. Those days, this more familiar approach translates into what is known as μ-tomography (Fig. 18a) in which a small pixel X-ray image detector can acquire micrometre resolution projections and thus allow tridimensional imaging with similar resolution.

Further, better-resolved images require a different development of XRM. One of its first and conceptually simplest implementations was the scanning transmission X-ray microscopy (STXM) (Fig. 18b),^377^ where a focused probe scans across the sample, and the transmitted X-ray is measured at all scanned points. Thus, STXM would be X-ray equivalent to scanning transmission electron microscopy (STEM) as it does not rely on an objective lens after the sample for imaging but only on the bi-dimensional correlation between the beam position and the measured intensity. Besides the absorption contrast of X-rays, which includes X-ray absorption spectroscopy (XAS) contrast,^378^ a scanning X-ray probe also allows for more advanced contrast imaging such as X-ray fluorescence,^379–381^ diffraction,^382–384^ scattering,^385–389^ and even X-ray magnetic dichroism.^376^ In all those cases, the measured bi-dimensional image can be interpreted as a projection image that itself can compose a tomogram allowing for a respective advanced contrast 3D X-ray imaging.

All of those techniques can, in principle, be used to trace different aspects of the nanomaterial ones inside the biological environment. This not only allows the characterisation of the inorganic material but also adds context to the biotransformation being observed with various spatial resolutions. However, most of them are still under development and lack use in general-purpose studies aiming the biotransformation of Fe particles. Still, techniques that exploit X-ray spectroscopy elemental sensitivity, like XAS implemented on a STXM and especially scanning X-ray fluorescence microscopy (SXFM) provide valuable information on the chemical state of NPs. Over the past decades, SXFM (Fig. 18b) has established itself as a method for investigating naturally occurring elements,^390–392^ drugs,^393,394^ and NPs^395,396^ inside tissues and cells with variable special resolutions capable of resolving NP clusters.

However, if the desired experiment targets a single NP and the biological context is limited to a single cell, another XRM technique can contribute to the visualisation of iron NPs. Soft X-ray XRM (Fig. 18c) uses low-energy X-rays at the so-called “water window”^397^ and Fresnel zone plate objective lens capable of magnifications up to 100 times.^378^ The presence of an objective lens creates an X-ray microscope similar to an optical microscope, capable of achieving resolutions of 25 nm for biological samples. Also similar to an optical microscope, the fundamental contrast mechanism for the formation of images will be the absorption power of different materials. Into the water window regime, this specific energy range, defined from the carbon K-absorption edge at 282 eV to the oxygen K-edge at 533 eV, makes water relatively transparent but keeps carbon absorption power, making cell components visible,^398^ together with any heavier inorganic particle allowing 3D single NP visualization.^374,399^

If there is the need to work above the water window, as would be the case for thicker samples, the absorption power of both water and cell components for harder X-rays is very similar, thus making individual cellular component imaging impractical despite individual particles still being visible. Nonetheless, similar to an optical microscope, a lens-based X-ray microscope can be equipped with a Zernike phase plate for phase contrast of weakly absorbing structures^400,401^

**Fig. 19:**
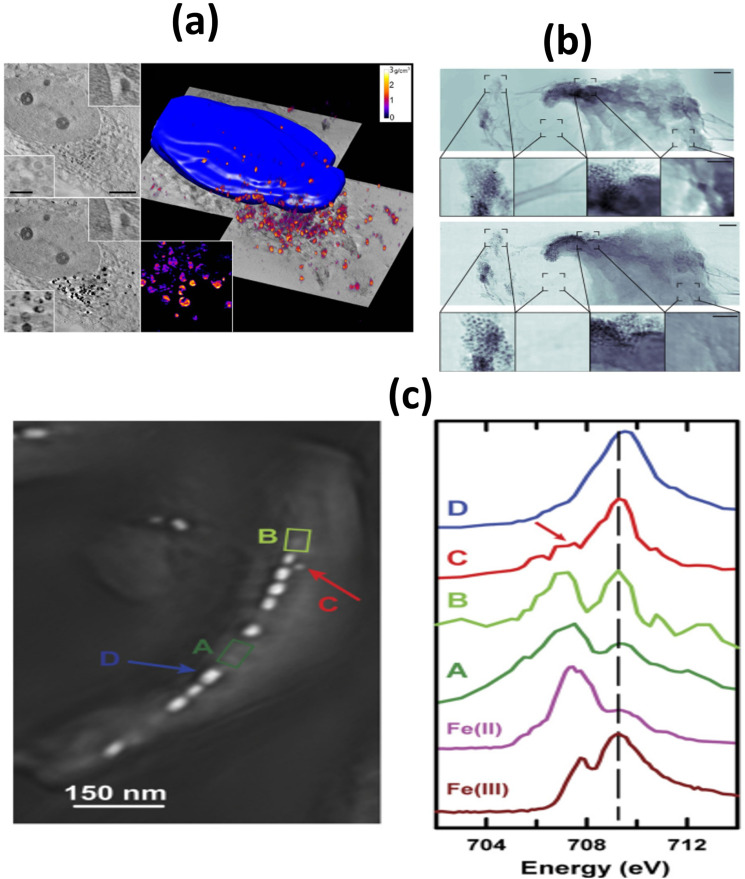
Examples of XRM used for the study of the Fe content on biological systems. (a) Iron oxide nanoparticle localisation inside a mammalian cell by full-field X-ray microscopy. Reproduced from ref. 374 under a Creative Commons (CC BY) license from Springer Nature, Copyright 2016. (b) Individual core–shell nanoparticle of silica@iron oxide localised on a mammalian cell by ptychography. Reproduced from ref. 413 under a Creative Commons (CC BY) license from Springer Nature, Copyright 2017. (c) Chemical characterisation by X-ray spectroscopy ptychography of individual magnetosomes inside bactéria. Reproduced from ref. 376 under a Creative Commons (CC BY) license from PNAS, Copyright 2016.

More recently, to avoid the high-energy X-ray optics limitations, a new group of lensless XRM, known as coherent diffraction imaging (CDI), was developed. In general, CDI reconstructs the amplitude and phase modulation of a coherent X-ray illumination scattered by a sample.^402^ The sample-modulated X-ray propagates and eventually creates a diffraction pattern to be recorded by an area detector. From this diffraction pattern, the sample image is reconstructed by using phase retrieval algorithms.^372,403^ Thus, the detector, together with the phase retrieval algorithms, replaces the objective lenses, and, consequently, CDI techniques are known as lensless microscopy, implying the manufacturing and quality of the X-ray objective lens has no impact on the technique. Several CDI methods have been developed, including plane-wave CDI, Bragg CDI, X-ray holography, ptychography, and others.^403,404^ The distinction between those resides in the experimental setup and the mathematical modelling used by the phase retrieval algorithm. Among those methods, examples regarding this work have mostly been performed using ptychography (Fig. 18d) ranging from the 2D imaging of cells,^405–407^ 3D ptycho-tomography of cells,^402,408^ tissues^409–412^ and biostructures.^411,412^ Finally, ptychography can be combined with X-ray absorption spectroscopy allowing for a specific observation of IONPs,^372,413^ or be combined with other scanning X-ray microscopy techniques like SXFM^414,415^ for elemental specify contrast, carrying a big promise for single particle biotransformation imaging and characterisation into cells. Main examples of the discussed XRM used for the study of Fe content on biological systems are presented in Fig. 19.

#### 3.5.6. Immunohistochemical techniques

Several studies have emphasized the importance of immunochemistry (IHC) in understanding the interactions and effects of NPs in various medical scenarios, for example, the clinical study by Grauer *et al.*^416^ An avidin-biotin peroxidase technique immunohistochemistry with a Dako REAL™ Detection System (K5001) staining demonstrated the upregulation of caspase-3 and heat shock protein 70 and the infiltration of macrophages and T-cells in glioblastoma patients treated with amino silane-coated MIONPs (magnetite core of ∼12 nm), cluster of differentiation 3-positive (CD3^+^) T-cells, and radiotherapy. Additionally, flow cytometry revealed higher IFN-γ to IL-4 ratios in CD4^+^ and CD8^+^ memory T cells, and activation of tumor-associated myeloid cells and microglia, indicated by upregulated HLA-DR and PD-L1. Notably, two patients experienced long-lasting treatment responses for over 23 months without additional therapy. In this study, IHC was crucial in identifying and visualizing specific proteins and cell types within the tissue samples. The avidin-biotin peroxidase technique was used to detect various markers, such as CD3 (T-cells), CD8 (cytotoxic T-cells), CD68 (macrophages), myeloperoxidase (MPO), heat shock protein 70 (HSP70), and caspase-3, in glioblastoma tissues treated with NPs and radiotherapy. The staining allowed for the precise localization and quantification of these proteins, helping to reveal the upregulation of caspase-3 and HSP70 and the infiltration of immune cells like T-cells and macrophages in response to the treatment. Thus, IHC was essential for demonstrating the cellular responses and interactions within the treated tissues. This is just one study that highlights the significance of IHC in revealing cellular responses to NP treatments within specific tissues. Table 4 provides a structured overview of the critical steps and considerations in performing IHC techniques for studying MIONPs.

**Table 4:** Summary of the common IHC technique steps for MIONPs

No.	Step	Description	Necessity
1	Sample preparation	Fixation in formalin, embedding in paraffin or OCT compound, sectioning into thin slices (4–10 μm)	Essential; proper fixation, embedding, and sectioning are crucial for preserving tissue morphology and enabling accurate staining
2	Deparaffinization and rehydration	For paraffin sections: deparaffinize in xylene, rehydrate through graded alcohols (100% to 70%), wash in water	Essential for paraffin-embedded sections; necessary to remove paraffin and rehydrate the tissue for subsequent staining
3	Antigen retrieval	Use heat-induced epitope retrieval (HIER) or enzymatic retrieval methods to unmask antigens, with buffers like citrate or EDTA	Generally essential; antigen retrieval is typically necessary to unmask epitopes that may be hidden during fixation and embedding; some antigens may not require retrieval so that this step can be adjusted
4	Blocking non-specific binding	Incubate sections with blocking buffers (normal serum or BSA) to prevent non-specific binding of antibodies	Essential; blocking is crucial to avoid non-specific binding of antibodies, which can lead to high background staining
5	Primary antibody incubation	Incubate sections with primary antibodies specific to the target antigen. For MIONPs, primary antibodies could target markers of cells (*e.g.*, macrophages, dendritic cells) or MIONP-associated proteins	Essential; the primary antibody is necessary for specific binding to the target antigen
6	Detection of MIONPs	Prussian blue staining: forms a blue color with ferric ions to visualize Fe deposits	Essential but method dependent
		Fluorescently labeled antibodies: direct visualization using fluorescence microscopy	
7	Secondary antibody incubation	Incubate sections with secondary antibodies conjugated to enzymes (HRP or AP) or fluorophores, binding to the primary antibody	Essential for indirect detection methods; necessary if using primary antibodies that are not directly labeled; if using directly labeled primary antibodies, this step can be skipped
8	Signal detection	Chromogenic detection: enzyme-conjugated secondary antibodies with substrates like 3,3′-diaminobenzidine (DAB) for colored precipitate	Essential; detection methods are necessary to visualize the bound antibodies
		Fluorescence detection using fluorescence microscopy	
9	Counterstaining	Use hematoxylin (chromogenic IHC) or DAPI (fluorescence IHC) to visualize cell nuclei and tissue architecture	Optional but recommended; counterstaining helps to visualize the overall tissue architecture and cell nuclei, providing context to the specific staining
10	Mounting and imaging	Mount sections with appropriate media, cover with coverslips, and image using light or fluorescence microscopy	Essential; proper mounting and imaging are necessary to preserve the stained sections and to visualize and analyze the results accurately

It is worth noting that certain steps in the IHC process can be skipped or adjusted depending on the specific experimental requirements and the type of tissue being studied. Antigen retrieval, for instance, can be modified or sometimes omitted based on the nature of the antigen and the tissue type, as not all antigens require this step. The detection of MIONPs is method-dependent; if a method that does not involve Prussian Blue or direct detection is not needed, this step can be adjusted accordingly. Similarly, secondary antibody incubation can be skipped if directly labeled primary antibodies are used. Counterstaining is optional but recommended to provide additional context to the staining by visualizing overall tissue architecture and cell nuclei. Lastly, deparaffinization and rehydration are essential for paraffin-embedded sections but are not required for frozen sections. By understanding which steps are flexible, researchers can tailor their IHC protocols to best suit their study on MIONPs. Formalin fixation, involving formaldehyde, is widely used as a sample preparation to preserve tissues by quickly permeating cell walls and membranes and maintaining cell structure.^417^ Deparaffinization, a critical step in IHC, involves removing paraffin wax from tissue sections to enable proper antigen retrieval and staining. This process conventionally relies on solvents like xylene for paraffin removal. For example, Sharma *et al.*^418^ following deparaffinization, tissue sections undergo rehydration through a series of graded alcohols, transitioning from 100% alcohol to lower concentrations like 70%, culminating in a final wash in water to prepare them for subsequent staining procedures.^419^ Antigen retrieval is a crucial step in IHC to restore the immunoreactivity of fixed antigens, including those related to MIONPs.^420^Table 5 presents various antigen retrieval techniques in immunohistochemistry.

**Table 5:** Various antigen retrieval techniques in immunohistochemistry

Technique	Description	Common buffers/enzymes	Method	Reference
Heat-induced epitope retrieval (HIER)	Heating tissues in a buffered solution to break cross-links and unmask epitopes	Citrate buffer (pH 6.0); EDTA buffer (pH 8.0–9.0); Tris–EDTA buffer (pH 9.0)	Microwave: 10–20 min; pressure cooker: consistent high-temperature heating; water bath or steamer: slower heating	Krenacs *et al.*,^421^ Vinod *et al.*^422^
Enzymatic antigen retrieval	Proteolytic enzymes digest proteins masking epitopes	Trypsin; proteinase K; pepsin; pronase	Incubate tissue in enzyme solution at 37 °C for 10–30 min	McNicol *et al.*,^423^ Janardhan *et al.*^424^
Combined heat and enzymatic retrieval	Combines HIER and enzymatic digestion for difficult antigens	Based on specific HIER and enzyme used	Apply HIER first, followed by enzymatic digestion	Kouchmeshky *et al.*,^425^ Renshaw,^426^ Krenacs *et al.*,^427^ Janardhan *et al.*^424^
pH adjustment	Adjusting buffer pH to optimize antigen retrieval	Acidic buffers (pH 6.0); basic buffers (pH 8.0–9.0)	Modify pH of buffer according to the specific antigen requirements	Krenacs *et al.*,^421^ Shi *et al.*,^428^ Emoto *et al.*^429^
Microwave-assisted retrieval	Uses microwave for rapid and consistent heating during HIER	Citrate, EDTA, or Tris–EDTA buffers	Heat slides in microwave at full power, then cool at room temperature	Temel *et al.*,^430^ Katoh^431^
Pressure cooker method	Uses consistent high-temperature heating in a pressure cooker (*e.g.*, Instant Pot®) for HIER	Citrate, EDTA, or Tris–EDTA buffers	Heat slides in retrieval buffer under pressure for 10–20 min; may take up to 40 min using household pressure cooker	Krenacs *et al.*,^421^ Kearns *et al.*^432^
Autoclave retrieval	Applies high pressure and temperature using an autoclave	Citrate, EDTA, or Tris–EDTA buffers	Autoclave at 121 °C for 10–15 min	Ehara *et al.*^433^
Ultrasonic retrieval	Uses ultrasonic energy to assist antigen retrieval; may not be generally applicable or may not significantly improve retrieval yield as compared to traditional methods	Citrate, EDTA, or Tris–EDTA buffers	Immerse tissue in retrieval solution within an ultrasonic bath	Hatta *et al.*^434^

Various methods are employed to prevent non-specific bindings in IHC involving MIONPs and avoid unwanted interactions that could lead to false results. One common approach is blocking agents such as serum or bovine serum albumin (BSA) to saturate non-specific binding sites on the tissue sections. Amirian *et al.*^435^ utilized serum (Gibco™) and Triton™ X-100 in PBS to block non-specific binding in their immunofluorescence labeling studies, demonstrating the effectiveness of this approach in reducing background staining and enhancing specific antibody binding. By pre-incubating tissue sections with blocking agents, researchers can minimize non-specific interactions and improve the specificity of IHC staining, including when working with MIONPs. In addition to blocking agents, specific blocking buffers tailored to the experimental setup can help reduce non-specific binding. Kristensen *et al.*^436^ employed commercial True-Stain Monocyte Blocker and phosphorothioate-oligodeoxynucleotides (referred to as “Oligo-Block”) to remove non-specific binding without affecting the specific binding of antibodies, highlighting the importance of selecting appropriate blocking reagents for particular applications, including those involving MIONPs. These specialized blocking buffers can effectively reduce background staining while preserving the specific binding of antibodies to their target antigens, ensuring accurate detection of MIONPs.

Furthermore, optimizing blocking conditions, including incubation time and temperature, can significantly impact the success of IHC experiments using MIONPs. Brasino *et al.*^437^ developed a method to turn antibodies off and on using a covalently tethered blocking peptide, demonstrating a precise approach to selectively block antibody binding pockets without modifying the primary antibody sequence. In their study, the covalent bond secured the developed linker and blocking peptide to the antibody's light chain, ensuring the blocking peptide remained near the antibody's binding site. This positioning effectively placed the antibody in an “off-state” (incapability of binding to a target). Incorporating protease-cleavable and photocleavable elements in the tether allowed for controlled activation of the antibody to its “on-state” for both anti-FLAG (Asp–Tyr–Lys–Asp–Asp–Asp–Asp–Lys) and cetuximab antibodies. By fine-tuning the blocking conditions, researchers can enhance the specificity of antibody–antigen interactions and minimize non-specific bindings in IHC involving MIONPs. Moreover, blocking reagents and conditions should be tailored to the specific experimental requirements and target antigens. Non-specific bindings can effectively be reduced, and the accuracy of IHC analyses can be improved by customizing the blocking strategy to the characteristics of the target antigens and tissues, including those with MIONPs.

In detecting MIONPs in IHC, choosing primary antibodies is crucial to ensure accurate and specific detection. Several studies provide insights into using primary antibodies for incubation in detecting MIONPs. For instance, Zhao *et al.*^438^ demonstrated the activation of immune cells and cytokine production induced by MIONPs, highlighting the potential of these NPs in promoting immune responses. This study underscores the importance of selecting primary antibodies that effectively target and detect MIONPs in IHC assays. Moreover, Gao *et al.*^439^ described a strategy using platelets as carriers for co-delivering anti-PDL1 (programmed death-ligand 1) antibodies and MIONPs to the postsurgical tumor site for cancer immunotherapy. Similarly, Lee *et al.*^440^ conjugated lipid-coated superparamagnetic MIONPs with PD-L1 antibodies (25.8 ± 1.8 nm hydrodiameter) for the identification of PD-L1 expression in glioblastoma, highlighting the importance of antibody–NP conjugates in molecular imaging and targeted therapy. Here, PD-L1-SPIO demonstrated a specific binding affinity to PD-L1 in the mouse glioblastoma cell line (GL261). The presence and amount of PD-L1-SPIO in temozolomide-resistant glioblastoma cells and tumor tissue were validated using Prussian blue staining and *in vivo*
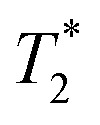
 map MRI, respectively. This research demonstrates the utility of primary antibodies in detecting specific biomarkers associated with MIONPs in disease diagnosis and treatment. The selection of appropriate primary antibodies is essential for accurately detecting and characterizing MIONPs in IHC. Studies such as those by Zhao *et al.*,^438^ Gao *et al.*,^439^ and Lee *et al.*^440^ provide valuable insights into using primary antibodies for targeting and detecting MIONPs, showcasing their potential in various biomedical applications. A summarized table of primary antibodies for detecting MIONPs is presented in Table 6. Using these primary antibodies, researchers can effectively identify and analyze MIONPs' presence, distribution, and effects in various biological samples. The choice of antibody depends on the specific characteristics and coatings of the NPs used in the study.

**Table 6:** List of example common antibodies used for detecting MIONPs

Primary antibody	Target	Use	Reference
Anti-ferritin	Ferritin	Detecting iron accumulation and distribution in tissues	Fernández *et al.*,^441^ Horvat *et al.*^442^
Anti-transferrin	Transferrin	Studying uptake and distribution of MIONPs	Huang *et al.*^443^
Anti-dextran	Dextran (if coated)	Detecting dextran-coated MIONPs	Mohammed *et al.*^444^
Anti-polyethylene glycol (PEG)	Polyethylene glycol (if PEGylated); anti-PEG (if anti-PEGylated)	Detecting PEG/antiPEG-coated MIONPs	Chen *et al.*,^445^ Liu *et al.*,^446^ Hsieh *et al.*^447^

Developing antibodies that directly bind to the surface of MIONPs is challenging due to several factors. Firstly, antibodies typically recognize specific protein or carbohydrate structures, known as epitopes, on antigens. However, MIONPs are inorganic and composed of iron oxide, which lacks the complex biological structures that antibodies usually target. The surface of MIONPs does not naturally present the proteinaceous or polysaccharide features necessary for antibody binding. MIONPs are often coated with materials like polymers or organic molecules to stabilize the particles and enhance their biocompatibility. While these coatings can be functionalized to attach biomolecules, including antibodies, the iron oxide core remains an unsuitable target for direct antibody binding due to its lack of specific epitopes. Instead of developing antibodies that bind directly to MIONPs, researchers typically functionalize the surface of these NPs with specific biomolecules, such as peptides or antibodies, that can target particular cells or tissues. This approach allows MIONPs to be directed toward specific biological targets without needing the NPs themselves to be recognized by antibodies. Moreover, even if antibodies could recognize and bind to MIONPs, such interactions might be non-specific and weak, leading to potential issues in biological applications, such as unwanted binding to non-target sites. Therefore, the inorganic nature of MIONPs and lack of specific biological epitopes make them unsuitable targets for direct antibody binding, prompting functionalization strategies to achieve targeted interactions. After incubation of a primary antibody, detection of MIONPs may follow, depending on the protocol. After that, secondary antibodies bind to the primary antibodies and facilitate visualization. These secondary antibodies are typically conjugated to enzymes (like horseradish peroxidase, HRP) or fluorescent dyes (like Alexa Fluor® or FITC) to allow for detection through colorimetric or fluorescent methods. A list of commonly used secondary antibodies for this purpose is presented in Table 7.

**Table 7:** Commonly used secondary antibodies for detecting MIONPs

Secondary antibody type	Example	Use	Reference
HRP-conjugated	Goat anti-rabbit IgG (HRP), goat anti-mouse IgG (HRP)	Colorimetric detection with substrates like 3,3′-diaminobenzidine (DAB)	Wang *et al.*^448^
Fluorescent dye-conjugated	Goat anti-rabbit IgG (Alexa Fluor® 488), donkey anti-mouse IgG (FITC)	Fluorescent detection under a fluorescence microscope	Sumner & Kopelman^449^
Biotinylated	Goat anti-rabbit IgG (biotin), goat anti-mouse IgG (biotin)	Used with avidin–biotin complex (ABC) methods for amplified detection	Chen *et al.*^450^
Alkaline phosphatase (AP)-conjugated	Goat anti-rabbit IgG (AP), Goat anti-mouse IgG (AP)	Colorimetric detection with substrates like BCIP/NBT	Predoi *et al.*^451^

To ensure optimal binding and detection of MIONPs in IHC, specific protocols are followed for incubating secondary antibodies. Yang *et al.*^452^ demonstrated a method where tissue sections were incubated with a secondary antibody linked to horseradish peroxidase (HRP) at 37 °C for 40 min after primary antibody incubation, followed by a reaction with a substrate solution. This sequential incubation process allows the secondary antibody to bind to the primary antibody, facilitating the visualization of MIONPs through enzymatic reactions. In a study by Shahhosseini *et al.*,^453^ the membrane was incubated with a secondary antibody for 1 h at room temperature following primary antibody incubation, enabling the detection of MIONPs in the sample. This incubation step with the secondary antibody is crucial for amplifying the signal generated by the primary antibody, leading to the visualization of MIONPs in the tissue section. Moreover, Schoenfeld *et al.*^454^ described a multistaining protocol involving sequential incubation with primary and secondary antibodies for IHC and immunofluorescence imaging. This protocol allows for the detection of MIONPs along with other antigens in the same tissue section by incubating with different primary and secondary antibodies sequentially, enhancing the information obtained from the sample. Bassiouni *et al.*^455^ developed a double-blocking protocol for immunofluorescent labeling of murine cochlear sections with primary mouse antibodies, involving incubation with primary antibodies followed by secondary antibodies. This method effectively addresses the mouse-on-mouse problem in immunofluorescence by utilizing a double-blocking strategy during the incubation steps, which can be adapted to ensure the specific and accurate detection of MIONPs.

As secondary antibodies have been linked, Table 8 provides a range of options for detecting MIONPs in IHC, each with unique benefits to suit different experimental needs. Various methods are employed to detect MIONPs in IHC, each offering distinct advantages tailored to specific experimental requirements. Enzymatic colorimetric detection utilizes horseradish peroxidase (HRP) or alkaline phosphatase (AP) to catalyze chromogenic substrates, making a color change visible under a microscope. HRP plays a crucial role in chemiluminescent reactions, where it catalyzes the oxidation of luminol by hydrogen peroxide to produce luminescent signals for detection purposes.^456^ Recent studies have focused on enhancing the sensitivity and accuracy of chemiluminescent assays by incorporating HRP into innovative platforms, such as chemiluminescent lateral flow immunoassays (LFIA).^457^ These LFIA systems have been developed using Au NP–antibody–HRP–polyethylene glycol conjugates for detecting biomarkers like cardiac troponin I, showcasing the potential of HRP-based chemiluminescence in clinical diagnostics.^457^ However, it would be of great interest for these chemiluminescent systems to be incorporated into MIONP detections. Integrating functional nanomaterials with chemiluminescence detection methods has shown promise in improving detection performance.^458^ For instance, the combination of Pb^2+^-dependent DNAzyme and hemin/G-quadruplex motifs that mimic HRP has been utilized for rapid and portable detection of metal ions through chemiluminescence resonance energy transfer, demonstrating the potential of nanomaterial-based systems (for example, in MIONP-based IHC) in sensitive detection applications.^458^

**Table 8:** Overview of detection methods for MIONPs in IHC

Detection method	Description
Enzymatic colorimetric detection	Uses HRP or AP to catalyze chromogenic substrates to produce a color change
Fluorescence detection	Employs fluorescent dye-conjugated secondary antibodies for visualization
Biotin–avidin detection system	Uses biotinylated secondary antibodies and avidin/streptavidin conjugates for signal amplification
Enhanced chemiluminescence (ECL)	Uses chemiluminescent substrates catalyzed by HRP or AP, emitting light detected by a camera
NP-based detection	Utilizes NP-conjugated antibodies for enhanced signal amplification and high-resolution imaging
Tyramide signal amplification (TSA)	HRP-conjugated secondary antibodies catalyze the deposition of labeled tyramide for amplification

Fluorescence detection also relies on fluorescent dye-conjugated secondary antibodies for visualization, enabling the detection of multiple targets simultaneously with high specificity. The biotin–avidin detection system utilizes biotinylated secondary antibodies and avidin/streptavidin conjugates to amplify the signal, enhancing detection sensitivity. Biotin, known for its high affinity for streptavidin-type membrane proteins, is a targeting ligand for avidin-class proteins, enabling precise and efficient targeting at the cell surface.^459^ The strong association between biotin and avidin, ranging from 10^13^–10^15^ M^−1^, forms the basis for the robust and specific binding interactions utilized in detection systems.^460^ In the context of drug delivery systems, integrating biotin–avidin interactions with MIONPs has shown promise in targeted therapies. For instance, the preparation of peptide and recombinant tissue plasminogen activator conjugated poly(lactic-*co*-glycolic acid) magnetic NPs involved binding PMNP-avidin with biotin-PEG-rtPA, demonstrating the versatility of the biotin–avidin system in dual-targeted thrombolytic therapy.^461^ This approach highlights the potential of utilizing MIONPs with the biotin–avidin system for precise and targeted drug delivery applications. In developing immunochromatographic test strips with enhanced sensitivity, functionalization of nanocomposite probes with biotinylated proteins has been achieved to modify probe surfaces with desired biotinylated antibodies through biotin–avidin binding.^462^ This strategy demonstrates the utility of the biotin–avidin system in enhancing the sensitivity and specificity of detection platforms, showcasing the potential for MIONPs in such applications.

Tyramide signal amplification (TSA) involves the deposition of labeled tyramide catalyzed by HRP-conjugated secondary antibodies, enabling high sensitivity detection of MIONPs. Also known as catalyzed reporter deposition (CARD), this is an enzyme-mediated amplification method used to enhance the sensitivity of various assays, particularly in fixed-cell assays like immunocytochemistry and *in situ* hybridization.^463^ TSA utilizes enzymatic reactions to amplify signals, enabling the detection of low-abundance targets in histochemical analysis. The application of TSA has shown promise beyond traditional assays, suggesting its potential applicability in novel areas such as detecting targets in conjunction with MIONPs. By combining TSA with MIONPs, researchers can develop more effective methods for detecting targets in histochemical analysis, taking advantage of TSA's sensitivity and MIONPs' unique properties. Additionally, combining TSA with multiple immunolabeling techniques could support translational oncology studies involving MIONPs, allowing for the high-sensitivity detection of various targets in complex biological samples.^464,465^ Further exploring TSA with MIONPs could open new avenues for sensitive and specific detection methods in biomedical research and diagnostic applications.

Moreover, several instruments are commonly employed to facilitate the visualization and analysis of signals. Microscopes are essential for observing the colorimetric or fluorescent signals the detection methods generate. Specifically, fluorescence microscopes are utilized to visualize the fluorescent signals emitted by fluorophore-conjugated secondary antibodies in fluorescence detection. Imaging systems, including cameras or imaging systems, are essential for capturing and documenting the signals produced by enhanced chemiluminescence (ECL) detection, enabling quantitative analysis of the results. Additionally, electron microscopes are utilized for NP-based detection to visualize NP-conjugated antibodies and achieve high-resolution imaging. These instruments play crucial roles in IHC experiments, enabling researchers to detect and visualize MIONPs in tissue samples using various detection methods.

Research trends in IHC involving magnetic NPs have shown significant advancements in recent years. Magnetic NPs, such as SPIONs, have been extensively researched for their potential to enhance MRI data, tissue engineering, drug delivery, cancer diagnosis, and therapy.^466,467^ The development of a new IHC method using fluorescence-emitting phosphor-integrated dot NPs has been recently introduced to detect cancer cell proteins accurately and may be integrated into the IHC of MIONPs.^468^ The future direction of research in this field may involve further exploration of magnetic NP applications in targeted drug delivery, responsive drug release systems, and precise magnetic therapy *in vivo*.^469^ Enhancing the magnetic characteristics of NPs, investigating surface modifications, and optimizing drug delivery systems for treating various diseases, including bone diseases and melanoma, may see continued development.^470^ Interestingly, the discovery of magneto-sensing proteins and magneto-sensing-protein-expressing cells is an emerging field, which may lead to an antibody–antigen-free detection, and consequently cell/tissue imaging, of MIOPs.^471^ Advances in these areas could lead to more effective and safer NP-based therapies and novel diagnostic tools. The integration of MIONPs in IHC and other biomedical techniques is a growing focus, with the potential to provide deeper insights into disease mechanisms and improve the precision of treatments. As the field progresses, interdisciplinary collaboration between materials science, biology, and medicine will be essential to fully realize the potential of MIONPs in clinical settings.

## 4. Conclusion, challenges, and future perspectives

The route of entry for MIONPs plays a critical role in determining their biodistribution, cellular interactions, and potential effects within the body. While inhalation, oral ingestion, dermal exposure, and injection are all viable routes, each has distinct implications for NP behavior and potential toxicity. Inhalation poses concerns for respiratory health, with ongoing research exploring its potential for drug delivery and treatment of respiratory diseases. Oral ingestion and dermal exposure highlight the importance of NP size, surface properties, and formulation in minimizing toxicity and enhancing therapeutic outcomes. Injection, particularly intravenous administration, remains the most established route for MIONPs, with FDA-approved formulations primarily used in MRI imaging and the treatment of iron deficiency anemia. The choice of administration route influences the systemic distribution, targeting specificity, and clearance of MIONPs, impacting their effectiveness in biomedical applications such as drug delivery, imaging, and cancer therapy. As the field continues to advance, a better understanding of these routes and their effects will be critical to optimizing MIONPs for safe and effective clinical use.

MIONPs hold great promise in biomedical research and clinical applications due to their versatility in cellular uptake mechanisms and their potential for targeted drug delivery, imaging, and therapy. The surface chemistry of MIONPs plays a critical role in determining the pathways of cellular internalization, which include receptor-mediated endocytosis, fluid-phase endocytosis, phagocytosis, and magnetic force-mediated internalization. By modifying the surface properties of MIONPs, such as conjugating specific ligands or antibodies targeting receptors like transferrin, EGFR, folate, PSMA, integrins, or CD44, researchers can enhance their specificity and efficiency in entering cells, leading to more precise delivery of therapeutic agents. Receptor-mediated endocytosis, particularly for receptors highly expressed in cancer cells, offers selective targeting capabilities, allowing for the controlled introduction of NPs into specific cell types based on receptor expression. Fluid-phase endocytosis, on the other hand, facilitates nonspecific uptake, broadening the range of target cells. Phagocytosis by immune cells provides a unique approach to immunotherapy and the modulation of immune responses, while magnetic force-mediated internalization offers precise control over NP localization within target cells. The mechanisms governing MIONP uptake underscore the importance of surface modifications in optimizing their interactions with cells. By harnessing these pathways, MIONPs can be strategically designed to enhance therapeutic efficacy, improve imaging precision, and minimize off-target effects, paving the way for innovative applications in personalized medicine and cancer treatment.

MIONPs also exhibit complex interactions with biological systems, primarily through mechanisms of cellular uptake, intracellular localization, and oxidative stress. Their internalization into cells is driven by endocytosis, receptor-mediated pathways, and other cellular processes such as macropinocytosis and phagocytosis, influenced by factors like particle size, surface charge, and coating materials. Once inside cells, MIONPs can trigger oxidative stress by generating ROS, leading to potential cytotoxicity, DNA damage, and apoptosis. This ROS production is a crucial feature of MIONPs, making them effective in applications such as cancer treatment and bacterial eradication, though it also poses risks of genotoxicity and inflammation. Additionally, the balance between pro-apoptotic and anti-apoptotic proteins, particularly in the BCL-2 family, plays a significant role in determining cellular fate in response to MIONP exposure, especially through the modulation of the mitochondrial apoptotic pathway. MIONPs have been shown to induce oxidative stress, genotoxicity, and apoptosis in a dose-dependent manner, with NP properties such as size, surface coating, and concentration playing critical roles in determining their biological impact. The surface modification of MIONPs, particularly with coatings like PEI or PEG, can influence their cytotoxicity, cellular uptake, and biodistribution. These factors are essential for optimizing MIONPs in biomedical applications, such as drug delivery, imaging, and cancer therapy, while minimizing potential adverse effects. Understanding the intricate interactions between MIONPs and cellular systems is crucial for designing safer and more effective NP-based therapies. Further research is needed to clarify the long-term effects and optimize the use of MIONPs for therapeutic applications, ensuring their benefits outweigh potential risks.

The accumulation of MIONs within cells is a complex process governed by multiple factors, including NP properties, cellular uptake mechanisms, and external influences such as magnetic fields. MIONs are primarily internalized through endocytosis, phagocytosis, and receptor-mediated pathways, with their accumulation in various organelles, such as lysosomes, mitochondria, and the cytoplasm, influencing cellular functions. External magnetic fields, particularly pulsed fields, enhance NP uptake, providing a controlled method for promoting accumulation in target cells. Surface functionalization with ligands or polymers allows for further tuning of their interactions with cells, balancing biocompatibility with specific targeting capabilities. The biodistribution of MIONs *in vivo* depends on NP size, charge, and coating, affecting their accumulation in organs such as the liver, spleen, and brain. NPs can undergo biotransformation and speciation changes within cells, leading to the release of iron ions or retention as metal oxides. These factors are critical for designing MIONs for therapeutic applications like drug delivery, imaging, and cancer treatment. Understanding the intracellular fate and organ-specific distribution of MIONs is essential for optimizing their biomedical use and ensuring their safety and efficacy.

IHC is an indispensable tool for investigating the biological interactions, cellular responses, and therapeutic effects of NPs, particularly MIONPs. As demonstrated in various studies, including those focusing on glioblastoma, lung tissue, and cancer immunotherapy, IHC allows for the precise localization and visualization of specific proteins, immune cells, and NP-associated markers within tissues. Key steps in IHC protocols, such as sample preparation, antigen retrieval, blocking of non-specific binding, and primary antibody incubation, are crucial for the accurate detection of MIONPs and their effects on biological systems. The flexibility in adjusting certain IHC steps, such as deparaffinization and antigen retrieval, depending on tissue type and experimental goals, further enhances the technique's applicability. Moreover, the choice of primary antibodies plays a pivotal role in detecting MIONPs, especially in the context of their conjugation with therapeutic molecules or immune markers like PD-L1. Studies using innovative IHC techniques, including Prussian blue staining for NP detection and fluorescence-based methods, demonstrate the capacity of IHC to provide insights into NP localization, cellular uptake, and their impact on immune responses. Thus, IHC remains a critical methodology in the study of NP-based therapies, facilitating breakthroughs in understanding NP interactions within complex biological environments and advancing the development of NP-driven diagnostics and therapeutics.

It is important to note that targeting efficiency of nanoparticles *in vivo* often remains low.^472,473^ Overall, targeting efficiency is relatively comparable across different targeting strategies, with the percentage of injected MIONPs reaching the tumor ranging from 0.0051% to 3% for passive targeting, 0.1% to 7% for molecular targeting, and 0.0051% to 2.6% for magnetic targeting.^474^ For MIONPs specifically, this limitation arises from several factors. While targeting efficiency close to 100% is the theoretical ideal, experimental data suggests that efficiencies greater than 5% are rarely achieved for magnetic nanoparticles under *in vivo* conditions. The targeting efficiency of Fe_3_O_4_ nanoparticles under magnetic field guidance is demonstrated by their enhanced accumulation in tumors, with larger nanoparticles showing significantly higher retention compared to smaller ones. For example, in the study of Guo and colleagues, Fe_3_O_4_ nanoparticles of 310 nm in size exhibited the highest tumor accumulation, with a 1.94-fold increase in the presence of an MF, achieving 29.5 injected dose (ID)%/g in the tumor under magnetic field *versus* 15.3 ID%/g in the tumor without MF at 48 h post-injection.^475^ On the other hand, Shen and colleagues^476^ studied the targeting efficiency of DOX@ES-MION3@RGD2@mPEG3 nanoparticles. The higher accumulation in tumors compared to the liver and spleen was evident from the ΔSNR (signal-to-noise ratio) values obtained from *in vivo T*_1_-weighted MR imaging, with the tumor showing the highest ΔSNR (173.8 ± 17%) in contrast to the liver (81.9 ± 28%) and spleen (78.2 ± 30%). Furthermore, elevated iron levels in tumors post-injection, reinforcing their strong tumor-targeting ability. According to the authors, the efficiency is attributed to the nanoparticles' optimized hydrodynamic diameter (13.1 nm), which ensures effective tumor penetration and retention, along with their precise targeting facilitated by RGD peptide ligands. The differences in nanoparticle size between the two studies reflect their focus on distinct targeting mechanisms: magnetic field guidance *versus* molecular targeting. While Guo *et al.*^475^ prioritized magnetic responsiveness with larger nanoparticles, Shen *et al.*^476^ emphasized tumor penetration and ligand-mediated specificity with smaller nanoparticles. Combining these approaches, such as optimizing nanoparticle size to balance magnetic responsiveness with tumor penetration, could potentially achieve superior targeting efficiencies. The following are the primary limitations affecting the targeting efficiency of MIONs:

• Physicochemical properties of MIONPs: size distribution, nanocrystalline shape, and their arrangement patterns influence biodistribution, cellular uptake, and magnetic behavior.^477^ Consistent size and shape improve circulation time and minimize immune clearance, while specific arrangement patterns enhance magnetic alignment and targeting accuracy when guided by external magnetic fields.

• Non-specific biodistribution: after intravenous injection, MIONPs are rapidly cleared by the mononuclear phagocyte system, primarily through the reticuloendothelial system in organs such as the liver and spleen, which significantly reduces the availability of nanoparticles for active targeting.^281^

• Physiological barriers: barriers such as blood flow dynamics, endothelial permeability, and the tumor microenvironment (for cancer targeting) reduce the efficiency of nanoparticle delivery.^478–481^

• Magnetic field strength and gradient: external magnetic fields often lack sufficient depth penetration and strength to effectively guide MIONs to targets in deep tissues.^482^

• Protein corona formation: interaction of MIONs with plasma proteins can alter their surface properties and reduce their ability to recognize molecular targets.^483,484^

• Off-target accumulation: non-specific interactions with non-target tissues and cells may further dilute the targeting efficiency.

Over the years, efforts have been made to improve targeting efficiency through advancements in surface functionalization, improved magnetic field designs, and enhanced nanoparticle formulations.^473,476^ In SPION-based delivery nanosystems, various targeting ligands such as transferrin, antibodies, aptamers, hyaluronic acid, folate, and targeting peptides are incorporated onto the surface of SPIONs to enhance the drug targeting efficiency.^485^ Despite these improvements, achieving efficiencies beyond this targeting efficiency range remains challenging.

The creation of 3D tumor models has transformed the study of cancer and endorsed its use in thermal therapeutics applications. These models are foreseen to provide a better understanding of the interactions between cancer cells and hyperthermic nanomedicines, as well as the control of the tumor microenvironment (TME) on drug delivery and nanomedicines dispersion inside the tumor, as well as the reaction to treatment. Researchers can improve treatment plans and create more therapeutically applicable hyperthermia cancer therapy techniques if they have a thorough understanding of these pathways. 3D models are more resistant to the therapy, according to comparative research comparing the effectiveness of hyperthermic nanomedicines in tumor spheroids and monolayers. Researchers have been concentrating on creating various 3D tumor models in an effort to improve upon traditional 2D monolayered models. Spheroids, which are made up of spheroidal, randomly self-assembled cell-rich formations, are the most often used 3D *in vitro* models.^486,487^ These models may consist of multiple cell types (heterotypic) or only one kind (monotypic).^488^ Heterotypic spheroids have been used to screen many kinds of potential treatments and can be bioengineered with adjustable cell density and size.^489^ Heterotypic spheroids are thought to be more clinically relevant models since they more closely resemble the cellular complexity of genuine malignancies. And used to evaluate drug and nanoparticle penetration, chemotherapeutic response, and the impact of various cell types on tumor growth and gene and protein expression patterns. The differences in readouts between preclinical and clinical trials for screening advanced anti-cancer medicines should be lessened by combining developments in 3D *in vitro* tumor models with new methods for hyperthermia and non-invasive 3D thermometry systems. It is anticipated that this confluence of technology will facilitate the wider implementation of medicines in clinical practice by improving their translational relevance.

## Abbreviations

### Coatings

TMSMA: 3-(Trimethoxysilyl)propyl methacrylate
PEGMA: Poly(ethylene glycol) methyl ether methacrylate
PEI: Polyethylenimine
PAA: Poly(acrylic acid)
PMAO: Poly(maleic anhydride-*alt*-1-octadecene)
PEG: Polyethyleneglycol
PVA: Poly(vinyl alcohol)
SAS: (3-Triethoxysilyl)propylsuccinic anhydride
PG: Polyglycerol
PMAA: Poly(methacrylic acid)
PPEG: Poly(oxy-1,2-ethanediyl)R-(3-phosphonopropy)ω-hydroxyl
PEO: Poly(ethylene oxide)
DOPA: Dihydroxy-l-phenylalanine
PLA: Poly(lactic acid)
TREG: Triethylene glycol
OA: Oleic acid
APS: Aminopropyl silane

### Media

PBS: Phosphate buffer saline
HEPES: 4-(2-Hydroxyethyl) piperazine-1-ethanesulfonic acid
FBS: Fetal bovine serum
FCS: Fetal calf serum
DMEM: Dulbecco's modified Eagle medium
RPMI: Roswell Park Memorial Institute medium

### Experimental techniques

TEM: Transmission electron microscopy
DLS: Dynamics light scattering
SEM: Scanning electron microscopy
FTIR: Fourier transform infrared spectroscopy
ATR-FTIR: Attenuated total reflection FTIR
TGA: Thermogravimetric analysis
EDS: Energy dispersive spectroscopy
BCA: Bicinchoninic acid assay
DCS: Differential centrifugal sedimentation
SEC-FPLC: Size exclusion chromatography/fast protein liquid chromatography
HPLC: High performance liquid chromatography
1D-SDS-PAGE: One dimensional sodium dodecyl sulfate-polyacrylamide gel electrophoresis
2D-PAGE: Two dimensional-polyacrylamide gel electrophoresis
LC-MS: Liquid chromatography-mass spectrometry
LC-MS/MS: Liquid chromatography-mass spectrometry/mass spectrometry
RPLC-MS/MS: Reversed phase liquid chromatography-mass spectrometry/mass spectrometry
Nano-LC-ESI-MS/MS: Nano-liquid chromatography-electro spray ionization-mass spectrometry/mass spectrometry

## Data availability

No primary research results, software or code have been included and no new data were generated or analyzed as part of this review.

## Author contributions

C. Jacinto: conceptualization, funding acquisition, methodology, project administration, supervision, writing – review and editing. Yasir Javed: data curation, writing original draft. Gabriel Lavorato: conceptualization, methodology, project administration, writing original draft. Wilson A. Tarraga: data curation, writing original draft. Blessed Isaac C. Conde: data curation, writing original draft. Juan Manuel Orozco: data curation, writing original draft. Agustin S. Picco: conceptualization, methodology, project administration, writing original draft. J. Garcia: conceptualization, methodology, project administration, writing original draft. Carlos Sato Baraldi Dias: data curation, writing original draft. S. Malik: conceptualization, funding acquisition, methodology, project administration, supervision, writing – review and editing. S. K. Sharma: conceptualization, funding acquisition, methodology, project administration, supervision, writing – review and editing. All authors have contributed to original draft writing and revision of the manuscript.

## Conflicts of interest

There are no conflicts to declare.
